# A Real‐Data‐Driven Framework for Evaluating Differential Transcript Usage Methods Across Long‐Read Bulk, Single‐Cell, and Spatial Transcriptomics

**DOI:** 10.1002/advs.77756

**Published:** 2026-09-17

**Authors:** Chenxing Zhang, Jun Liu, Qi Zhao, Huilong Yin, Angang Yang, Minhua Zheng, Rui Zhang

**Affiliations:** ^1^ State Key Laboratory of Holistic Integrative Management of Gastrointestinal Cancers and Department of Immunology Fourth Military Medical University Xi'an Shaanxi China; ^2^ State Key Laboratory of Holistic Integrative Management of Gastrointestinal Cancers and Department of Biochemistry and Molecular Biology Fourth Military Medical University Xi'an Shaanxi China; ^3^ Henan Collaborative Innovation Center of Molecular Diagnosis and Laboratory Medicine School of Medical Technology Xinxiang Medical University Xinxiang Henan China; ^4^ State Key Laboratory of Holistic Integrative Management of Gastrointestinal Cancers and Department of Medical Genetics and Developmental Biology Fourth Military Medical University Xi'an Shaanxi China

**Keywords:** differential transcript usage (DTU), evaluation framework, evaluation metric, long‐read transcriptomics, multi‐omics data resources

## Abstract

Differential transcript usage (DTU) analysis reveals transcript‐level regulation in alternative splicing. With the rapid adoption of long‐read sequencing in bulk, single‐cell, and spatial transcriptomics, reliable evaluation of DTU methods under real biological conditions becomes essential. Current evaluation frameworks mainly rely on simulated data, which can introduce bias and may not reflect true regulatory mechanisms. A real‐data‐driven framework is constructed to evaluate DTU methods across long‐read bulk, single‐cell, and spatial transcriptomics. The framework includes two key components. First, a biologically grounded reference transcript set is defined using RNA‐binding protein (RBP) knockout or knockdown RNA‐seq data together with experimentally validated RBP‐transcript interactions. Second, a DTU‐specific evaluation metric, the transcript set enrichment score, is introduced to quantify how effectively a method prioritizes reference transcripts in ranked results. The framework is systematically validated for reliability, unbiasedness, stability, effectiveness, and robustness using multiple real RNA‐seq datasets. Supported by this validation, ten representative DTU methods are evaluated across long‐read and short‐read data, revealing performance differences across data types. Beyond evaluating DTU methods, the framework is further extended to predict transcript‐level RBP activity, recovering perturbed RBPs more consistently than gene‐level differential expression strategies. Together, this study establishes a biologically interpretable and data‐driven standard for DTU method evaluation.

## Graphical abstract

Grounded in real biological RNA‐seq data, the framework benchmarks ten differential transcript usage (DTU) methods across short‐read bulk, long‐read bulk, long‐read single‐cell, and long‐read spatial transcriptomics. By integrating reference transcript sets with ranked DTU results, the Transcript Set Enrichment Score (TSES) identifies data‐type‐specific method strengths and supports transcript‐level RNA‐binding protein activity prediction, enabling biologically informed interpretation of isoform regulation.

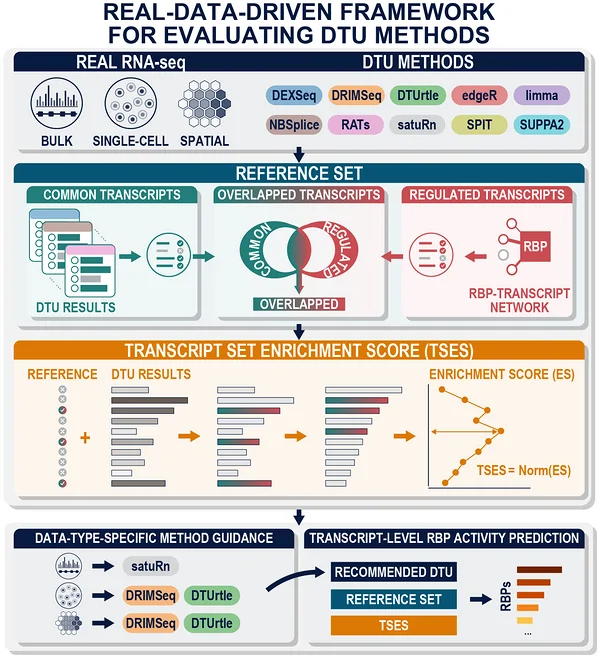

## 1 Introduction

**FIGURE 1:**
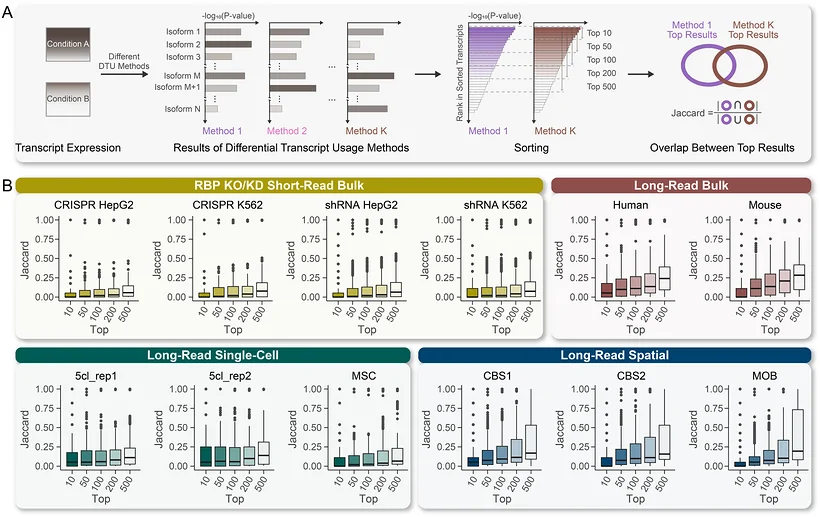
Overlap among the results of DTU methods. (A) Workflow for measuring the overlap among DTU method results. Transcript expression data under different conditions are used as input for ten representative DTU methods. Each method produces a ranked list of significant transcripts. For any two methods, we rank transcripts by significance, select the top transcripts from each list, and calculate their overlap using the Jaccard index. (B) Quantitative comparison of overlaps among DTU method results across datasets. Different box colors represent different data types, including RBP KO/KD short‐read bulk RNA‐seq data, as well as long‐read bulk RNA‐seq, long‐read single‐cell RNA‐seq, and long‐read spatial RNA‐seq data without KO/KD. Each data type contains several datasets; for example, the long‐read spatial type includes CBS1, CBS2, and MOB. Each dataset includes multiple biological conditions (for example, different spatial regions), which are paired to form condition pairs. Each point in the boxplot represents the Jaccard index between two DTU methods under one condition pair at a given top threshold. The x‐axis shows the top thresholds based on significance in DTU results, and the y‐axis shows the Jaccard index of significant transcripts between two methods.

Differential transcript usage (DTU) methods are used to measure changes in the expression proportion of transcripts from the same gene under different biological conditions [1, 2]. Because these transcript‐level changes are mainly regulated by alternative splicing [3, 4], DTU analysis gives important insights into biological mechanisms and disease processes from an alternative splicing perspective [2, 5]. Existing DTU methods are based on different statistical models, such as negative binomial regression, Dirichlet–multinomial modeling, and generalized linear models (for example, DEXSeq [6], DRIMSeq [7], DTUrtle [8], and satuRn [9]). Some other methods use non‐parametric tests, such as SUPPA2 [10] and SPIT [11]. In addition, these methods may also be limited by data type characteristics and original design scope. In short‐read RNA‐seq, read‐to‐transcript ambiguity (RTA) caused by overlapping transcripts is not directly modeled by most DTU methods and can affect both power and false discovery rate (FDR) control [12]. In long‐read bulk, single‐cell, and spatial transcriptomics, many DTU methods are applied outside their original design scope, where variable transcript coverage, sparsity, and cell‐ or region‐level heterogeneity can further change method performance [13]. These differences in modeling and data context often lead to inconsistent results when the same dataset is analyzed by different DTU methods. As shown in Figure 1, overlap among the results produced by different DTU methods is limited, with median pairwise Jaccard indices generally below 0.1 across several data types and remaining below 0.25 even at broader top‐ranked thresholds. Although false‐positive discoveries may contribute to disagreement among methods, sample‐label permutation analyses indicate that they do not fully account for the generally low overlap among DTU methods (Figure S1). These observations highlight the need for a systematic and objective framework to evaluate performance differences and select methods appropriate for different data types.

However, most current DTU evaluation studies still depend on simulated datasets and classification evaluation metrics [14, 15]. For instance, Merino et al. used simulated data generated by RSEM, where real RNA‐seq datasets were only used to estimate simulation parameters. Their evaluation relied on standard metrics such as precision, recall, and F1‐score [14]. Likewise, Lio et al. carried out a large‐scale benchmark across bulk, single‐cell, and time‐series datasets, including real data such as SARS‐CoV‐2 infection and prostate cancer. But their main evaluation still used simulated DTU events as the gold standard. Simulated data, however, are based on manually defined distributions and cannot reflect the full biological complexity of transcript regulation in real tissues or diseases [15]. As a result, the evaluation results are often biased toward simulation assumptions, and their usefulness for real datasets remains unclear. In addition, these metrics, though widely used in classification studies, are not designed for DTU evaluation. They only check whether a DTU method can “hit” the reference transcript set (the so‐called gold standard). In contrast, DTU analysis focuses on whether a method can prioritize the reference transcripts in its ranked results. Therefore, current DTU evaluation frameworks still lack reliable reference transcript sets and DTU‐specific evaluation metrics.

To solve these problems, this study proposes an evaluation framework based on real biological data. This framework has two key parts: (1) building a reference transcript set from real biological data, and (2) designing a DTU‐specific evaluation metric. To build the reference transcript set, we note that transcript‐level changes mainly come from alternative splicing, which is regulated by RNA‐binding proteins (RBPs) [16, 17]. We integrate transcript expression data from RBP knockout or knockdown (KO/KD) experiments with known RBP‐transcript regulatory relationships, and then combine the consensus results from several DTU methods. This process produces a high‐confidence reference transcript set that is supported by both biological and methodological evidence. For the metric design, we develop the transcript set enrichment score (TSES), which measures how well a method prioritizes the reference transcript set at the top of its ranked list. In this way, DTU accuracy is evaluated from a prioritization view rather than a simple hit‐or‐miss one, giving a more specific and biologically meaningful result. Then, we validate both the reference transcript set and the TSES metric using diverse datasets, confirming that the reference transcript set is reliable and that the TSES metric is unbiased, stable, and effective.

Using this framework, RBP KO/KD short‐read bulk RNA‐seq datasets, together with non‐KO/KD long‐read bulk RNA‐seq, long‐read single‐cell RNA‐seq, and long‐read spatial RNA‐seq datasets, are collected to systematically evaluate ten representative DTU methods. The evaluation results provide clear guidance for method selection across different data types. satuRn achieves the best accuracy for bulk RNA‐seq data, while DRIMSeq and DTUrtle perform better for long‐read single‐cell RNA‐seq and long‐read spatial RNA‐seq datasets. These results indicate that DTU methods have data‐type‐dependent strengths.

Finally, the evaluation results and components of the framework are further combined to predict RBP activity at the transcript level. Specifically, the recommended DTU method is combined with RBP‐specific overlapped transcript sets and TSES. In the evaluated ENCODE and GEO datasets, and within the present reference‐set and scoring framework, this downstream application recovers perturbed RBPs more consistently than the evaluated DTE + TSES and gene‐level DGE strategies.

In summary, this study builds a real‐data‐based framework for evaluating DTU methods. It provides both a reliable reference transcript set and a DTU‐specific metric that make the evaluation more accurate and biologically meaningful. The framework offers practical guidance for method selection across different data types and can be extended to predict RBP activity at the transcript level. Together, these advances promote a more reliable interpretation of transcript‐level regulation in complex biological systems.

## 2 Results

### 2.1 DTU Methods Show Limited Agreement in Top‐Ranked Transcripts Across Data Types

We first assess the agreement among ten DTU methods across datasets generated using different sequencing platforms. For each dataset, we obtain the ranked transcript list produced by each method and calculate pairwise Jaccard indices between methods for the top 10, 50, 100, 200, and 500 transcripts to quantify the overlap between their results (Figure 1).

The results show consistently low agreement among methods across both short‐ and long‐read sequencing platforms and across bulk, single‐cell, and spatial RNA‐seq data. For example, in the human long‐read bulk RNA‐seq dataset, the median pairwise Jaccard indices for the top 10 transcripts range from approximately 0 to 0.05 across the evaluated contrasts. Even when the comparison is expanded to the top 500 transcripts, the median Jaccard index reaches only approximately 0.24. Agreement is even lower in long‐read single‐cell and spatial RNA‐seq datasets, with median Jaccard indices often below 0.1 (Figure 1; Table S1).

Given this consistently low overlap, we next investigate whether it can be explained by false‐positive discoveries. We estimate the empirical FDR of each method using sample‐label permutations (see Materials and Methods for details). The mean empirical FDR across all datasets is 1.62% for DEXSeq, 4.34% for DRIMSeq, 4.30% for DTUrtle, 4.51% for edgeR‐diffSplice, 1.65% for limma‐diffSplice, 1.83% for NBSplice, 72.13% for RATs, 1.25% for satuRn, <0.01% for SPIT, and 0.05% for SUPPA2 (Figure S1 and Table S2). Thus, nine of the ten evaluated methods have mean empirical FDR values below 5%, although the values vary across individual datasets and contrasts.

Collectively, these results suggest that false‐positive discoveries alone cannot account for the disagreement among DTU methods. Instead, method‐specific differences in statistical modeling and transcript prioritization are also likely to contribute to differences in the transcripts detected from the same dataset, potentially leading to divergent biological interpretations and conclusions. These findings therefore highlight the need for a systematic and fair evaluation framework to compare DTU methods and identify those best suited to different data types.

### 2.2 Overview of a Real‐Data‐Driven Framework for DTU Method Evaluation

**FIGURE 2:**
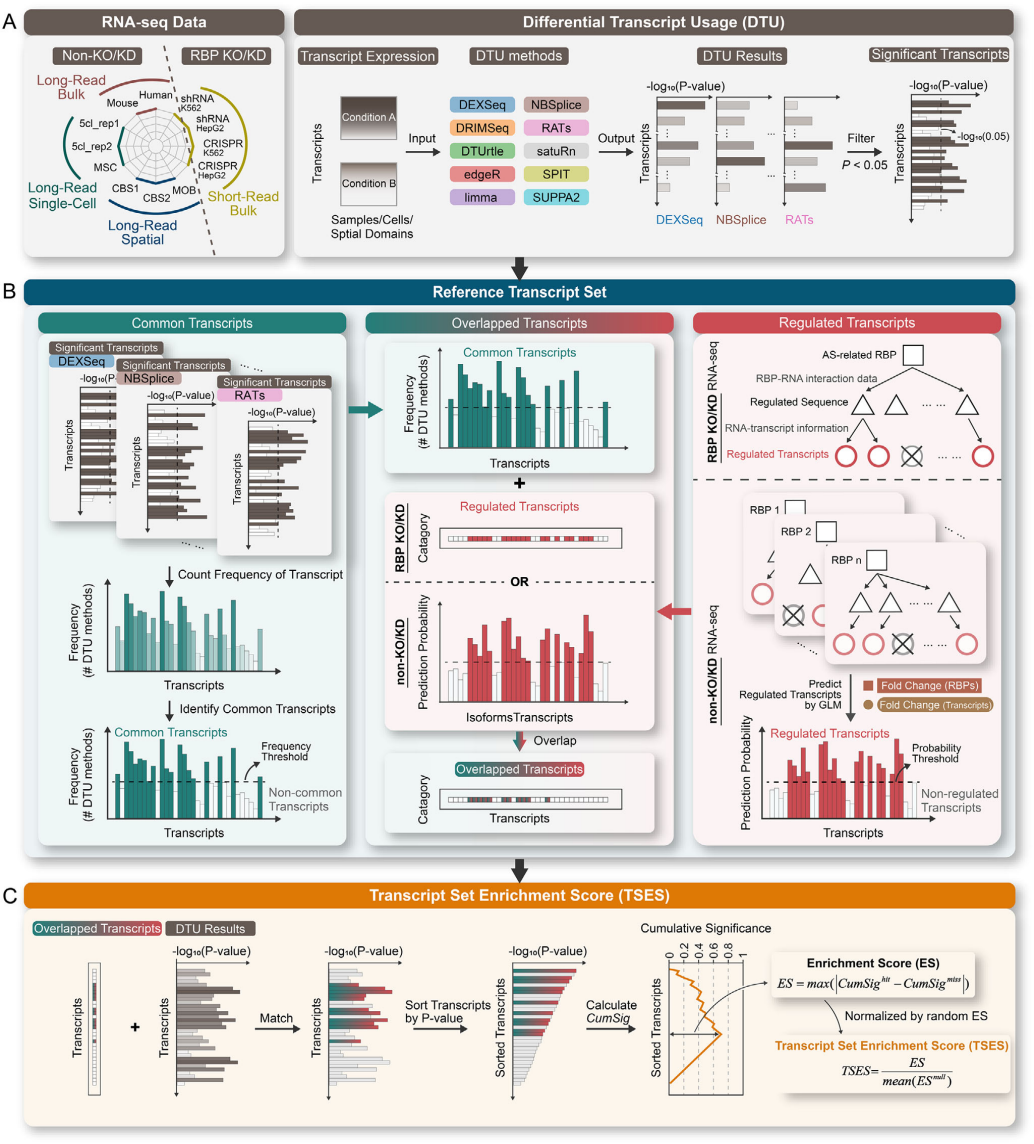
Framework for evaluating DTU methods. (A) Collection of datasets and DTU methods. It includes short‐read bulk RNA‐seq datasets from RBP KO/KD experiments, as well as long‐read bulk RNA‐seq, long‐read single‐cell RNA‐seq, and long‐read spatial RNA‐seq datasets without KO/KD. Ten representative DTU methods are evaluated. (B) Construction of a reference transcript set based on real biological data. It includes the common transcript set (left panel), the regulated transcript set (right panel), and the overlapped transcript set (middle panel). (C) Design of a DTU‐specific evaluation metric. The transcript set enrichment score (TSES) is designed to evaluate the enrichment of the reference transcript set along a transcript list ranked by P‐value in a DTU result. The score is then normalized using random permutations.

**FIGURE 3:**
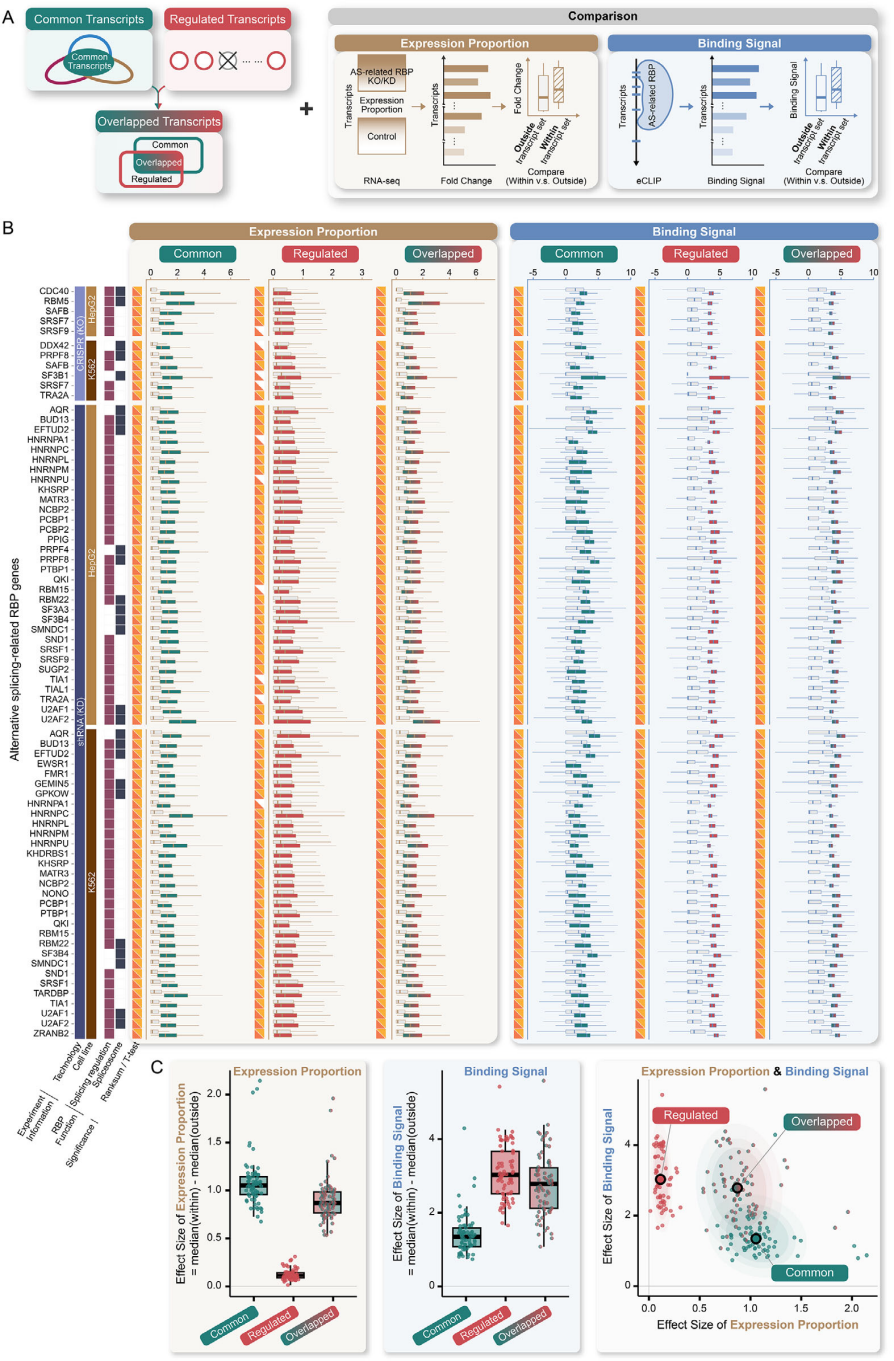
Quantitative validation of reference transcript sets. The reliability of the common, regulated, and overlapped transcript sets is tested by comparing transcripts within and outside each reference set in RBP KO/KD datasets. (A) Workflow for evaluating reference transcript sets. The common transcript set, regulated transcript set, and overlapped transcript set are compared with transcripts outside the corresponding set. The comparison is based on expression proportion fold changes between RBP KO/KD and control groups and binding signals. (B) Statistical validation of common, regulated, and overlapped transcript sets. In each boxplot, colored boxes represent transcripts within the corresponding set, while grey boxes represent transcripts outside the set. The y‐axis represents RBP KO/KD datasets, and the x‐axis shows expression proportion fold change or binding signal. Annotation bars indicate experimental technology, cell line, RBP Function, and Significance from t‐test and rank‐sum test. (C) Effect size of expression proportion fold change and binding signal. Effect size is calculated as median (within) minus median (outside) for each RBP KO/KD dataset and each reference transcript set. Boxplots summarize effect size values, and the scatter plot shows the relationship between the effect size of expression proportion fold change and the effect size of binding signal.

To overcome the problems of data dependency and metric limitation in existing evaluations, we propose a real‐data‐based framework for DTU method evaluation (Figure 2). This framework includes three parts: (1) the collection of datasets and DTU methods (Figure 2A); (2) the construction of a reference transcript set based on real biological data (Figure 2B), and (3) the design of a DTU‐specific evaluation metric (Figure 2C).

For dataset collection, we follow two main criteria: (1) Each sequencing type should include multiple tissues or cell lines from the same experimental source and (2) Long‐read single‐cell RNA‐seq and long‐read spatial RNA‐seq datasets should include cell‐type or spatial‐domain annotations. Following these criteria, the framework integrates RBP KO/KD short‐read bulk RNA‐seq datasets (CRISPR_HepG2, CRISPR_K562, shRNA_HepG2 and shRNA_K562), long‐read bulk RNA‐seq datasets (Human and Mouse), long‐read single‐cell RNA‐seq datasets (5cl_rep1, 5cl_rep2 and MSC) and long‐read spatial RNA‐seq datasets (CBS1, CBS2 and MOB) (left panel of Figure 2A). Detailed information about dataset collection and filtering is summarized in Table 1 and described in the MATERIALS AND METHODS section. For the collection of DTU methods, the framework evaluates methods according to three criteria: the method (1) takes transcript‐level input from two biological conditions; (2) focuses on transcript usage rather than gene‐level expression; and (3) is publicly available and easy to use. Following these criteria, the framework integrates ten representative DTU tools: DEXSeq, DRIMSeq, DTUrtle, edgeR‐diffSplice, limma‐diffSplice, NBSplice, RATs, satuRn, SPIT, and SUPPA2 (right panel of Figure 2A). Detailed information about the DTU methods collection is summarized in Table 3 and described in the MATERIALS AND METHODS section.

**TABLE 1:** Details of RNA‐seq Datasets.

Sequencing Technology	Experiment Type	Resource	Refs.	Detail
Short‐read Bulk	RBP KO/KD	ENCODE	[28, 29, 30]	376 RBP KO/KD experiments, including CRISPR knockout in HepG2 (CRISPR_HepG2), CRISPR knockout in K562 (CRISPR_K562), shRNA knockdown in HepG2 (shRNA_HepG2), and shRNA knockdowns in K562 (shRNA_K562).
Long‐read Bulk	Non‐KO/KD	ENCODE	[35]	9 human tissues and 7 human cell lines, and 6 mouse tissues and 2 mouse cell lines
Long‐read Single‐cell	Non‐KO/KD	GSE154868 GSE154869 GSE154870	[36]	H2228, H838, H1975, HCC827, A549 cell lines (5cl_rep1 and 5cl_rep2) and mouse quiescent muscle stem cell and activated stem cells (MSC).
Long‐read Spatial	Non‐KO/KD	GSE153859	[37]	two mouse hippocampal samples (CBS1, CBS2) and one mouse olfactory bulb sample (MOB).

For the construction of the reference transcript set (Figure 2B), we integrate consensus results from multiple DTU methods to find common transcripts (left panel of Figure 2B). Specifically, we obtain significant transcripts from each DTU result and record how often each transcript is detected as significant across all methods. Transcripts detected by at least half of the DTU methods are defined as common transcripts. At the same time, we combine real data from RBP KO/KD experiments with known RBP‐transcript regulatory relationships to identify regulated transcripts (right panel of Figure 2B). The identification of regulated transcripts includes two cases: (1) When RBP KO/KD RNA‐seq data are available, we use RBP‐RNA interaction data and RBP‐transcript information to identify transcripts regulated by alternative splicing (upper part of the right panel in Figure 2B). (2) For non‐KO/KD datasets, we build a generalized linear model to predict regulated transcripts. This model uses fold changes of RBP gene expression and transcript expression proportion between conditions as features, and uses regulated transcripts identified from RBP KO/KD data as training labels (lower part of the right panel in Figure 2B). The detailed model structure and training process are shown in Figure S2 and in the MATERIALS AND METHODS section. Finally, we take the intersection between the common transcript set and the regulated transcript set to obtain the overlapped transcript set (center panel of Figure 2B). These overlapped transcripts are supported by both DTU method consensus and biological regulation evidence, and they are used as the reference transcript set for method evaluation.

To evaluate DTU method accuracy from a “prioritize” rather than a “hit‐or‐miss” view, we design a new metric called the transcript set enrichment score (TSES) (Figure 2C). This metric is inspired by gene set enrichment analysis (GSEA), which tests whether a gene set is enriched at the top or bottom of a ranked gene list [18]. Similarly, TSES evaluates the enrichment of the reference transcript set along a transcript list ranked by *p*‐value in a DTU result and normalizes the score using random permutations. A higher TSES value means that most reference transcripts are ranked near the top, indicating that the DTU method better captures true expression differences between conditions. The detailed calculation steps and formulas of TSES are described in the MATERIALS AND METHODS section.

Together, this framework yields a biologically supported reference transcript set and a DTU‐specific accuracy metric, enabling systematic comparison of DTU methods across data types.

### 2.3 Reference Transcript Sets Reflect Genuine Transcript Usage Changes and RBP Binding Signals in RBP Perturbation Data

Because the reference transcript sets (regulated, common, and overlapped) are not perfect gold standards, their reliability requires validation. This analysis tests whether these sets represent transcripts with real biological differences between perturbation and control groups, including both transcript usage changes and RBP binding signals.

In RBP knockout or knockdown experiments, transcripts regulated by a perturbed RBP are expected to show larger expression proportion fold changes between perturbation and control samples than background transcripts. Therefore, the distribution of expression proportion fold changes is compared between transcripts within and outside each reference set. One‐sided t‐tests and Wilcoxon rank‐sum tests are used to assess whether transcripts within the reference sets show higher values (Figure 3A). The results show that, in most datasets, transcripts within the regulated, common, or overlapped sets have significantly higher fold changes than transcripts outside the corresponding sets (*p* < 0.05 for both tests; Figure 3B; Table S3). For the regulated transcript set, 64 of 74 datasets pass the t‐test, and all 74 datasets pass the rank‐sum test. For the common and overlapped transcript sets, all datasets pass both tests.

RBP binding signal provides an additional layer of biological validation. For each RBP perturbation dataset with matched ENCODE RBP binding signal data, transcripts are assigned an RBP binding signal score, defined as the mean RBP binding signal across available replicates. The RBP binding signal scores are then compared between transcripts within and outside each reference set using the same one‐sided t‐test and Wilcoxon rank‐sum test framework (Figure 3A). Transcripts within the regulated, common, and overlapped sets show stronger RBP binding signals than background transcripts, indicating that the reference sets are enriched not only for transcript usage changes but also for independent RBP‐binding evidence supporting RBP‐transcript association (Figure 3B; Table S3). This validation based on RBP binding signal is further extended to the predicted regulated transcript sets. In five‐fold cross‐validation, fold‐specific predicted regulated transcripts show higher RBP binding signals than transcripts outside the corresponding predicted sets, supporting the cross‐validation consistency of the regulated transcript prediction model (Figure S3 and Table S3). In addition, independent RBP knockdown datasets from the GEO database are used to evaluate the external generalizability of the predicted regulated transcript sets. For MATR3, RBM5, PCBP1, and TRA2A, predicted regulated transcripts show stronger RBP binding signals than background transcripts using matched ENCODE RBP binding signal data, further supporting the biological reliability of the regulated transcript prediction model (Figure S4 and Table S3).

To summarize these two types of evidence, expression effect and RBP binding signal effect are calculated as median(within) minus median(outside) for each dataset and each reference set. Positive expression effects indicate stronger transcript usage changes within the reference sets, while positive RBP binding signal effects indicate stronger RBP binding signal enrichment. The paired effect analysis shows that the reference transcript sets are supported by both expression proportion changes and RBP binding signals (Figure 3C). Among the three candidate reference sets, the overlapped transcript set shows the most favorable combined effect size and is therefore selected as the main reference set for downstream evaluation. The common transcript set shows the strongest expression effect but a relatively modest RBP binding signal effect (1.0537 and 1.3446, respectively), whereas the regulated transcript set shows only a weak expression effect but the strongest RBP binding signal effect (0.1136 and 3.0292, respectively). In contrast, the overlapped transcript set retains a pronounced expression effect while also showing strong enrichment in RBP binding signal, with effect sizes of 0.8708 and 2.7847, respectively. This set therefore integrates the statistically significant enrichment in RBP binding signal captured by the regulated transcript set with the pronounced transcript proportion changes captured by the common transcript set. Thus, the overlapped transcript set provides a higher‐confidence and biologically supported reference than either component set alone (Figure 3C; Table S3).

The same validation strategy is also applied in the leave‐one‐method‐out setting to address the potential circularity caused by using DTU method consensus during reference set construction. In this analysis, one DTU method is excluded at a time, and the common and overlapped transcript sets are rebuilt using the remaining methods. The rebuilt reference sets remain supported by both expression proportion changes and RBP binding signals, indicating that the validation results are not driven by any single DTU method included in the reference set construction (Figure S5 and Table S3).

Together, these findings indicate that the regulated, common, and overlapped transcript sets capture genuine biological signals in RBP perturbation data. Transcripts within these reference sets show stronger transcript usage changes and higher RBP binding signals than background transcripts, and these patterns remain supported by cross‐validation, independent GEO knockdown validation, and leave‐one‐method‐out analyses. Among the three candidate sets, the overlapped transcript set provides the most balanced evidence by retaining the expression proportion advantage of the common transcript set and the RBP binding signal advantage of the regulated transcript set. Therefore, the overlapped transcript set is used as the primary biologically grounded reference set for downstream DTU method evaluation.

### 2.4 TSES Provides an Unbiased, Stable, and Effective Metric for DTU Evaluation

**FIGURE 4:**
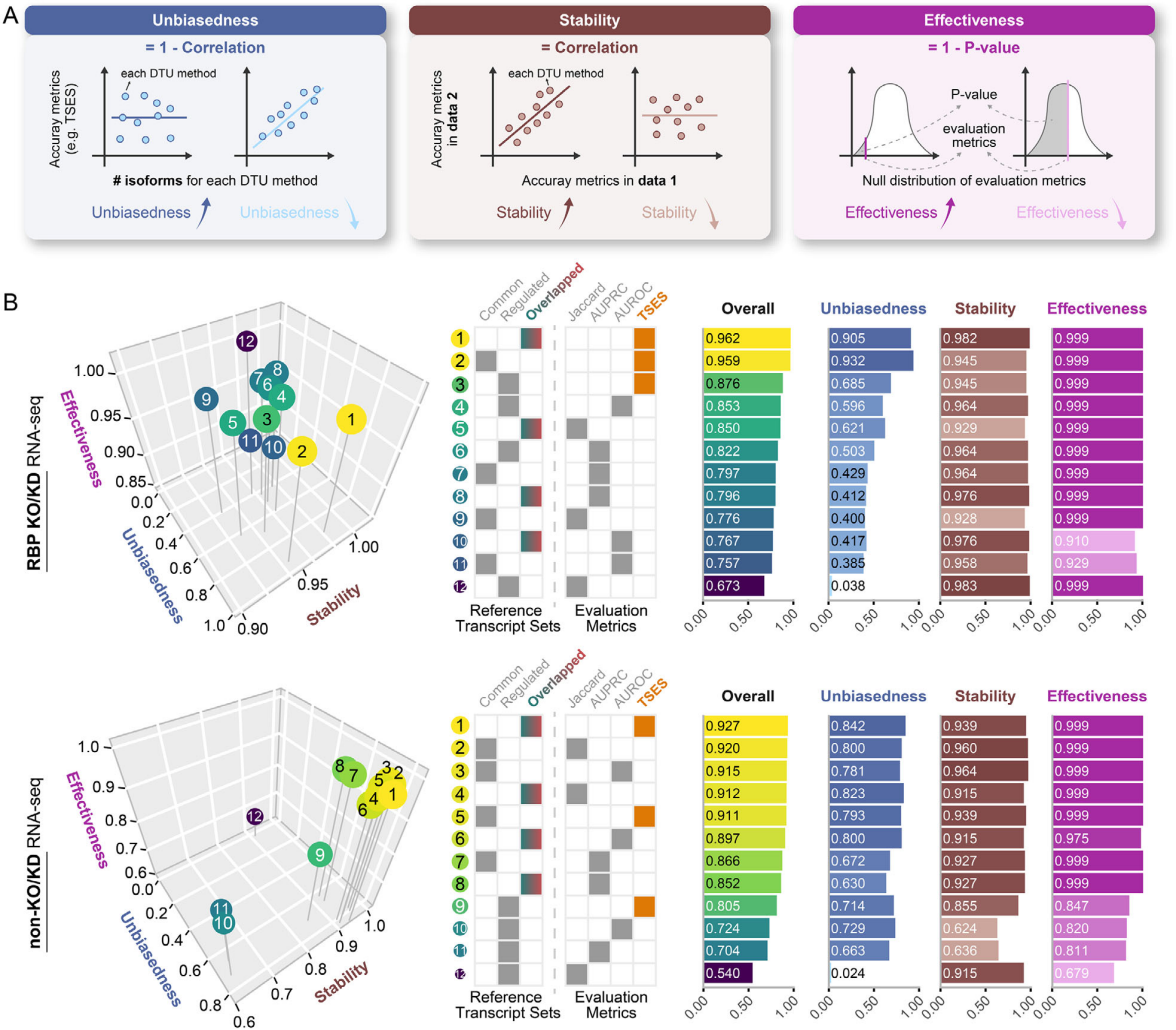
Quantification of unbiasedness, stability, and effectiveness of evaluation metrics. (A) Schematic overview of how unbiasedness, stability, and effectiveness are quantified. Unbiasedness is defined as one minus the correlation between the number of significant transcripts predicted by each DTU method and its accuracy score. Stability measures the correlation of scores for the same DTU method across different datasets. Effectiveness evaluates how well a metric distinguishes real accuracy from random noise by comparing real scores with those from random permutations. (B) Quantitative comparison of unbiasedness, stability, and effectiveness across different evaluation metrics. The left panel shows a 3D plot, where the x‐, y‐, and z‐axes represent unbiasedness, stability, and effectiveness, respectively. Each point indicates the scores of these three metrics under a specific combination of reference transcript set and evaluation metric. The middle panel displays an annotation bar showing the combination of each reference transcript set with its corresponding evaluation metric. The right panel presents the detailed scores of unbiasedness, stability, and effectiveness for each combination, together with the overall score, calculated as the average of the three. The upper and lower sections represent RNA‐seq data from different experimental conditions, including RBP knockout or knockdown (KO/KD) data and non‐KO/KD data.

We next evaluate whether TSES outperforms commonly used accuracy metrics in DTU evaluation. We assess each metric from three perspectives: unbiasedness, stability, and effectiveness. Unbiasedness measures whether the evaluation metric is independent of the number of predicted differential transcripts. In other words, the correlation between accuracy scores and the number of detected transcripts should be close to zero. Stability measures whether a metric gives consistent scores for the same DTU method across different datasets. A stable metric should show high correlation between scores from different datasets. Effectiveness tests whether a metric truly reflects the real performance of DTU methods. We test this by random permutation, where transcript names are shuffled to build a null model. If a metric is effective, the real score should be much higher than the score from the null model (Figure 4A; detailed calculation procedures are provided in the MATERIALS AND METHODS section).

To demonstrate the advantage of our proposed metric, TSES, we compare it with classical metrics including Jaccard, area under the precision–recall curve (AUPRC), and area under the receiver operating characteristic curve (AUROC). For each metric, we calculate unbiasedness, stability, and effectiveness using three different reference transcript sets. The results show that, across all reference sets, TSES achieves the highest unbiasedness, stability, and effectiveness. Moreover, the combination of the overlapped transcript set and TSES metric gives the best overall performance (0.962 in RBP KO/KD data and 0.927 in non‐KO/KD data, Figure 4B; Table S4).

Together with the validation of the reference transcript sets in the previous section, these results demonstrate that our framework provides reliable data and high‐quality metrics for the systematic evaluation of DTU methods. Overall, these analyses establish TSES, in combination with the overlapped transcript set, as a reliable standard for DTU method evaluation.

### 2.5 Comparative Performance of DTU Methods Across Bulk, Single‐Cell, and Spatial Data

**FIGURE 5:**
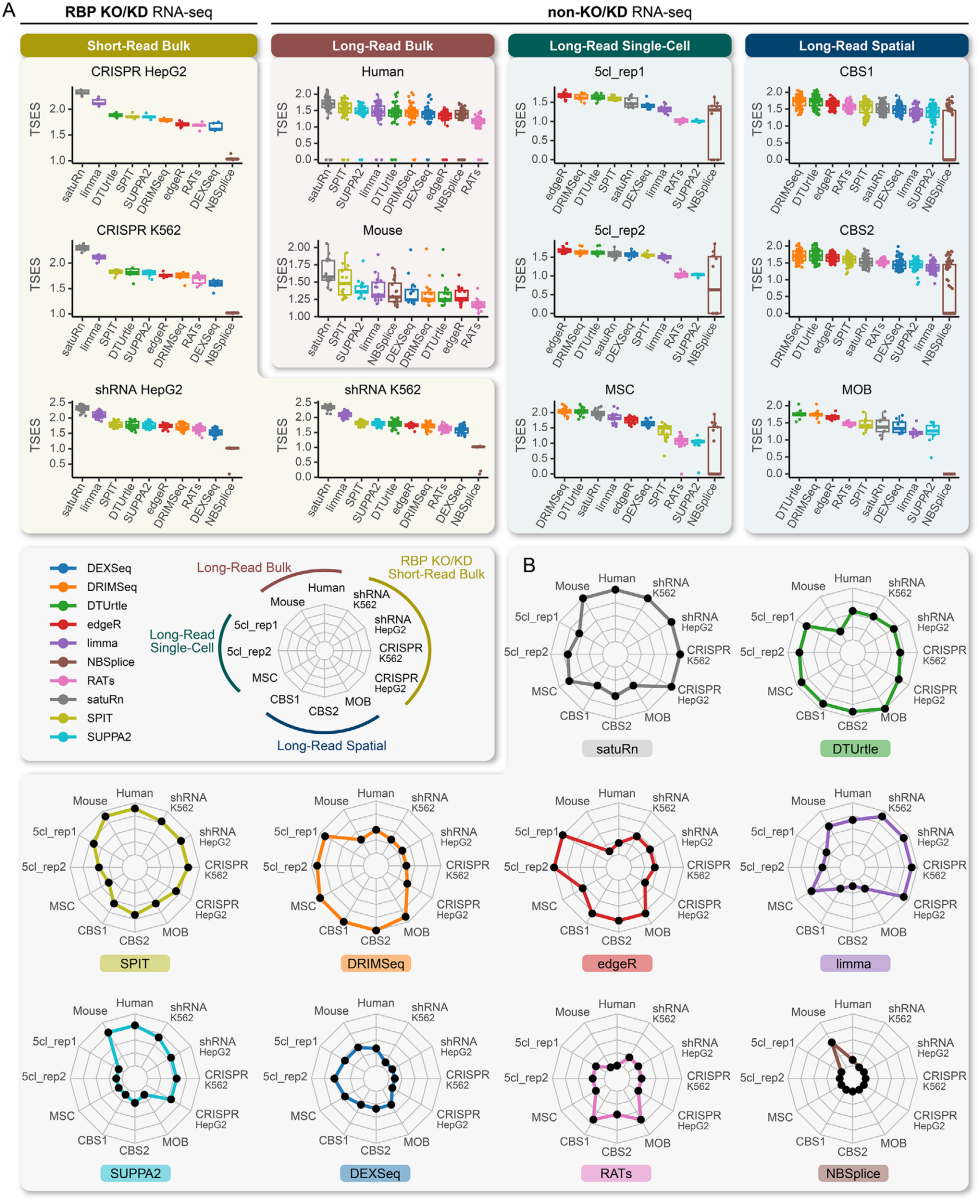
Evaluation results of ten representative DTU methods. (A) TSES of different DTU methods across multiple datasets. The y‐axis represents the TSES values, and the x‐axis shows the DTU methods. Each dot in the boxplot indicates the TSES value of one condition pair within a dataset. Methods on the x‐axis are ranked by their mean TSES values. (B) Ranking of DTU methods based on TSES in different datasets. In the radar chart, a point further away from the center indicates a higher ranking of the corresponding method in that dataset.

The evaluation framework based on TSES is applied to ten representative DTU methods across RBP KO/KD short‐read bulk RNA‐seq datasets and non‐KO/KD long‐read bulk RNA‐seq, long‐read single‐cell RNA‐seq, and long‐read spatial RNA‐seq datasets (Figure 5A; Table S5). Each method is ranked by mean TSES within each dataset, and the resulting ranks are summarized with radar charts (Figure 5B).

Clear performance differences are observed among DTU methods. In bulk RNA‐seq data (short‐read and long‐read), satuRn consistently shows the highest TSES scores across both short‐read bulk RNA‐seq and long‐read bulk RNA‐seq datasets. limma‐diffSplice ranks second in short‐read bulk RNA‐seq data, whereas SPIT ranks second in long‐read bulk RNA‐seq data. In long‐read single‐cell RNA‐seq and spatial RNA‐seq data, DRIMSeq, DTUrtle, and edgeR‐diffSplice generally rank among the strongest methods (Figure 5B). A leave‐one‐method‐out (LOMO) analysis yields similar TSES distributions and method rankings after each evaluated method is excluded from construction of its own reference transcript set, suggesting limited influence of direct self‐contribution (Figures S6–S8 and Table S5).

These results support method selection according to data type. satuRn, limma‐diffSplice, and SPIT are strong choices for bulk RNA‐seq data, whereas DRIMSeq, DTUrtle, and edgeR‐diffSplice perform better for long‐read single‐cell RNA‐seq or long‐read spatial RNA‐seq data, where sparsity and noise are more prominent.

### 2.6 Robustness of the Evaluation Framework to Model and Parameter Choices

The evaluation framework is tested for robustness to model choice, TSES parameter selection, reference set construction, common transcript threshold, transcript quantification method, expression filtering, and sample size. These analyses address potential sensitivity caused by model assumptions, circularity in reference set construction, upstream quantification, filtering strategy, and small‐sample settings.

**FIGURE 6:**
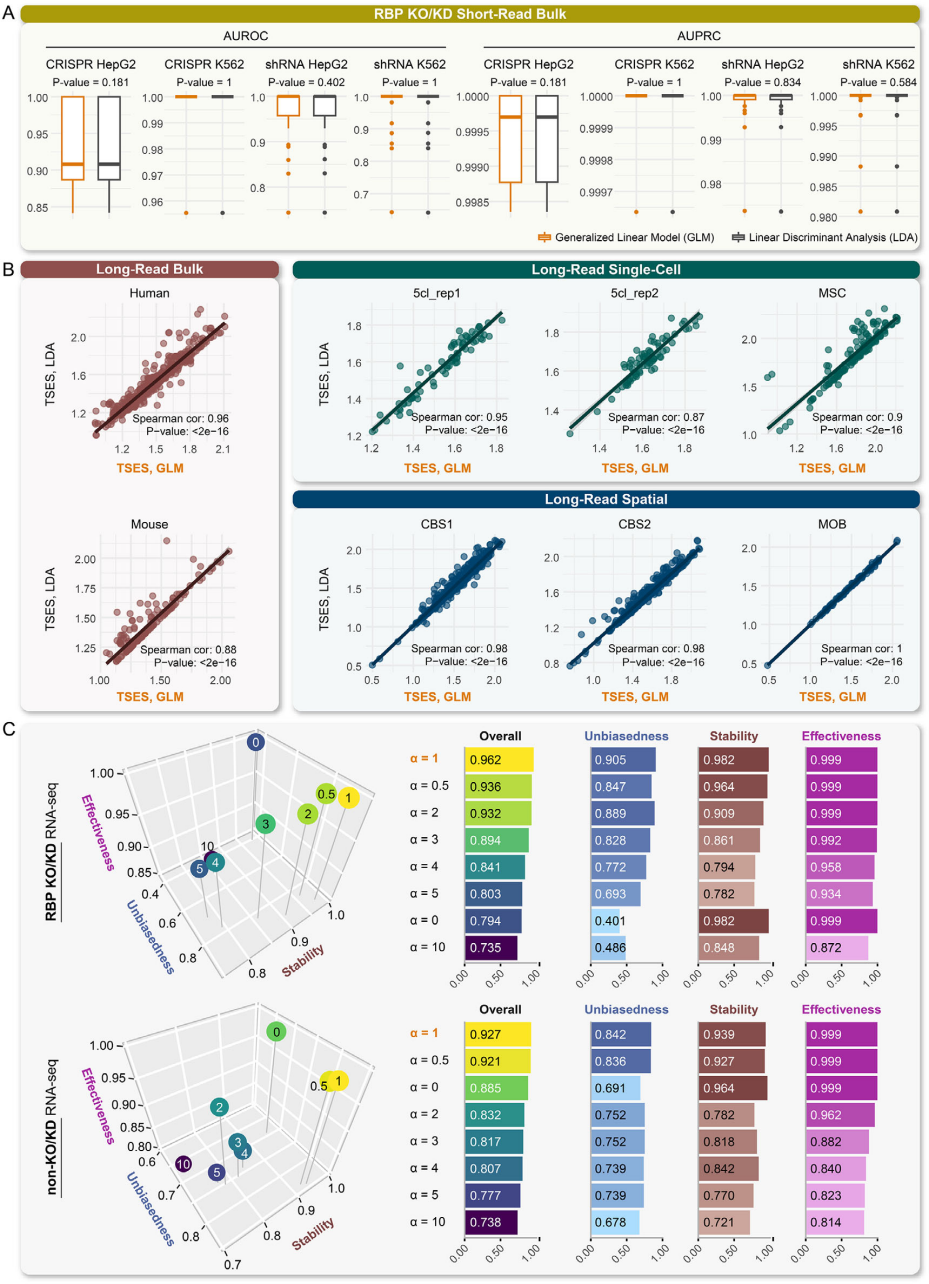
Parameter sensitivity analysis of the evaluation framework. (A) Similarity in the accuracy of different models for predicting regulated transcripts. The yellow box represents the generalized linear model, and the black box represents linear discriminant analysis. The y‐axis shows accuracy metrics; the four boxplots on the left use area under the precision‐recall curve (AUPRC), and the four on the right use area under the receiver operating characteristic curve (AUROC). Statistical significance is tested using a paired t‐test. (B) Similarity of TSES scores based on reference transcript sets obtained from different prediction models. The x‐axis shows TSES values calculated from reference transcript sets predicted by the generalized linear model (GLM), and the y‐axis shows those predicted by linear discriminant analysis (LDA). Similarity is measured using Spearman correlation and its significance. Different colors represent different data types: dark red for long‐read bulk RNA‐seq data, dark green for long‐read single‐cell RNA‐seq data, and dark blue for long‐read spatial RNA‐seq data. (C) Effect of the parameter α in the TSES metric. The left panel shows a 3D plot of unbiasedness, stability, and effectiveness for different α values (0, 0.5, 1, 2, 3, 4, 5, and 10), while the right panel presents the corresponding numerical scores. The upper and lower sections represent RNA‐seq data from different experimental conditions, including RBP knockout or knockdown (KO/KD) data and non‐KO/KD data.

First, the performance of two prediction models for regulated transcripts, generalized linear model (GLM) and linear discriminant analysis (LDA), is compared. No significant difference in prediction accuracy is observed between the two models across multiple RBP KO/KD short‐read bulk RNA‐seq datasets, as measured by AUPRC and AUROC (paired *t*‐test, *p* > 0.05; Figure 6A; Table S6). In addition, TSES scores calculated from reference transcript sets generated by the two models are highly correlated across all data types (Spearman r > 0.87, *p* < 2 × 10^−16^; Figure 6B; Table S6). These results indicate that the choice between GLM and LDA has limited influence on the evaluation results. GLM is retained as the default model because it provides a flexible framework for modeling transcript expression features.

The effect of the parameter α in TSES is further evaluated using α values of 0, 0.5, 1, 2, 3, 4, 5, and 10. TSES achieves the highest overall performance across unbiasedness, stability, and effectiveness at α = 1 (0.962 in RBP KO/KD data and 0.927 in non‐KO/KD data; Figure 6C; Table S6). Performance remains relatively stable when parameter α ranges from 0.5 to 2, indicating that the metric is not strongly dependent on a narrow parameter choice. Therefore, α = 1 is used as the default value in subsequent analyses.

To examine potential circularity caused by using DTU method consensus to construct the common transcript set, a leave‐one‐method‐out (LOMO) analysis is performed. In this setting, the method being evaluated is excluded from the construction of the corresponding reference transcript set. The common transcript set is reconstructed using the remaining DTU methods, and the overlapped transcript set is updated accordingly. DTU method evaluation results and method rankings under the LOMO setting remain similar to the original results (Figure S6, Table S5). Rank robustness analysis further shows that observed rank correlations between the original and LOMO settings are higher than random rank correlations generated by permutation (one‐sided Wilcoxon rank‐sum test *p* < 0.05 for all datasets; Figure S7). TSES scores based on LOMO reference sets are also consistent with those based on the original reference sets (Spearman r > 0.92, *p* < 2 × 10^−20^ for all datasets; Figure S8). These results indicate that the evaluation is not driven by any single DTU method contributing to the reference set.

The sensitivity of the common transcript threshold is assessed by varying the consensus threshold used to define common transcripts. In addition to the default threshold, where transcripts identified by at least five DTU methods are defined as common transcripts, alternative thresholds of at least three, four, six, and seven DTU methods are tested. DTU method performance remains comparable across these threshold settings (Figures S9–S12; Table S5), and rank stability relative to the default threshold remains higher than random expectation (one‐sided Wilcoxon rank‐sum test *p* < 0.001 for all datasets; Figure S13). Therefore, the main conclusions are not dependent on a single common transcript threshold.

The influence of transcript quantification methods is also evaluated. For short‐read bulk RNA‐seq datasets, alternative quantification results from Kallisto and Salmon are tested. For long‐read bulk RNA‐seq datasets, alternative quantification results from Bambu and Isoquant are tested. Within each quantification setting, all DTU methods use the same transcript expression data and preprocessing procedure, allowing method comparison under a consistent input condition. TSES distributions, method rankings, and rank stability remain broadly consistent between the original and alternative quantification methods (one‐sided Wilcoxon rank‐sum test *p* < 0.01 for all datasets; Figure S14 and Table S5). These results indicate that the framework is robust to upstream transcript quantification choices.

Expression filtering parameters are evaluated by testing six alternative “minExp” and “minSample” combinations: 2/2, 2/3, 3/2, 3/3, 5/2, and 5/3. Across these filtering settings, DTU method evaluation results remain stable (Figures S15–S20 and Table S5). Ranking robustness analysis shows that observed method rank correlations between the original and alternative filtering settings are higher than correlations from randomly permuted rankings (one‐sided Wilcoxon rank‐sum test *p* < 0.0001 for all datasets; Figure S21). Thus, the evaluation results are not mainly determined by a specific expression filtering rule.

Finally, robustness to sample size is assessed using random subsampling. DTU methods are evaluated under 3 versus 3 and 5 versus 5 sample settings, which represent common small‐sample settings in molecular biology studies. TSES‐based evaluation remains applicable under both subsampling settings (Figures S22 and S23 and Table S5), and method rankings remain more stable than random rankings when compared with the original setting (one‐sided Wilcoxon rank‐sum test *p* < 0.05 for most datasets; Figure S24). These results support the use of the framework in small‐sample settings.

In summary, the evaluation framework shows consistent performance across alternative model choices, TSES α values, LOMO reference construction, common transcript thresholds, transcript quantification methods, expression filtering parameters, and sample size settings. Together with the validation of reference transcript sets and the evaluation of TSES described above, these robustness analyses support the reliability of the framework for systematic DTU method evaluation.

### 2.7 Extension of the Framework Enables Transcript‐Level RBP Activity Prediction

**FIGURE 7:**
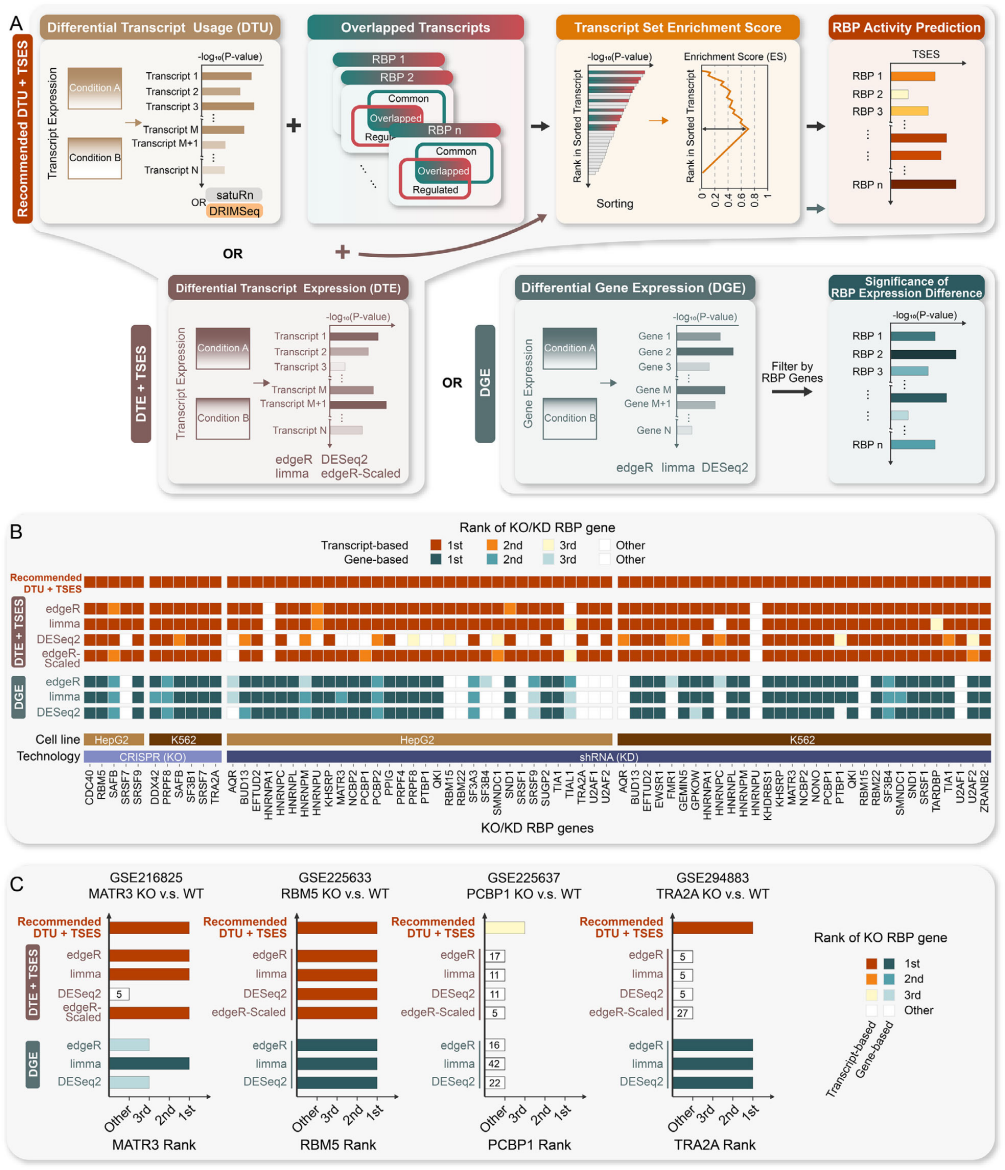
Workflow and validation of RBP activity prediction using DTU‐, DTE‐, and DGE‐based rankings. (A) Workflow of RBP activity prediction. DTU results are obtained using the DTU method recommended by the framework, including satuRn for bulk RNA‐seq data and DRIMSeq for long‐read single‐cell RNA‐seq or long‐read spatial RNA‐seq data. Alternatively, DTE results are generated using edgeR, limma, DESeq2, or edgeR‐Scaled. For each RBP, the overlapped transcript set constructed from common and regulated transcripts serves as the reference transcript set. TSES evaluates enrichment of the reference transcript set along transcripts ranked by ‐log_10_(P‐value), and RBPs are ranked by TSES. In the gene‐level comparison, DGE results from edgeR, limma, and DESeq2 are filtered to RBP genes and ranked by expression‐difference significance. (B) Validation using 74 ENCODE RBP KO/KD short‐read bulk RNA‐seq datasets, including 5 CRISPR knockout datasets in HepG2, 6 CRISPR knockout datasets in K562, 32 shRNA knockdown datasets in HepG2, and 31 shRNA knockdown datasets in K562. Columns represent KO/KD RBP genes grouped by cell line and technology. Rows represent recommended DTU + TSES, DTE + TSES, and DGE strategies. Tile colors indicate whether the KO/KD RBP gene ranks 1st, 2nd, 3rd, or lower than 3rd. (C) Validation using independent GEO case datasets, including MATR3, RBM5, PCBP1, and TRA2A knockout datasets. Bars indicate the rank category of the knocked‐out RBP gene for each transcript‐based or gene‐based strategy, and numbers mark ranks lower than 3rd. The recommended DTU + TSES ranks the knocked‐out RBP gene within the top three across all four independent datasets.

To further examine the application value of the evaluation framework, its evaluation results and components are combined for transcript‐level RBP activity prediction. Figure 7A summarizes the recommended DTU + TSES workflow and two comparison strategies: differential transcript expression (DTE) followed by TSES, hereafter DTE + TSES at the transcript level, and differential gene expression (DGE) at the gene level. In the recommended DTU + TSES workflow, DTU results are obtained using the DTU method recommended by the framework, including satuRn for bulk RNA‐seq and DRIMSeq for long‐read single‐cell RNA‐seq or long‐read spatial RNA‐seq data. In the DTE + TSES comparison, DTE results from edgeR [19], limma [20], DESeq2 [21], or edgeR‐Scaled [22] are used as transcript rankings for TSES. In the DGE comparison, DGE results from edgeR [19], limma [20], and DESeq2 [21] are filtered to RBP genes and ranked by expression difference significance.

The TSES metric shows how high the reference transcript set ranks in the DTU result. Since this transcript set is derived from a specific RBP KO/KD experiment, while the DTU result comes from a new dataset comparing experimental and control conditions, TSES measures the similarity between the transcript changes caused by an RBP KO/KD and those observed in the new experiment. Therefore, a higher TSES score means that the current experimental condition is more similar to the knockout or knockdown of that RBP, indicating that the RBP shows a greater change in activity between the two conditions. In an extreme case, when the experimental condition itself is an RBP KO/KD, the TSES score of that RBP is expected to be among the highest across all RBPs.

RBP recovery in this downstream analysis is evaluated using this extreme case, in which one RBP is knocked out or knocked down in each dataset. Using the RBP KO/KD RNA‐seq database, which contains 11 RBP knockout and 63 RBP knockdown experiments, TSES scores are calculated for all RBPs, and the perturbed RBP is ranked in each dataset. In the 74 ENCODE RBP KO/KD RNA‐seq datasets, the recommended DTU + TSES strategy ranks the perturbed RBP first in all datasets. Within the present reference‐set and scoring framework, the recommended DTU + TSES strategy recovers the perturbed RBP more consistently than the evaluated DTE + TSES and gene‐level DGE strategies.

The DTE + TSES strategies rank the perturbed RBP first in 68/74 datasets for edgeR, 68/74 for limma, 40/74 for DESeq2, and 66/74 for edgeR‐Scaled. DGE methods rank the perturbed RBP within the top three in most cases, including 62/74 for edgeR, 59/74 for limma, and 57/74 for DESeq2 (Figure 7B; Table S7).

The same comparison is applied to four independent RBP knockout datasets from the GEO database, including MATR3 [23], RBM5 [24], PCBP1 [25], and TRA2A [26]. The recommended DTU + TSES again ranks the knockout RBP within the top three in all four datasets. DTE + TSES often prioritizes the true RBP but varies by method and dataset, especially in the PCBP1 and TRA2A cases. DGE methods can place the true RBP much lower in some cases, especially PCBP1 (Figure 7C; Table S7).

Together, these results show that, within the present reference set and scoring framework, the recommended DTU + TSES strategy recovers the perturbed RBPs more consistently than the evaluated DTE + TSES and gene‐level DGE strategies.

## 3 Discussion

This study presents a framework for systematic evaluation of DTU methods. Unlike previous benchmarking studies that rely on simulated datasets, the framework constructs reference transcript sets from real biological data and uses a DTU‐specific evaluation metric. This design reflects biological conditions more directly and provides evaluation with high unbiasedness, stability, and effectiveness. The framework also provides guidance for selecting suitable DTU methods across data types. In addition, the evaluation results and components of the framework are further combined for RBP activity prediction at the transcript level. In the tested datasets and within the present reference‐set and scoring framework, the recommended DTU + TSES strategy recovers perturbed RBPs more consistently than the evaluated DTE + TSES and gene‐level DGE strategies.

The evaluation results show that different DTU methods perform best on different data types. In bulk RNA‐seq data, satuRn achieves the highest accuracy. Its advantage comes from the quasi‐binomial generalized linear model, which directly models transcript proportions and accounts for dependencies among transcripts of the same gene. By applying empirical Bayes shrinkage to estimate overdispersion, satuRn provides reliable variance estimation even in small‐sample settings, thus combining high accuracy with computational efficiency in bulk data analysis [9]. In contrast, DRIMSeq performs better in long‐read single‐cell RNA‐seq and long‐read spatial RNA‐seq datasets. Its Dirichlet‐multinomial model effectively captures overdispersion and compositional effects in transcript proportions, making it suitable for sparse and highly variable data [7]. Since long‐read sequencing provides more accurate transcript quantification, DRIMSeq's assumptions more closely match the true data characteristics, improving both sensitivity and reliability. DTUrtle, which extends the DRIMSeq framework, applies stricter transcript filtering and integrates stageR for multiple testing correction, further improving precision in large datasets [8]. Therefore, within the evaluated datasets, DRIMSeq and DTUrtle show stronger performance in long‐read single‐cell RNA‐seq and long‐read spatial RNA‐seq analyses.

These rankings should be interpreted in relation to false‐discovery control and in the context of the data types and original design scope of each method. Figure S1C shows no significant correlation between TSES and empirical FDR across methods, suggesting that transcript‐ranking performance and false‐discovery control represent different dimensions of method performance. Although nine of the ten evaluated methods have a mean empirical FDR below 5%, dataset‐level median empirical FDR values nevertheless vary markedly across data types (Figure S1A,B). After excluding RATs, the dataset‐level median values range from 0% to 67.48% across the short‐read RNA‐seq datasets, substantially higher than the corresponding range of 0% to 4.88% across the long‐read datasets (Figure S1A). This difference may partly reflect read‐to‐transcript ambiguity caused by transcript overlap in short‐read RNA‐seq data [12]. Accordingly, FDR control remains challenging for DTU methods applied to short‐read RNA‐seq data [2, 12]. Notably, for satuRn, SPIT, and SUPPA2, empirical FDR is non‐estimable in some short‐read bulk RNA‐seq datasets because no transcript meets the significance threshold in the corresponding original analysis. Nevertheless, their high TSES rankings show that reference transcripts are enriched near the top of their DTU results. Moreover, most methods evaluated in long‐read bulk, single‐cell, and spatial transcriptomics are adapted from methods developed for short‐read RNA‐seq or from general statistical models rather than being specifically developed for these technologies, and there is no clearly leading DTU method for long‐read RNA‐seq [13]. Therefore, the results for these datasets should be interpreted as empirical benchmarking evidence rather than as the expected performance of methods specifically optimized for these technologies.

Because sufficiently large real datasets with independently validated transcript‐level changes remain limited, the framework uses the overlap between DTU‐method consensus and RBP‐related regulatory evidence as the reference transcript set, enabling evaluation without requiring experimentally established transcript‐level ground truth for each condition comparison. However, the consensus component of this reference set may favor methods whose results resemble those of the majority, whereas a biologically valid method that identifies distinct transcript‐usage changes may receive a lower TSES. The LOMO analysis reduces direct self‐contribution by excluding the method under evaluation from construction of its own reference set, and the similar TSES distributions and method rankings under LOMO indicate that the main conclusions are not primarily driven by this direct self‐contribution. Nevertheless, the LOMO reference set still incorporates consensus among the remaining nine methods, and residual consensus dependence therefore remains. Thus, the same reference‐set design that enables systematic DTU evaluation on real datasets without requiring independently validated transcript‐level ground truth also leaves the evaluation partly dependent on consensus among the evaluated methods.

Several points should be considered when interpreting the small‐sample analysis shown in Figures S22 and S23. First, in the rank stability analysis, long‐read spatial RNA‐seq data show weaker statistical support than other data types (Figure S24). This weaker stability is likely related to the high sparsity and high noise of long‐read spatial RNA‐seq data, because subsampling can strongly change the set of testable transcripts and the estimated transcript expression proportions. Second, for short‐read bulk RNA‐seq and long‐read bulk RNA‐seq data, many original contrasts already contain fewer than 3 or 5 samples per condition. Therefore, for these data types, the analysis mainly reflects repeated random subsampling of datasets that already contain only a limited number of samples. Nevertheless, this analysis remains informative because many biological experiments include equal numbers of samples in the experimental and control groups, and it assesses method performance under balanced group designs in bulk datasets. Third, although satuRn performs well in the small‐sample analyses of long‐read single‐cell RNA‐seq and long‐read spatial RNA‐seq data, single‐cell and spatial studies usually contain many more cells or spots than the subsampled setting tested here, and sparsity and noise can make rankings less stable when the available data are strongly reduced. Therefore, method recommendations remain based mainly on the original evaluation in Figure 5 and the LOMO evaluation in Figure S6.

Runtime is also compared across DTU methods. Among these methods, DEXSeq is the most time‐consuming and highly affected by sample size, but its runtime is comparable to others on small datasets. limma‐diffSplice, edgeR‐diffSplice, and satuRn show shorter runtimes overall (Figure S25).

The general applicability of the framework depends on the amount, diversity, and quality of real biological data. The RBP KO/KD datasets used in this study are mainly from the ENCODE project [27, 28, 29, 30, 31, 32], which follows strict standards in experimental design, library construction, sequencing, and data processing [33]. However, the number of publicly available RBP KO/KD RNA‐seq datasets is still limited [34]. Some RBPs have essential housekeeping roles, and their complete knockout causes cell death, making experimental data difficult to obtain. In addition, RBP knockout experiments are technically challenging and expensive. Apart from large projects like ENCODE, most studies focus on specific biological questions and therefore have limited scale. For example, using RNA‐seq data from RBP knockout experiments, Islam et al. report that the loss of MATR3 increases the expression of interferon‐stimulated genes and activates the cGAS‐STING pathway [23]. Zhang et al. show that RBM5 acts as an upstream regulator of HOXA9 and controls the development of acute myeloid leukemia [24]. Karam et al. find that PCBP1 regulates transcription through interaction with DHX9 [25], while Lee et al. reveal that TRA2A cooperates with its paralog TRA2B in splicing regulation [26]. Moreover, data produced by different laboratories are often scattered across databases such as GEO and the Sequence Read Archive (SRA), with inconsistent pipelines and incomplete metadata. These issues make data integration and reuse more difficult. At present, no long‐read RNA‐seq database for RBP KO/KD experiments exists, which limits the ability to evaluate DTU methods at the full‐length transcript level. Therefore, the lack of RBP KO/KD data remains one of the main barriers to the generalization of the evaluation framework.

The primary purpose of the RBP activity analysis is to demonstrate that the proposed framework can be extended beyond DTU method evaluation to downstream analyses. Specifically, the evaluation results and components of the framework can be further used and combined to address other biological questions. Here, the recommended DTU method, RBP‐specific overlapped transcript sets, and TSES are combined to predict RBP activity at the transcript level, while DTE + TSES and gene‐level DGE strategies are included for comparison. These comparisons should nevertheless be interpreted within the scope of the evaluated strategies. Using the same RBP‐specific reference transcript sets and TSES calculation makes it possible to examine the effect of replacing DTU‐derived rankings with DTE‐derived rankings, but it also means that the DTE + TSES strategies are not fully independent comparators. Moreover, because the reference transcript sets partly incorporate DTU‐method consensus, some favorability toward DTU‐derived rankings cannot be excluded. At present, the scope of this application is also limited by the availability of RBP KO/KD data. In addition, although the current analysis focuses on splicing‐related RBPs, the framework may be extended to other RBP types as suitable perturbation datasets become available. Despite these limitations, the results support the value of extending the framework beyond DTU method evaluation to downstream biological analyses.

## 4 Materials and Methods

### 4.1 Dataset Collection

We collect multiple publicly available transcript expression datasets, together with related information needed to construct the reference transcript sets. This includes RBP‐RNA interaction data, RBP annotation data, and genome reference sequences.

The selection of RNA‐seq datasets follows two main criteria:

1. Each data type should include multiple tissues or cell lines from the same experimental source, ensuring that metric stability is not affected by inconsistent experimental designs or processing pipelines.
2. Long‐read single‐cell RNA‐seq and long‐read spatial RNA‐seq datasets should include cell‐type or spatial‐domain annotations, allowing the generation of transcript expression data for different cell types or regions.

Following these criteria, we collect four types of RNA‐seq datasets, including RBP KO/KD short‐read bulk RNA‐seq, long‐read bulk RNA‐seq, long‐read single‐cell RNA‐seq, and long‐read spatial RNA‐seq datasets, as summarized in Table 1.

These collected RNA‐seq datasets are used at the transcript‐count level. The transcript quantification sources are as follows: (1) short‐read bulk RNA‐seq data: RSEM for the main analysis, with Salmon and Kallisto as alternatives; (2) long‐read bulk RNA‐seq data: TALON for the main analysis, with Bambu and IsoQuant as alternatives; (3) long‐read single‐cell RNA‐seq data: the FLAMES pipeline; and (4) long‐read spatial RNA‐seq data: the SiT / ScNaUmi‐seq‐derived Nanopore isoform unique molecular identifier (UMI) pipeline.

In addition, to construct the reference transcript sets, we also collected RBP‐RNA interaction data for 221 human RBPs from the ENCODE project and 46 mouse RBPs from POSTAR3. RBP annotation information is obtained from POSTAR3, and genome reference sequences are derived from GENCODE v29 (human) and GENCODE vM21 (mouse). Detailed information for all datasets is summarized in Table 2. ENCODE accession numbers for the short‐read bulk RNA‐seq and long‐read bulk RNA‐seq datasets are provided in Table S8. The number of samples in each dataset is summarized in Table S9.

**TABLE 2:** Details of Interaction, RBP and Sequence Information.

Resource	Refs.	Detail
**RBP binding signal**		
ENCODE	[28, 29]	RNA binding signal bound by 221 RBPs in human
**RBP‐RNA interaction**		
ENCODE	[28, 29]	RNA sequence information bound by 221 RBPs in human
POSTAR3	[38]	RNA sequence information bound by 46 RBPs in mouse
**RBP information**		
POSTAR3	[38]	356 RBPs information, including 111 alternative splicing‐related RBPs
**Genome Reference Sequence**		
GENCODE	[39]	GENCODE v29 for human and vM21 for mouse

### 4.2 DTU Methods Collection

The DTU methods evaluated in this study are selected according to three criteria:

1. The method takes transcript expression and annotation information from two biological conditions as input.
2. The method focuses on the transcript level rather than the gene level.
3. The method is easy to use and publicly available.

Following these criteria, the framework evaluates ten representative DTU tools: DEXSeq [6], DRIMSeq [7], DTUrtle [8], edgeR‐diffSplice [19], limma‐diffSplice [20], NBSplice [40], RATs [41], satuRn [9], SPIT [11], and SUPPA2 [10]. These tools cover a wide range of statistical models and analytical strategies, including generalized linear models, Dirichlet‐multinomial models, and non‐parametric tests. A summary of each method is provided in Table 3. For fair comparison, within each quantification setting, all DTU methods use the same transcript count matrix, transcript‐to‐gene annotation, sample labels, expression‐filtering rule, and downstream analysis workflow.

**TABLE 3:** Details of differential transcript usage (DTU) methods.

Method	Software	Model	Features
DEXSeq	R	Negative binomial GLM	Models exon–condition interaction; controls biological variance; suitable for complex designs but slow for large datasets.
DRIMSeq	R	Dirichlet‐Multinomial	Models multivariate transcript proportions per gene; tests for changes in transcript ratios between conditions; uses empirical Bayes for stable dispersion estimation.
DTUrtle	R	Dirichlet‐Multinomial	Extends DRIMSeq; adds adaptive filtering, visualization, and single‐cell support; employs two‐stage FDR control (stageR) for robust detection in bulk and scRNA‐seq data.
edgeR‐diffSplice	R	Negative Binomial GLM	Tests exon‐ or transcript‐level log‐fold changes vs gene‐level change; identifies differential usage across transcript features.
limma‐diffSplice	R	Linear Models	Uses moderated t‐tests to compare log‐fold changes among exons or transcripts within genes; identifies differential usage across transcript features.
NBSplice	R	Negative binomial GLM	Fits gene‐level GLM on transcript relative expression; tests transcript‐condition interaction via likelihood ratio or F‐test.
RATs	R	G‐test (non‐parametric)	Non‐parametric, count‐based approach; tests independence of transcript and condition; employs bootstrap resampling to assess DTU reliability.
satuRn	R	Quasi‐binomial GLM	Fast, memory‐efficient, and scalable; models relative transcript usage; uses an empirical null distribution for accurate FDR estimation; applicable to bulk and single‐cell RNA‐seq.
SPIT	Python	KDE + Mann‐Whitney U test (non‐parametric)	Identifies sample subgroups with distinct transcript usage via KDE; uses SPIT‐Test for robust FDR control; suitable for heterogeneous populations.
SUPPA2	Python	Empirical distribution (non‐parametric)	Estimates ΔPSI directly from transcript abundances; assesses significance based on replicate variability and expression‐dependent uncertainty; supports multi‐condition clustering and biological variability modeling.

Abbreviations: GLM, Generalized Linear Model; KDE, Kernel Density Estimation; DTU, Differential Transcript Usage; ΔPSI, Difference of Percent Spliced‐In.

### 4.3 Data Preprocessing

#### 4.3.1 RBP Filtering

The POSTAR3 database provides information for 356 RBPs. From this list, we select 111 RBPs that are known to be related to splicing regulation or spliceosome formation. These RBPs are referred to as alternative splicing‐related RBPs (AS‐related RBPs) in this study.

#### 4.3.2 RBP KO/KD RNA‐seq Dataset Filtering

The RBP KO/KD short‐read bulk RNA‐seq collection comprises 376 RBP KO/KD experiments in HepG2 and K562 cell lines. We retain 74 datasets corresponding to AS‐related RBPs, including 5 CRISPR knockouts in HepG2 (CRISPR_HepG2), 6 CRISPR knockouts in K562 (CRISPR_K562), 32 shRNA knockdowns in HepG2 (shRNA_HepG2), and 31 shRNA knockdowns in K562 (shRNA_K562).

#### 4.3.3 Transcript Expression Preprocessing

To reduce false positives in DTU detection and remove lowly expressed transcripts, we apply an expression filter to each dataset before analysis.

Only transcripts that meet both the minimum expression threshold and the minimum number of samples are retained:

- RBP KO/KD short‐read bulk RNA‐seq data: keep transcripts with expression >5 in at least 2 samples.
- Long‐read bulk RNA‐seq data: keep transcripts with expression >5 in at least 3 samples.
- Long‐read single‐cell RNA‐seq data: keep transcripts with expression >3 in at least 3 cells.
- Long‐read spatial RNA‐seq data: keep transcripts with expression >2 in at least 2 spots.

For DTU evaluation, condition pairs are constructed within each dataset. Short‐read bulk RNA‐seq datasets use RBP KO/KD versus matched control comparisons. Long‐read bulk RNA‐seq datasets use pairwise comparisons among cell lines or tissues, long‐read single‐cell RNA‐seq datasets use pairwise comparisons among annotated cell groups, and long‐read spatial RNA‐seq datasets use pairwise comparisons among spatial domains. The analysis units are individual RBP KO/KD or matched‐control replicates for short‐read bulk RNA‐seq, individual tissue samples for long‐read bulk RNA‐seq, individual cells for long‐read single‐cell RNA‐seq, and individual spots for long‐read spatial RNA‐seq. For each dataset and condition pair, one transcript count matrix is first generated, and the data‐type‐specific expression filter is applied once before DTU analysis. The same filtered count matrix, condition labels, and transcript‐to‐gene annotation are then used as input for all ten DTU methods.

#### 4.3.4 ENCODE RBP Binding Signal Data Preprocessing

ENCODE eCLIP RBP binding signal data are collected as independent evidence for validating reference transcript sets. For each RBP KO/KD dataset, matched eCLIP peak files from the same RBP and cell line are used when available. Peak signal values are mapped to GENCODE v29 transcript annotations, and transcript‐level binding signals are calculated across available replicates. These signals are used to compare transcripts within and outside the regulated, common, and overlapped transcript sets.

#### 4.3.5 RBP‐RNA Interaction Data Preprocessing

For human RBP‐RNA interaction data, we extract information from POSTAR3 for 221 RBPs originating from the ENCODE project. ENCODE uses standardized experimental procedures and data processing pipelines, reducing technical bias across RBPs. Moreover, its use of the same genome reference and cell lines ensures compatibility with the RBP KO/KD RNA‐seq datasets. For mouse RBP‐RNA interaction data, POSTAR3 integrates results from various studies. To ensure reliability, we retain only interactions detected in at least two independent samples.

### 4.4 RBP‐Transcript Interaction Network Construction

We build RBP‐transcript interaction networks by mapping RBP‐RNA interaction sequences to their corresponding transcripts. Specifically, RBP‐RNA interaction data from POSTAR3 are aligned to the GENCODE reference genome (v29 for human, vM21 for mouse) using the bedtoolsr R package (“bt.intersect” function). This mapping identifies which transcripts correspond to the interacting RNA regions. By combining the RBP–RNA and RNA–transcript information, we generate an RBP‐transcript interaction network that links each RBP to its target transcripts.

### 4.5 Reference Transcript Set Construction

In this evaluation framework, we define three types of reference transcript sets:

- Common transcript set: includes transcripts that are identified as differential by at least half of the DTU methods (i.e., detected by at least five methods).
- Regulated transcript set: includes transcripts known to be regulated by RNA‐binding proteins (RBPs) according to RBP–transcript interactions in RBP KO/KD datasets or predicted by the trained model in non‐KO/KD RNA‐seq datasets.
- Overlapped transcript set: represents the intersection of the common and regulated transcript sets. This set is used as the main reference in all subsequent evaluations because it combines biological evidence with methodological consensus.

### 4.6 Identification of Regulated Transcripts

For RBP KO/KD RNA‐seq datasets, we directly extract all transcripts that interact with the knocked‐out RBP from the RBP‐transcript interaction network to form the regulated transcript set. For non‐KO/KD RNA‐seq datasets (such as long‐read bulk RNA‐seq, long‐read single‐cell RNA‐seq, and long‐read spatial RNA‐seq datasets collected in this study), we integrate multiple RBP KO/KD datasets into machine learning models to predict regulated transcripts. Specifically, for each RBP KO/KD dataset, we calculate two types of changes between conditions (e.g., control vs. treatment): the fold change in RBP gene expression and the fold change in transcript expression proportion. Then we combine these two types of fold change values from multiple RBP KO/KD datasets to generate the training data and select a machine learning model to train the prediction model. The detailed procedure is as follows:

#### 4.6.1 RBP Gene Expression Fold Change

For a given alternative splicing‐related RBP gene *g*, let its expression value under condition *A* (e.g., KO/KD) in sample or cell *a* be $x_{g,a}^{A}$, and under condition *B* (e.g., control) in sample or cell *b* be $x_{g,b}^{B}$.

The RBP gene expression fold change between the two conditions is defined as:

$$FC_{g}^{RBP}=abs\left(log_{2}\left(\frac{\underset{a\in A}{mean}\left(x_{g,a}^{A}\right)+1}{\underset{b\in B}{mean}\left(x_{g,b}^{B}\right)+1}\right)\right)$$

Further, normalize all fold changes by max fold change to maintain comparability across genes:

$$normFC_{g}^{RBP}=\frac{FC_{g}^{RBP}}{\underset{g}{max}\left(FC_{g}^{RBP}\right)}$$

#### 4.6.2 Transcript Expression Proportion Fold Change

Let gene *g* contain *N_g_* transcripts and denote its *k*‐th transcript as *u*_*g*,*k*_. For transcript *u*_*g*,*k*_, let its expression value under condition *A* in sample or cell *a* be $y_{u_{g,k},a}^{A}$ and under condition *B* in sample or cell *b* be $y_{u_{g,k},b}^{B}$.

The expression proportion of transcript within its parent gene is defined as:

$$y_{u_{g,k},a}^{\prime A}=\frac{y_{u_{g,k},a}^{A}}{\sum_{k}y_{u_{g,k},a}^{A}},\;y_{u_{g,k},b}^{\prime B}=\frac{y_{u_{g,k},b}^{B}}{\sum_{k}y_{u_{g,k},b}^{B}}$$

The fold change in transcript expression proportion between the two conditions is then calculated as:

$$FC_{u}^{isoform}=abs\left(log_{2}\left(\frac{\underset{a\in A}{mean}\left(y_{u_{g,k},a}^{\prime A}\right)+0.01}{\underset{b\in B}{mean}\left(y_{u_{g,k},b}^{\prime B}\right)+0.01}\right)\right)$$

Further, normalize all fold changes by the maximum fold change to maintain comparability across transcripts:

$$normFC_{u}^{isoform}=\frac{FC_{u}^{isoform}}{\underset{u}{max}\left(FC_{u}^{isoform}\right)}$$

#### 4.6.3 Training Data Generation

Let each RBP‐transcript regulatory relationship in the RBP‐transcript interaction network be represented as a pair (*g,u*), where *g* denotes an RBP gene, and *u* denotes its potentially regulated transcript. The combined regulatory change associated with this pair is characterized by a 2D feature vector that integrates both the RBP gene expression change and the transcript proportion change:

$$w_{g,u}=\left(normFC_{g}^{RBP},normFC_{u}^{isoform}\right)$$

To enhance the generalizability of the prediction model, we aggregated training data from multiple individual RBP KO/KD experiments. Formally, if the number of RBP KO/KD datasets is *M_KO_*, the combined dataset is defined as:

$$w_{g,u}^{merge}=\left(\begin{array}{c} w_{g,u}^{1}\\ w_{g,u}^{1}\\ \text{…}\\ w_{g,u}^{M_{KO}} \end{array}\right)$$

The merged dataset $w_{g,u}^{merge}$ thus captures regulatory variation across multiple RBP KO/KD experiments. All RBP‐transcript pairs directly regulated by a given RBP are labeled as positive labels. These positive pairs, together with unlabeled background pairs, are used to train the predictive model for identifying regulated transcripts in non‐KO/KD datasets.

#### 4.6.4 Prediction Model

A generalized linear model (GLM) is trained using the *caret* R package with default settings. To evaluate model sensitivity, we also train a linear discriminant analysis (LDA) model using the same training data and parameters. After training, the model can predict regulated transcripts in non‐KO/KD datasets.

### 4.7 Computation of Transcript Set Enrichment Score (TSES)

The transcript set enrichment score (TSES) quantifies whether a reference transcript set *R* is preferentially ranked at the top of a transcript list ordered by statistical significance obtained from a DTU method.

The computation procedure consists of four steps:

#### 4.7.1 Compute Transcript‐Level Statistics

Let all transcripts be represented by the set *U* = {*u*_1_,..., *u_N_*}. For each transcript *u* ∈ *U*, the P‐value from a DTU method as *pval_u_* is transformed into a statistical significance score:

$$Sig_{u}=-log_{10}\left(Pval_{u}\right)$$

#### 4.7.2 Rank All Transcripts

All transcripts are sorted in descending order based on *Sig_u_*, producing a ranked sequence:

$$S=(u_{\left(1\right)},u_{\left(2\right)},\text{…},u_{\left(N\right)})$$

such that,

$$Sig_{u_{\left(1\right)}}\geq Sig_{u_{\left(2\right)}}\geq \text{…}\geq Sig_{u_{\left(N\right)}}$$

#### 4.7.3 Calculate Enrichment Score

Let the reference transcript set be *R*⊂*U*, and denote its size as |*R*| = *N_R_*. For each position *i* in the ranked list *S*, the cumulative significance for hits (transcripts in *R*) is:

CumSigR,ihit=∑u(j)∈Rj≤iSigu(j)αNR

and the cumulative significance for misses (transcripts not in *R*) is:

CumSigR,imiss=∑u(j)∉Rj≤i1N−NR

The enrichment score (ES) is the maximum deviation between the two cumulative significances:

$$ES=ES\left(R,S\right)=\underset{1\leq i\leq N}{max(}\left|CumSig_{R,i}^{hit}-CumSig_{R,i}^{miss}\right|)$$

#### 4.7.4 Normalize and Statistical Test

To estimate statistical significance, the transcript labels are randomly permuted *T* times (typically *T* = 1000) to generate a null distribution of enrichment scores $ES_{t}^{null}$ for *t* = 1,2,…,*T*.

The transcript set enrichment score (TSES) is the normalized enrichment score (NES) comparing the observed *ES* to the null distribution:

$$TSES=TSES\left(R,S\right)=NES\left(R,S\right)=\frac{ES}{\underset{t}{mean}\left(ES_{t}^{null}\right)}$$

The P‐value of the TSES is defined as the fraction of permutations where the randomized score exceeds the real score:

$$P-value_{ISES}=\frac{\sum_{t}I(ES_{t}^{null}\geq ES)+1}{n+1}$$

where *I*(·) is an indicator function defined as:

$$I\left(condition\right)=\left\{\begin{array}{cc} 1, & ifconditionisTrue\\ 0, & otherwise \end{array}\right.$$

This score quantifies whether reference transcripts tend to appear at the top of a DTU method's ranked list, providing a biologically meaningful measure of method accuracy.

### 4.8 Other Accuracy Evaluation Metrics

To compare TSES with commonly used measures, we computed three additional accuracy indices:

- Jaccard coefficient, quantifying the overlap between predicted transcripts and reference transcripts;
- AUPRC, Area Under the Precision–Recall Curve;
- AUROC, Area Under the Receiver Operating Characteristic Curve.

These metrics are computed using standard R packages: jaccard() from *jaccard*, roc() from *pROC*, and pr.curve() from *PRROC*.

### 4.9 Quantifying Unbiasedness, Stability, and Effectiveness

We quantify three key properties of the proposed metric to ensure the reliability of the evaluation framework.

#### 4.9.1 Unbiasedness

Unbiasedness measures whether the accuracy score of a DTU method is affected by the number of detected transcripts. Ideally, the correlation between the score and the number of predictions should be close to zero. A high correlation indicates low unbiasedness, while a low correlation means high unbiasedness.

Let the accuracy score (e.g., TSES) of the *l*‐th DTU method (among all *L* methods) for data type *i* (e.g., long‐read bulk RNA‐seq, long‐read single‐cell RNA‐seq, or long‐read spatial RNA‐seq) on dataset *j* (e.g., the CRISPR_HepG2 dataset or the CBS1 dataset) and comparison group *k* (e.g., a KO/KD group vs. control) be denoted as *Acc*_*i*,*j*, *k*, *l*_. Let *N*_*i*,*j*, *k*, *l*_denote the number of significant transcripts predicted by that method. The correlation between *accInd*_*i*,*j*, *k*, *l*_and *N*_*i*,*j*, *k*, *l*_within each comparison group is used to quantify the unbiasedness:

$$Unbiasedness_{i,j,k}=1-\mathrm{abs}\left(\mathrm{spearman}\left(\left(\begin{array}{c} Acc_{i,j,k,1}\\ Acc_{i,j,k,2}\\ \vdots \\ Acc_{i,j,k,L} \end{array}\right),\left(\begin{array}{c} N_{i,j,k,1}\\ N_{i,j,k,2}\\ \vdots \\ N_{i,j,k,L} \end{array}\right)\right)\right)$$

The overall unbiasedness is defined as the median unbiasedness across all data types, datasets, and comparison groups:

$$Unbiasedness=\underset{i,j,k}{\mathrm{median}}\left(Unbiasedness_{i,j,k}\right)$$

#### 4.9.2 Stability

Stability measures the consistency of the relative rankings of DTU methods across datasets/data subtypes. A stable framework should give similar scores for a method when applied to different datasets of the same data type.

Let the accuracy scores of the *l*‐th DTU method in the *i*‐th data type and *j*‐th dataset for the *k_1_*‐th and *k_2_*‐th comparison groups (where *k*_1_ ≠ *k*_2_) be $Acc_{i,j,k_{1},l}$ and $Acc_{i,j,k_{2},l}$.

The pairwise correlation between $Acc_{i,j,k_{1},l}$ and $Acc_{i,j,k_{2},l}$ reflects stability for that method.

$$Stability_{i,j,k_{1},k_{2}}=\mathrm{spearman}\left(\left(\begin{array}{c} Acc_{i,j,k_{1},1}\\ Acc_{i,j,k_{1},2}\\ \vdots \\ Acc_{i,j,k_{1},L} \end{array}\right),\left(\begin{array}{c} Acc_{i,j,k_{2},1}\\ Acc_{i,j,k_{2},2}\\ \vdots \\ Acc_{i,j,k_{2},L} \end{array}\right)\right)$$

The overall stability is the median of all pairwise correlations across all datasets, data types, and methods:

$$Stability=\underset{i,j,k_{1}\neq k_{2}}{\mathrm{median}}\;\left(Stability_{i,j,k_{1},k_{2}}\right)$$

#### 4.9.3 Effectiveness

Effectiveness reflects whether the evaluation metric truly quantifies the accuracy of DTU methods. Using a permutation test, accuracy scores are compared with those obtained after randomly shuffling the labels of the reference transcript set. If the evaluation framework is effective, the accuracy scores are significantly higher than the scores from the null distribution.

Let the accuracy score of the *l*‐th DTU method for data type *i*, dataset *j*, and transcript group *k* be *Acc*_*i*,*j*, *k*, *l*_. After performing *T* = 1000 random permutations of transcript labels, the accuracy score in the null distribution is denoted as $Acc_{i,j,k,l,t}^{null}$ for *t* = 1,2,…,*T*.

The effectiveness is then calculated as:

$$Effectiveness_{i,j,k,l}=1-\frac{\sum_{t}I(accInd_{i,j,k,l,t}^{null}\geq accInd_{i,j,k,l})+1}{T+1}$$

where *I*(·) is the indicator function:

$$I\left(condition\right)=\left\{\begin{array}{cc} 1, & ifconditionisTrue\\ 0, & otherwise \end{array}\right.$$

The overall effectiveness is the median of all individual effectiveness scores across data types, datasets, and methods:

$$Effectiveness=\underset{i,j,k,l}{\mathrm{median}}\;\left(Effectiveness_{i,j,k,l}\right)$$

This comprehensive quantification ensures that the proposed framework provides unbiased, stable, and effective assessments of various DTU methods under different data types.

### 4.10 RBP Activity Prediction and Comparator Strategies

RBP activity is predicted by combining the DTU method recommended for each data type with TSES. satuRn is used for bulk RNA‐seq, whereas DRIMSeq is designated for long‐read single‐cell and spatial RNA‐seq. Because the ENCODE and GEO perturbation benchmarks analyzed here consist of bulk RNA‐seq data, satuRn is used for the reported recommended DTU + TSES results. For each candidate RBP, its RBP‐specific overlapped transcript set is used as the reference set, and TSES is calculated from transcripts ranked by DTU significance. Candidate RBPs are subsequently ranked by their TSES values. The RBP activity prediction procedure consists of three steps:

#### 4.10.1 Select Recommended DTU Method

Based on our evaluation, satuRn is recommended for bulk RNA‐seq data, whereas DRIMSeq is recommended for long‐read single‐cell RNA‐seq and long‐read spatial RNA‐seq data. Because the data used in the RBP activity prediction analysis are bulk RNA‐seq, satuRn is selected for subsequent analysis.

#### 4.10.2 Calculate Transcript Set Enrichment Score for Each RBP

Given transcript expression data of the experiment and control group, we obtain the significance scores $S_{g}^{satuRn}$ of each RBP *g* generated by satuRn. We collect overlapped transcript set $R_{g}^{overlapped}$ for each RBP *g* included in the RBP KO/KD dataset (with a total of *M* RBPs) as the reference transcript set; we then calculate the transcript set enrichment score (TSES) for that RBP:

$$TSES_{g}=TSES\;\left(R_{g}^{overlapped},S_{g}^{satuRn}\right)$$

The *TSES_g_* value of RBP *g* measures how similar the transcript changes in the current experiment are to those caused by the KO/KD of this RBP. In other words, it reflects how strongly the transcripts regulated by RBP *g* are enriched among the top‐ranked transcripts in the *satuRn* results. A higher TSES score indicates that the experimental condition produces transcript usage patterns more similar to those from the RBP KO/KD, suggesting a stronger change in the activity of this RBP between the experimental and control groups.

#### 4.10.3 Rank Transcript Set Enrichment Score for All RBPs

Finally, we rank the TSES values of all RBPs and obtain the sequence:

$$\left(TSES_{g_{\left(1\right)}},TSES_{g_{\left(2\right)}},\text{…},TSES_{g_{\left(M\right)}}\right)$$

satisfied:

$$TSES_{g_{\left(1\right)}}\geq TSES_{g_{\left(2\right)}}\geq \text{…}\geq TSES_{g_{\left(M\right)}}$$

where*g*_(1)_represents the RBP predicted to show the most significant activity change.

Two comparator strategies are evaluated. First, differential transcript expression is analyzed using edgeR, limma, DESeq2, and edgeR‐Scaled. Transcripts are ranked according to their reported statistical significance, and the same RBP‐specific reference transcript sets and TSES calculation are applied to obtain the DTE + TSES rankings. Second, differential gene expression is analyzed using edgeR, limma, and DESeq2. The resulting gene‐level rankings are restricted to RBP genes, which are ranked according to differential‐expression significance without applying TSES. Prediction performance is measured by the rank assigned to the experimentally knocked‐out or knocked‐down RBP in each dataset.

### 4.11 Empirical FDR Estimation

Empirical FDR is evaluated using five random sample‐label permutations for each dataset contrast. Significance is defined at 0.05 using each method's adjusted‐significance output in both the original and permuted analyses. For SPIT and SUPPA2, for which the analyzed outputs do not contain an adjusted‐significance column, we adjust the nominal P‐values using the Benjamini‐Hochberg procedure. For each method and contrast, we calculate the uncapped empirical FDR estimate as the mean number of significant transcripts across the five sample‐label permutations divided by the number detected in the corresponding original analysis. Because the uncapped empirical FDR can exceed 100% when the number of significant transcripts in the original analysis is small, we define the capped empirical FDR as the smaller of 100% and the uncapped empirical FDR estimate. Throughout the manuscript, the capped empirical FDR values are referred to as ‘empirical FDR’ and are used in all statistical summaries and visualizations. When no transcript meets the adjusted‐significance threshold in the original analysis, we consider the empirical FDR non‐estimable and record it as missing. Both uncapped and capped empirical FDR estimates are reported in Table S2.

### 4.12 Leave‐One‐Method‐Out Analysis

In the leave‐one‐method‐out (LOMO) analysis, the method being evaluated is removed before construction of the common transcript set. A transcript is retained in the LOMO common transcript set when it is detected as differential in at least five of the remaining nine methods. The LOMO common transcript set is intersected with the regulated transcript set to generate the corresponding LOMO overlapped reference transcript set, and TSES is then recalculated for the excluded method.

## Author Contributions

C.Z. and R.Z. conceived the study. C.Z. conducted the experiments, performed the data analyses, and visualized the results. J.L., Q.Z., and H.Y. assisted in data processing and interpretation and provided technical support. A.Y. provided scientific advice and contributed to the biological interpretation of the results. C.Z. wrote the manuscript, which was revised by M.Z. and R.Z. All authors reviewed and approved the final manuscript.

## Funding

This work was supported by National Natural Science Foundation of China (Grant No. 82173046, 82472702), Shaanxi Province Funds for Distinguished Young Youths (Grant No. 2023‐JC‐JQ‐66), Shaanxi Province Key Industrial Innovation Chain Project (Grant No. 2024SF ZDCYL‐04‐04), the Fund of State Key Laboratory of Holistic Integrative Management of Gastrointestinal Cancers (Grants No. 2025GTKP005, CBSKL2022ZZ04, CBSKL2022KF06).

## Code Availability

All code used in this work is available on GitHub (https://github.com/chenxing‐zhang/DTU_evaluation_framework).

## Conflicts of Interest

The authors declare no conflicts of interest.

## Supporting information




**Supporting File 1**: advs77756‐sup‐0001‐SuppMat.pdf.

**Supporting File 2**: advs77756‐sup‐0002‐TableS1.xlsx.

**Supporting File 3**: advs77756‐sup‐0003‐TableS2.xlsx.

**Supporting File 4**: advs77756‐sup‐0004‐TableS3.xlsx.

**Supporting File 5**: advs77756‐sup‐0005‐TableS4.xlsx.

**Supporting File 6**: advs77756‐sup‐0006‐TableS5.xlsx.

**Supporting File 7**: advs77756‐sup‐0007‐TableS6.xlsx.

**Supporting File 8**: advs77756‐sup‐0008‐TableS7.xlsx.

**Supporting File 9**: advs77756‐sup‐0009‐TableS8.xlsx.

**Supporting File 10**: advs77756‐sup‐0010‐TableS9.xlsx.

## Data Availability

The data that support the findings of this study are openly available in Zenodo at https://doi.org/10.5281/zenodo.17698057.

## References

[1] C. Soneson , M. I. Love , and M. D. Robinson , “Differential Analyses for RNA‐seq: Transcript‐Level Estimates Improve Gene‐Level Inferences,” F1000Research 4 (2016): 1521. 10.12688/f1000research.7563.1 PMC4712774 26925227

[2] C. Soneson , K. L. Matthes , M. Nowicka , C. W. Law , and M. D. Robinson , “Isoform Prefiltering Improves Performance of Count‐Based Methods for Analysis of Differential Transcript Usage,” Genome Biology 17, no. 1 (2016): 12. 10.1186/s13059-015-0862-3 PMC4729156 26813113

[3] E. T. Wang , R. Sandberg , S. Luo , et al., “Alternative Isoform Regulation in Human Tissue Transcriptomes,” Nature 456, no. 7221 (2008): 470–476. 10.1038/nature07509 PMC2593745 18978772

[4] Q. Pan , O. Shai , L. J. Lee , B. J. Frey , and B. J. Blencowe , “Deep Surveying of Alternative Splicing Complexity in the Human Transcriptome by High‐Throughput Sequencing,” Nature Genetics 40, no. 12 (2008): 1413–1415. 10.1038/ng.259 18978789

[5] Y. Liu , M. Gonzàlez‐Porta , S. Santos , et al., “Impact of Alternative Splicing on the Human Proteome,” Cell Reports 20, no. 5 (2017): 1229–1241. 10.1016/j.celrep.2017.07.025 PMC5554779 28768205

[6] S. Anders , A. Reyes , and W. Huber , “Detecting Differential Usage of Exons from RNA‐seq Data,” Genome Research 22, no. 10 (2012): 2008–2017. 10.1101/gr.133744.111 PMC3460195 22722343

[7] M. Nowicka and M. D. Robinson , “DRIMSeq: A Dirichlet‐Multinomial Framework for Multivariate Count Outcomes in Genomics,” F1000Research 5 (2016): 1356. 10.12688/f1000research.8900.1 PMC5200948 28105305

[8] T. Tekath and M. Dugas , “Differential Transcript Usage Analysis of Bulk and Single‐Cell RNA‐seq Data with DTUrtle,” Bioinformatics 37, no. 21 (2021): 3781–3787. 10.1093/bioinformatics/btab629 PMC8570804 34469510

[9] J. Gilis , K. Vitting‐Seerup , K. Van Den Berge , and L. Clement , “satuRn: Scalable Analysis of Differential Transcript Usage for Bulk and Single‐Cell RNA‐Sequencing Applications,” F1000Research 10 (2022): 374. 10.12688/f1000research.51749.1 PMC9892655 36762203

[10] J. L. Trincado , J. C. Entizne , G. Hysenaj , et al., “SUPPA2: Fast, Accurate, and Uncertainty‐Aware Differential Splicing Analysis Across Multiple Conditions,” Genome Biology 19, no. 1 (2018): 40. 10.1186/s13059-018-1417-1 PMC5866513 29571299

[11] B. Erdogdu , A. Varabyou , S. C. Hicks , S. L. Salzberg , and M. Pertea , “Detecting Differential Transcript Usage in Complex Diseases with SPIT,” Cell Reports Methods 4, no. 3 (2024): 100736. 10.1016/j.crmeth.2024.100736 PMC10985272 38508189

[12] P. L. Baldoni , L. Chen , M. Li , Y. Chen , and G. K. Smyth , “Dividing Out Quantification Uncertainty Enables Assessment of Differential Transcript Usage with Limma and edgeR,” Nucleic Acids Research 53, no. 22 (2025): gkaf1305. 10.1093/nar/gkaf1305 PMC12684397 41359378

[13] X. Dong , M. R. M. Du , Q. Gouil , et al., “Benchmarking Long‐Read RNA‐Sequencing Analysis Tools Using In Silico Mixtures,” Nature Methods 20, no. 11 (2023): 1810–1821. 10.1038/s41592-023-02026-3 37783886

[14] G. A. Merino , A. Conesa , and E. A. Fernández , “A Benchmarking of Workflows for Detecting Differential Splicing and Differential Expression at Isoform Level in Human RNA‐seq Studies,” Briefings in Bioinformatics 20, no. 2 (2019): 471–481. 10.1093/bib/bbx122 29040385

[15] C. T. Lio , T. Düz , M. Hoffmann , et al., “Comprehensive Benchmark of Differential Transcript Usage Analysis for Bulk and Single‐Cell RNA Sequencing,” NAR Genomics and Bioinformatics 7, no. 3 (2025): lqaf117.

[16] X.‐D. Fu and M. Ares , “Context‐Dependent Control of Alternative Splicing by RNA‐Binding Proteins,” Nature Reviews Genetics 15, no. 10 (2014): 689–701. 10.1038/nrg3778 PMC4440546 25112293

[17] Y. Tao , Q. Zhang , H. Wang , X. Yang , and H. Mu , “Alternative Splicing and Related RNA Binding Proteins in Human Health and Disease,” Signal Transduction and Targeted Therapy 9, no. 1 (2024): 26. 10.1038/s41392-024-01734-2 PMC10835012 38302461

[18] A. Subramanian , P. Tamayo , V. K. Mootha , et al., “Gene Set Enrichment Analysis: A Knowledge‐Based Approach for Interpreting Genome‐Wide Expression Profiles,” Proceedings of the National Academy of Sciences 102 (2005): 15545. 10.1073/pnas.0506580102 PMC1239896 16199517

[19] Y. Chen , L. Chen , A. T. L. Lun , P. L. Baldoni , and G. K. Smyth , “edgeR v4: Powerful Differential Analysis of Sequencing Data with Expanded Functionality and Improved Support for Small Counts and Larger Datasets,” Nucleic Acids Research 53 (2025): gkaf018. 10.1093/nar/gkaf018 PMC11754124 39844453

[20] M. E. Ritchie , B. Phipson , D. Wu , et al., “limma Powers Differential Expression Analyses for RNA‐Sequencing and Microarray Studies,” Nucleic Acids Research 43, no. 7 (2015): 47. 10.1093/nar/gkv007 PMC4402510 25605792

[21] M. I. Love , W. Huber , and S. Anders , “Moderated Estimation of Fold Change and Dispersion for RNA‐seq Data with DESeq2,” Genome Biology 15, no. 12 (2014): 550. 10.1186/s13059-014-0550-8 PMC4302049 25516281

[22] P. L. Baldoni , Y. Chen , S. Hediyeh‐zadeh , et al., “Dividing Out Quantification Uncertainty Allows Efficient Assessment of Differential Transcript Expression with edgeR,” Nucleic Acids Research 52, no. 3 (2024): 13. 10.1093/nar/gkad1167 PMC10853777 38059347

[23] Z. Islam , A. Polash , M. Suzawa , et al., “MATRIN3 Deficiency Triggers Autoinflammation via cGAS‐STING Activation,” BioRxiv (2024): 2024.4.1.587645.

[24] M. Zhang , J. Hyle , X. Chen , et al., “RNA‐Binding Protein RBM5 Plays an Essential Role in Acute Myeloid Leukemia by Activating the Oncogenic Protein HOXA9,” Genome Biology 25, no. 1 (2024): 16. 10.1186/s13059-023-03149-8 PMC10785552 38216972

[25] J. A. Q. Karam , C. Fréreux , B. K. Mohanty , et al., “The RNA‐Binding Protein PCBP1 Modulates Transcription by Recruiting the G‐Quadruplex‐Specific Helicase DHX9,” Journal of Biological Chemistry 300, no. 11 (2024): 107830. 10.1016/j.jbc.2024.107830 PMC11538862 39342995

[26] A. R. Lee , A. Tangiyan , I. Singh , and P. S. Choi , “Incomplete Paralog Compensation Generates Selective Dependency on TRA2A in Cancer,” PLOS Genetics 21, no. 5 (2025): 1011685. 10.1371/journal.pgen.1011685 PMC12077678 40367120

[27] The ENCODE Project Consortium , “An Integrated Encyclopedia of DNA Elements in the Human Genome,” Nature 2012, 489, 7414 57. 10.1038/nature11247 PMC3439153 22955616

[28] E. L. Van Nostrand , P. Freese , G. A. Pratt , et al., “A Large‐Scale Binding and Functional Map of Human RNA‐Binding Proteins,” Nature 583, no. 7818 (2020): 711–719. 10.1038/s41586-020-2077-3 PMC7410833 32728246

[29] J. Zhang , D. Lee , V. Dhiman , et al., “An Integrative ENCODE Resource for Cancer Genomics,” Nature Communications 11, no. 1 (2020): 3696. 10.1038/s41467-020-14743-w PMC7391744 32728046

[30] J. Zhang , J. Liu , D. Lee , et al., “RADAR: Annotation and Prioritization of Variants in the Post‐Transcriptional Regulome of RNA‐Binding Proteins,” Genome Biology 21, no. 1 (2020): 151. 10.1186/s13059-020-01979-4 PMC7391703 32727537

[31] Y. Luo , B. C. Hitz , I. Gabdank , et al., “New Developments on the Encyclopedia of DNA Elements (ENCODE) Data Portal,” Nucleic Acids Research 48, no. D1 (2020): D882–D889. 10.1093/nar/gkz1062 PMC7061942 31713622

[32] M. S. Kagda , B. Lam , C. Litton , et al., “Data Navigation on the ENCODE Portal,” Nature Communications 16, no. 1 (2025): 9592. 10.1038/s41467-025-64343-9 PMC12575607 41168159

[33] B. C. Hitz , J.‐W. Lee , O. Jolanki , et al., “The ENCODE Uniform Analysis Pipelines,” BioRxiv (2023): 2023.04.04.535623.

[34] M. W. Hentze , A. Castello , T. Schwarzl , and T. Preiss , “A Brave New World of RNA‐Binding Proteins,” Nature Reviews Molecular Cell Biology 19, no. 5 (2018): 327–341. 10.1038/nrm.2017.130 29339797

[35] F. Reese , B. Williams , G. Balderrama‐Gutierrez , et al., “The ENCODE4 Long‐Read RNA‐seq Collection Reveals Distinct Classes of Transcript Structure Diversity,” *bioRxiv* (2023): 2023.5.15.540865.

[36] L. Tian , J. S. Jabbari , R. Thijssen , et al., “Comprehensive Characterization of Single‐Cell Full‐Length Isoforms in Human and Mouse with Long‐Read Sequencing,” Genome Biology 22, no. 1 (2021): 310. 10.1186/s13059-021-02525-6 PMC8582192 34763716

[37] K. Lebrigand , J. Bergenstråhle , K. Thrane , et al., “The Spatial Landscape of Gene Expression Isoforms in Tissue Sections,” Nucleic Acids Research 51, no. 8 (2023): 47. 10.1093/nar/gkad169 PMC10164556 36928528

[38] W. Zhao , S. Zhang , Y. Zhu , et al., “POSTAR3: An Updated Platform for Exploring Post‐Transcriptional Regulation Coordinated by RNA‐Binding Proteins,” Nucleic Acids Research 50, no. D1 (2022): D287–D294. 10.1093/nar/gkab702 PMC8728292 34403477

[39] J. M. Mudge , S. Carbonell‐Sala , M. Diekhans , et al., “GENCODE 2025: Reference Gene Annotation for Human and Mouse,” Nucleic Acids Research 53, no. D1 (2025): D966–D975. 10.1093/nar/gkae1078 PMC11701607 39565199

[40] G. A. Merino and E. A. Fernández , “Differential Splicing Analysis Based on Isoforms Expression with NBSplice,” Journal of Biomedical Informatics 103 (2020): 103378. 10.1016/j.jbi.2020.103378 31972288

[41] K. Froussios , K. Mourão , G. Simpson , G. Barton , and N. Schurch , “Relative Abundance of Transcripts (RATs): Identifying Differential Isoform Abundance from RNA‐seq,” F1000Research 8 (2019): 213. 10.12688/f1000research.17916.1 PMC6426083 30906538

