# Supplementary material for: A Real‐Data‐Driven Framework for Evaluating Differential Transcript Usage Methods Across Long‐Read Bulk, Single‐Cell, and Spatial Transcriptomics
Source: Adv Sci (Weinh). 2026 Sep 17:e77756. Online ahead of print. doi: 10.1002/advs.77756 (PMC13586480; doi:10.1002/advs.77756)
Supplement: Supplementary file 1 [file ADVS-9999-e77756-s009.pdf]

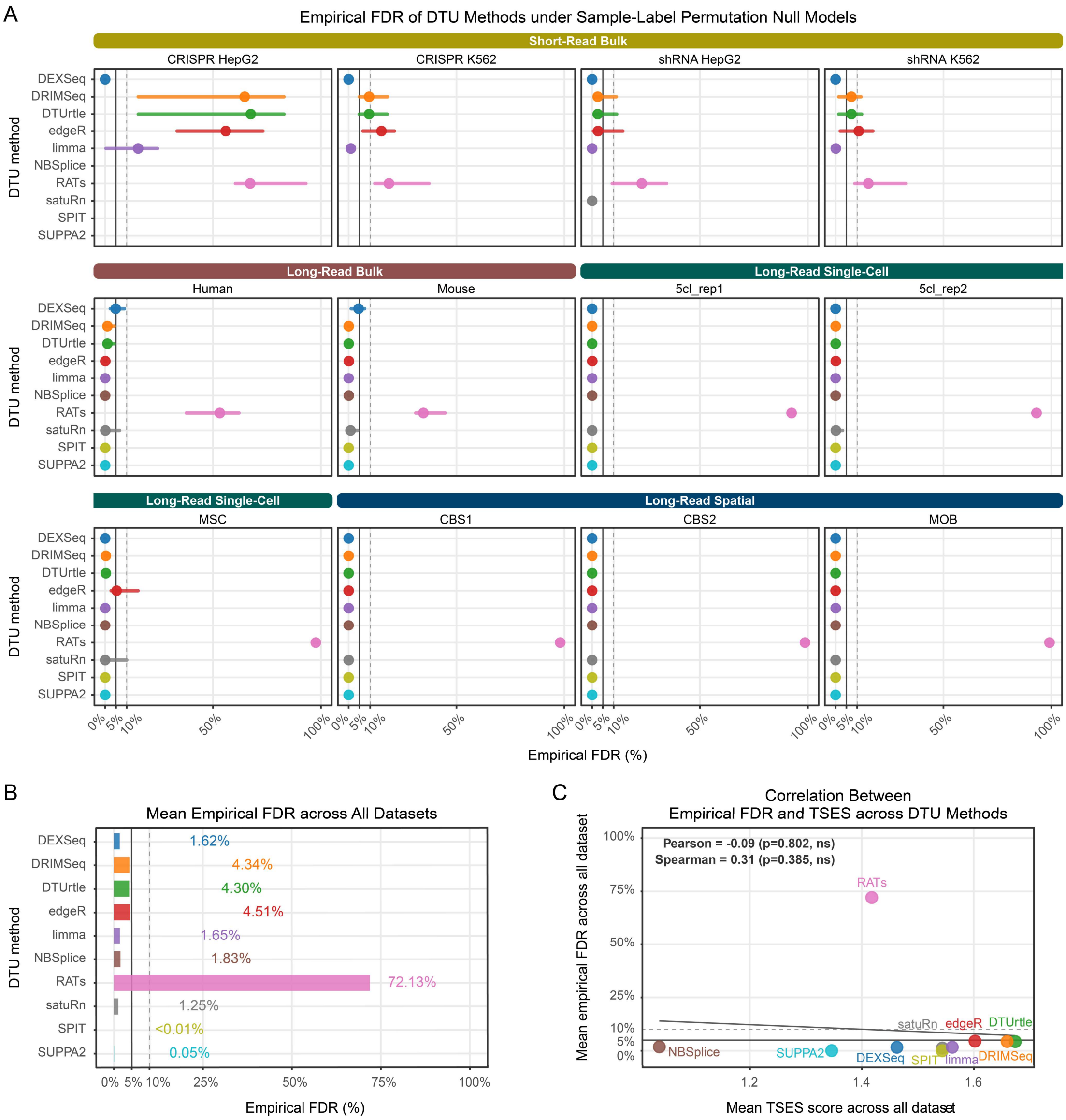

Figure S1. Empirical FDR of DTU methods under sample-label permutation null models. Empirical FDR is calculated as described in the Materials and Methods. Solid and dashed reference lines indicate 5% and 10% FDR. **(A) Empirical FDR of different DTU methods across multiple datasets.** The x-axis represents the empirical FDR, and the y-axis shows the DTU methods. Points indicate the median empirical FDR across contrasts within each dataset, and horizontal bars indicate the interquartile range. **(B) Mean empirical FDR of different DTU methods across all datasets.** The x-axis represents the empirical FDR, and the y-axis shows the DTU methods. Horizontal bars represent the mean empirical FDR across all datasets and contrasts. **(C) Correlation between empirical FDR and TSES score across DTU methods.** The x-axis represents the mean TSES score across all datasets, and the y-axis represents the mean empirical FDR. Each point indicates one DTU method, and the grey line shows a linear fit. No significant correlation is observed between empirical FDR and TSES score across methods.

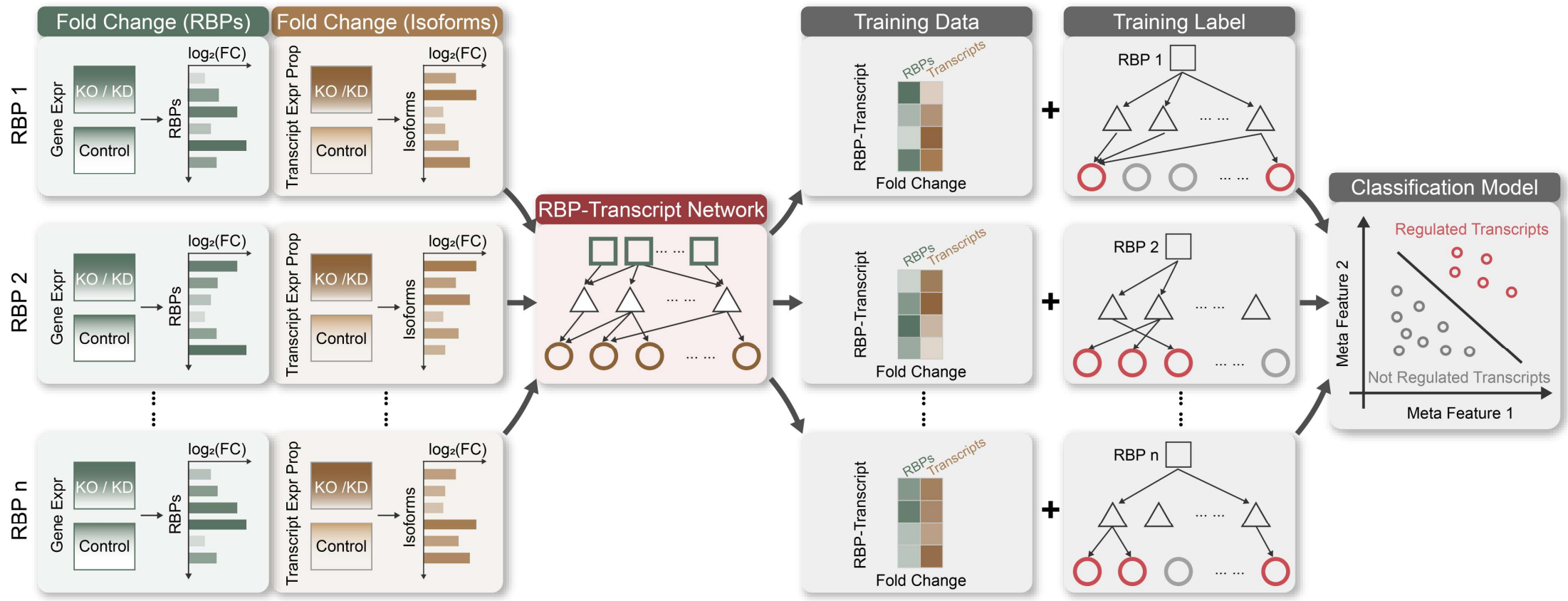

Figure S2. Workflow for predicting regulated transcripts. Taking the RBP 1 knockout dataset as an example, we use the fold change of RBP gene expression and transcript expression proportion between the KO/KD and control groups as the weights of the RBP and transcript nodes in the RBP-transcript network. All RBP-transcript edges are extracted as training data, and the known regulated transcript set of RBP 1 is used as training labels. We then combine the training data and labels from all RBPs (RBP 1, RBP 2, …, RBP n) and input them into a GLM model to train the prediction model for regulated transcripts. In the RBP-transcript network diagram, green boxes represent RBPs, yellow circles represent transcripts, and triangles indicate RBP binding sites. In the training label diagram, red circles represent transcripts known to be regulated by RBPs.

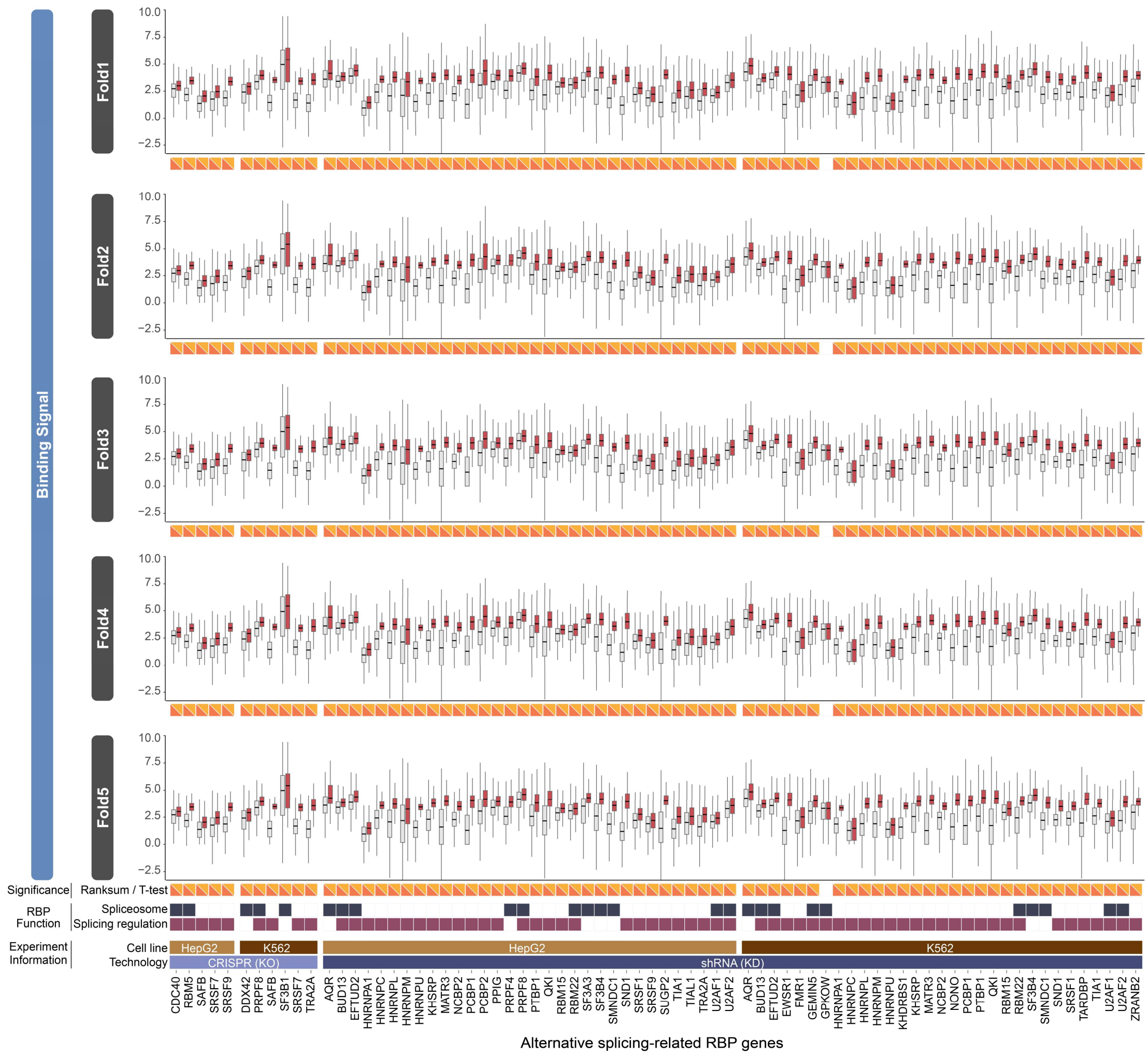

Figure S3. Validation of regulated transcript sets across five-fold cross-validation. The reliability of fold-specific GLM-predicted regulated transcript sets is tested by comparing RBP binding signals between transcripts within and outside each predicted set in ENCODE RBP KO/KD datasets. For each fold, the regulated transcript set contains transcripts predicted as regulated by the GLM in five-fold cross-validation. In each boxplot, red boxes represent transcripts within the corresponding fold-specific predicted regulated transcript set, while grey boxes represent transcripts outside the set among transcripts quantified for the same RBP KO/KD dataset. The x-axis shows alternative splicing-related RBP genes, and the y-axis shows mean peak signal. Annotation bars indicate experimental technology, cell line, RBP function, and significance from one-sided t-test and Wilcoxon rank-sum test.

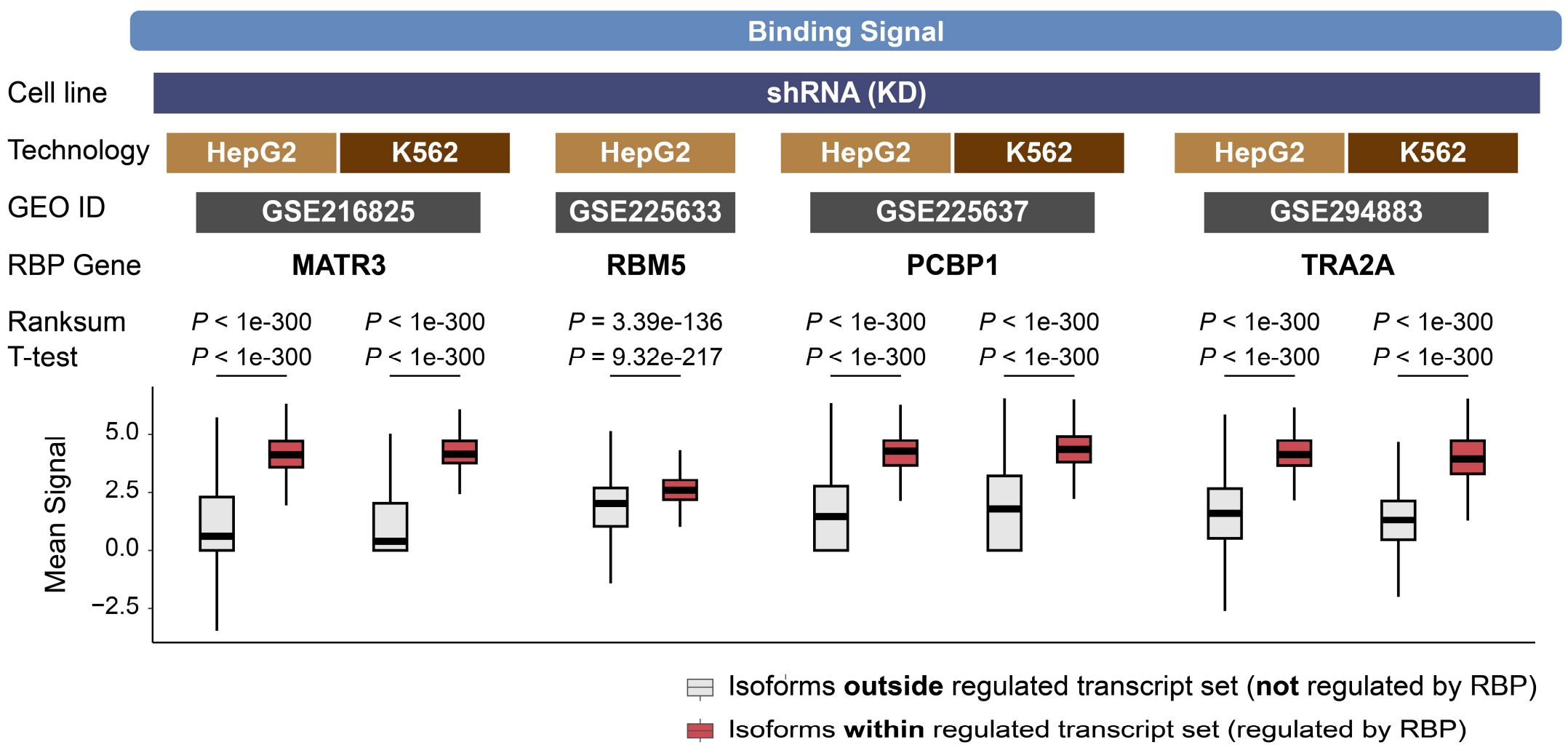

Figure S4. Validation of regulated transcript sets in independent RBP knockdown datasets. The reliability of regulated transcript sets is tested in four independent RBP knockdown GEO datasets. Regulated transcript sets for MATR3, RBM5, PCBP1, and TRA2A are predicted by the GLM model and are compared with transcripts outside the corresponding set. RBP binding signals are obtained from available ENCODE HepG2 or K562 peak-signal profiles for the same RBP. In each boxplot, red boxes represent transcripts within the predicted regulated transcript set, while grey boxes represent transcripts outside the set. The y-axis shows mean peak signal. Annotation bars indicate knockdown technology, cell line, GEO ID, and RBP gene. P-values from one-sided t-test and Wilcoxon rank-sum test assess whether transcripts within the predicted regulated transcript set show higher binding signal than transcripts outside the set.

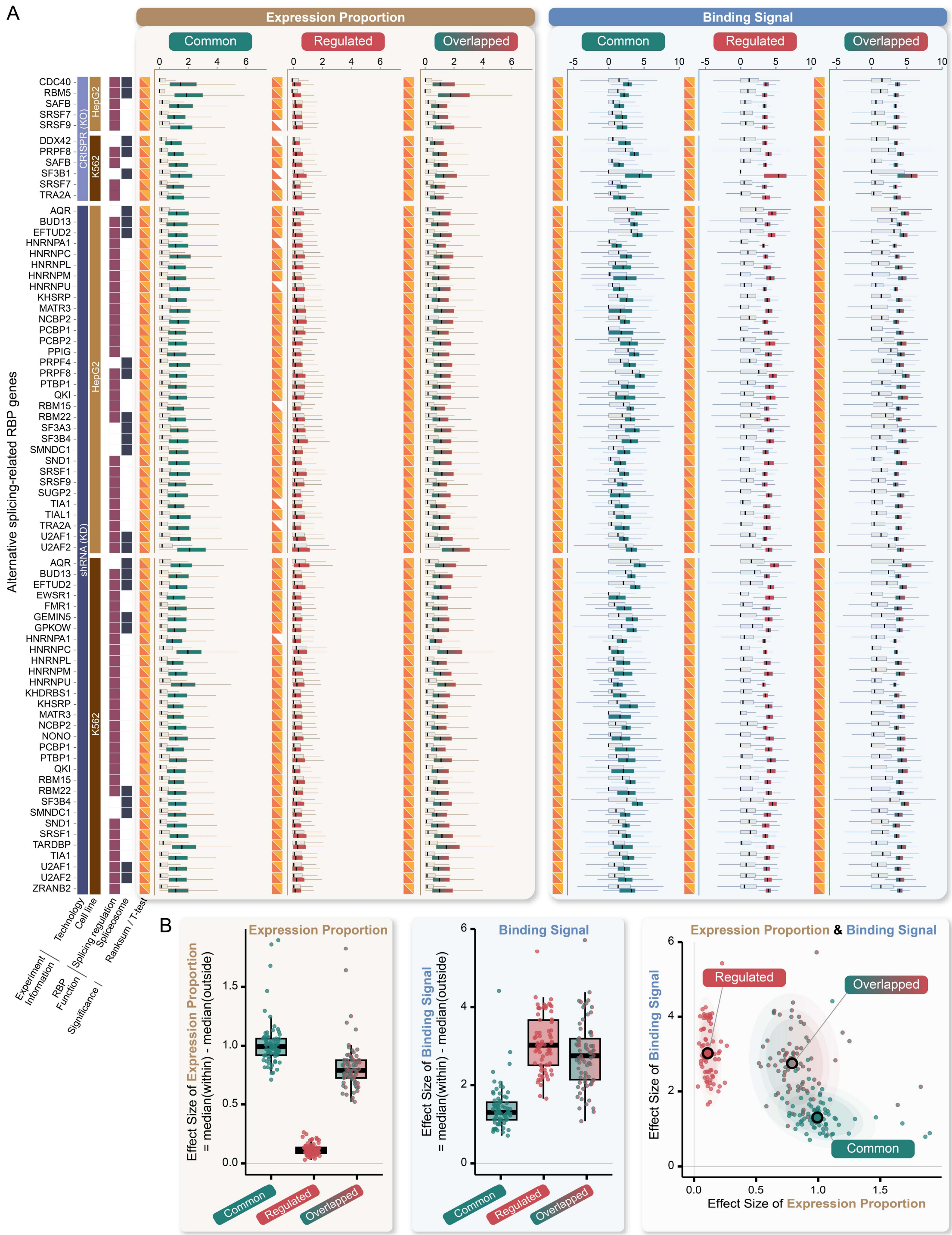

Figure S5. Quantitative validation of reference transcript sets under the leave-one-method-out (LOMO) setting. Under the LOMO setting, one DTU method is omitted when generating the common transcript set, and the overlapped transcript set is updated accordingly. The reliability of the resulting reference transcript sets is tested by comparing transcripts within and outside each set in RBP KO/KD datasets. **(A) Statistical validation of common, regulated, and overlapped transcript sets.** In each boxplot, colored boxes represent transcripts within the corresponding set, while grey boxes represent transcripts outside the set. The y-axis represents RBP KO/KD datasets, and the x-axis shows expression proportion fold change or binding signal. Annotation bars indicate experimental technology, cell line, RBP function, and significance from t-test and rank-sum test. **(B) Effect size of expression proportion fold change and binding signal.** Effect size is calculated as median(within) minus median(outside) for each RBP KO/KD dataset and each reference transcript set. Boxplots summarize effect size values, and the scatter plot shows the relationship between the effect size of expression proportion fold change and the effect size of binding signal.

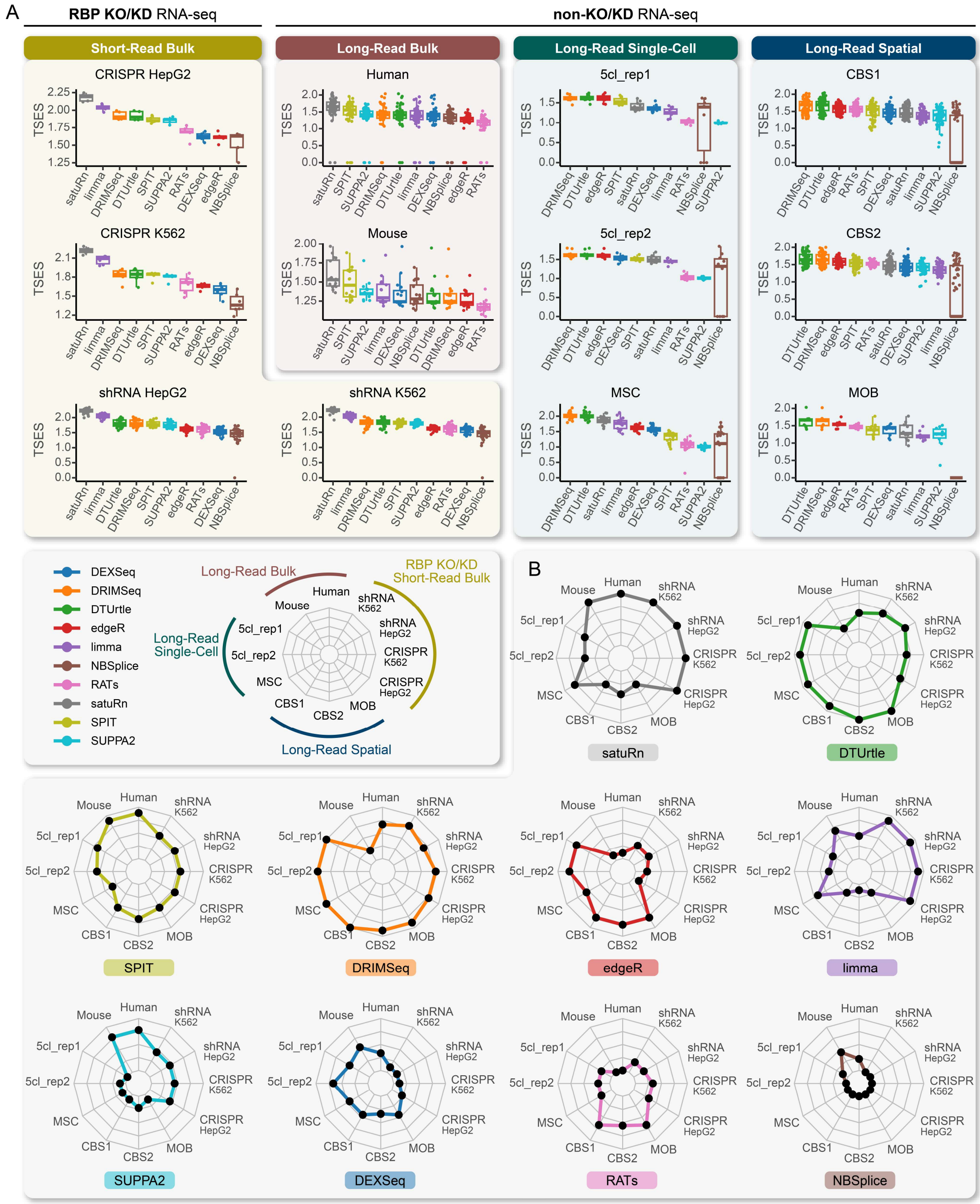

Figure S6. Evaluation results of ten representative DTU methods under the leave-one-method-out (LOMO) setting. Under the LOMO setting, when a DTU method is evaluated, that method is excluded from the generation of the reference transcript set. The common transcript set is then constructed using the remaining DTU methods, and the overlapped transcript set is updated accordingly. **(A) TSES values of different DTU methods across multiple datasets.** The y-axis represents the TSES values, and the x-axis shows the DTU methods. Each dot in the boxplot indicates the TSES value of one condition pair within a dataset. Methods on the x-axis are ranked by their mean TSES values. **(B) Ranking of DTU methods based on TSES across different datasets.** In the radar chart, a point farther from the center indicates a higher ranking of the corresponding method in that dataset.

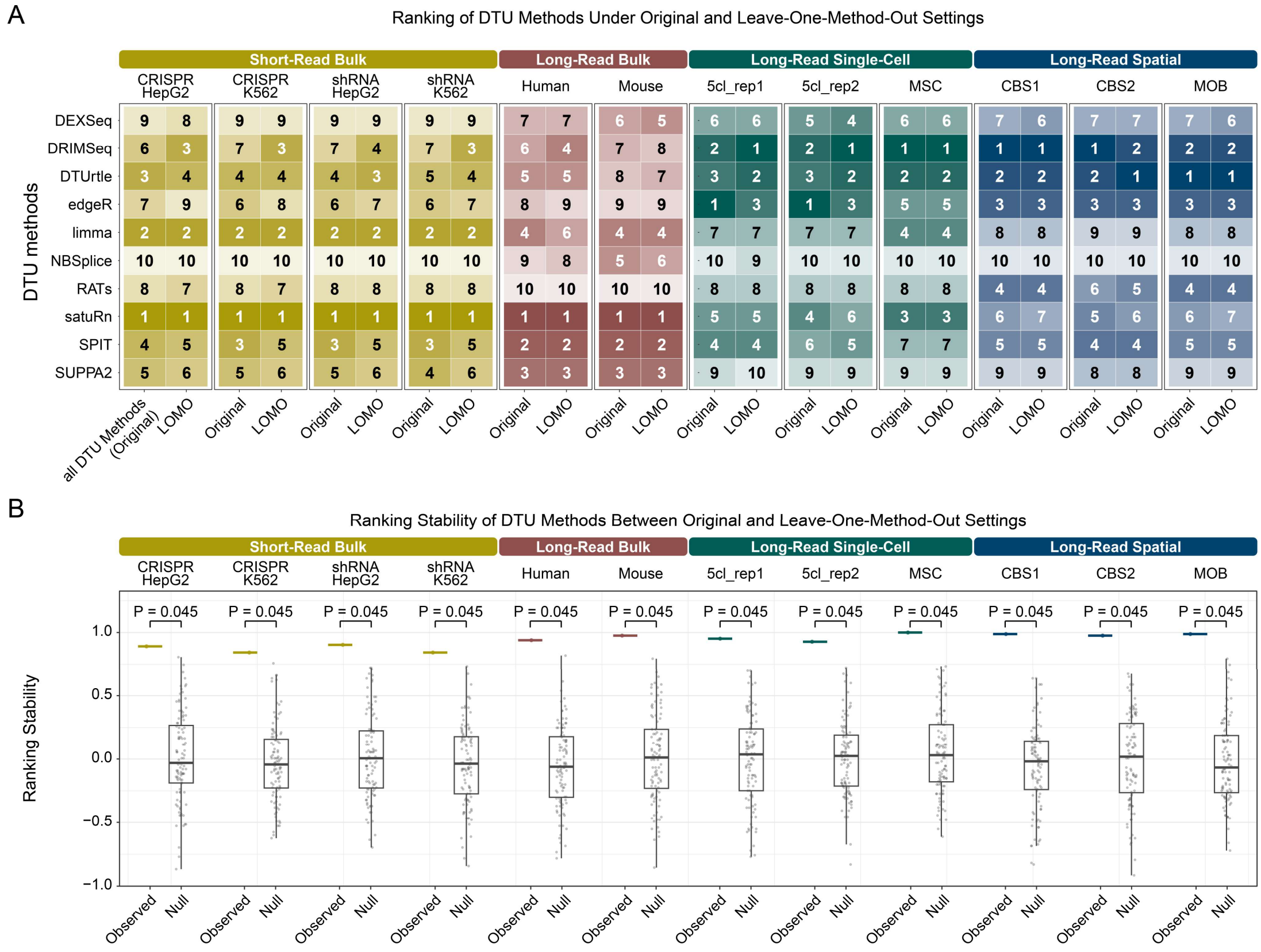

Figure S7. Ranking robustness of ten representative DTU methods under original and leave-one-method-out settings. (A) DTU method rankings across original and LOMO settings. The heatmap shows the ranks of ten DTU methods under the original and LOMO settings for each dataset. Rows indicate DTU methods, columns indicate original and LOMO settings, and numbers in the tiles indicate method ranks. **(B) Ranking stability relative to the original setting.** Observed ranking stability represents the Spearman correlation between the method ranking under the original setting and the ranking under the LOMO setting. Null distributions represent Spearman correlations between randomly permuted method rankings and are generated from 100 random permutations for the LOMO comparison, yielding 100 null correlations per dataset. P-values are based on one-sided Wilcoxon rank-sum tests for observed values greater than null values.

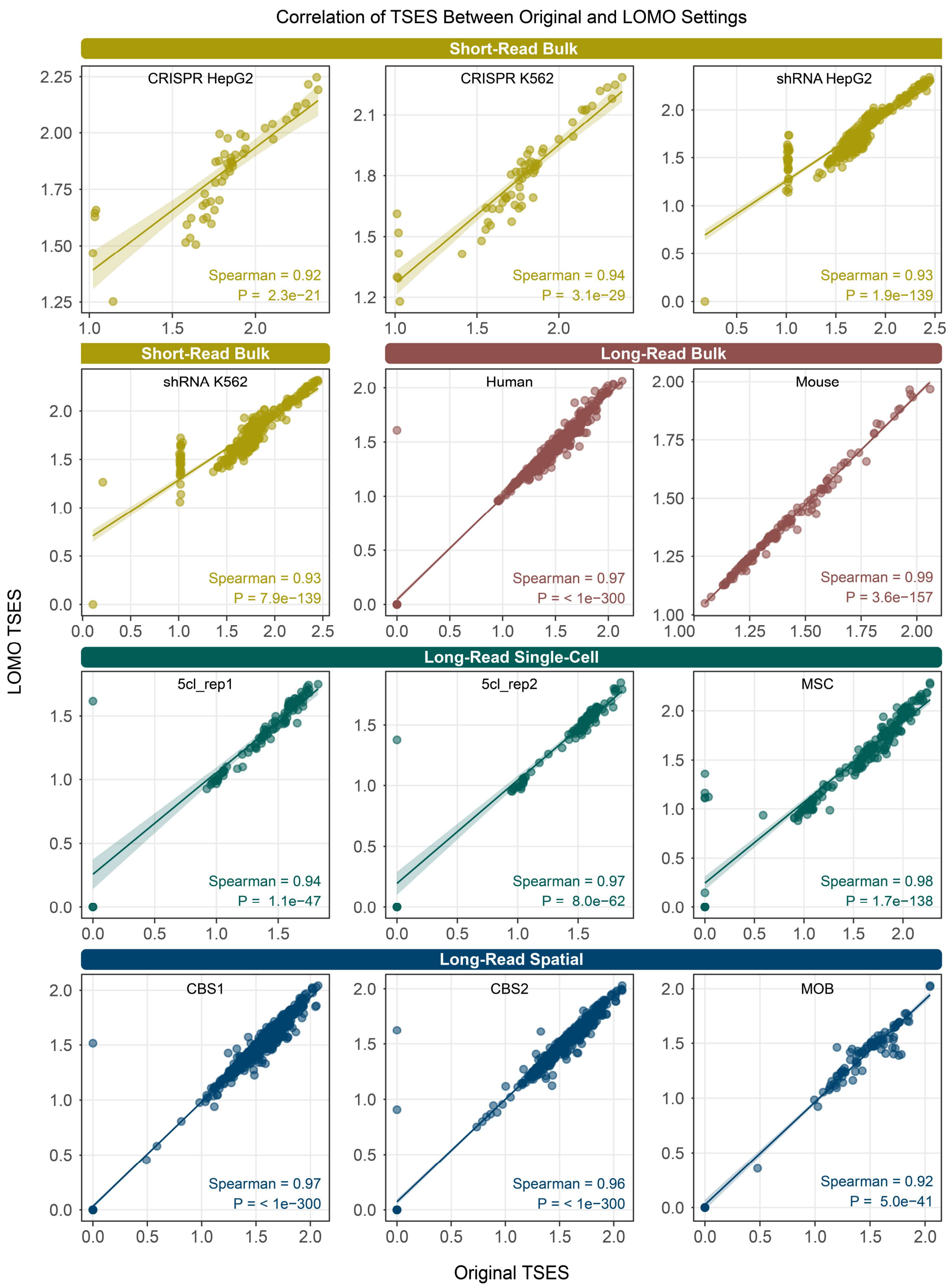

Figure S8. Similarity of TSES scores based on reference transcript sets obtained from the original and leave-one-method-out (LOMO) settings. Under the LOMO setting, when a DTU method is evaluated, that method is excluded from the generation of the reference transcript set. The x-axis shows TSES values calculated from the original reference transcript sets constructed using all DTU methods, and the y-axis shows those calculated from the LOMO reference transcript sets. Each dot represents paired TSES values for one condition pair and one DTU method within a dataset.

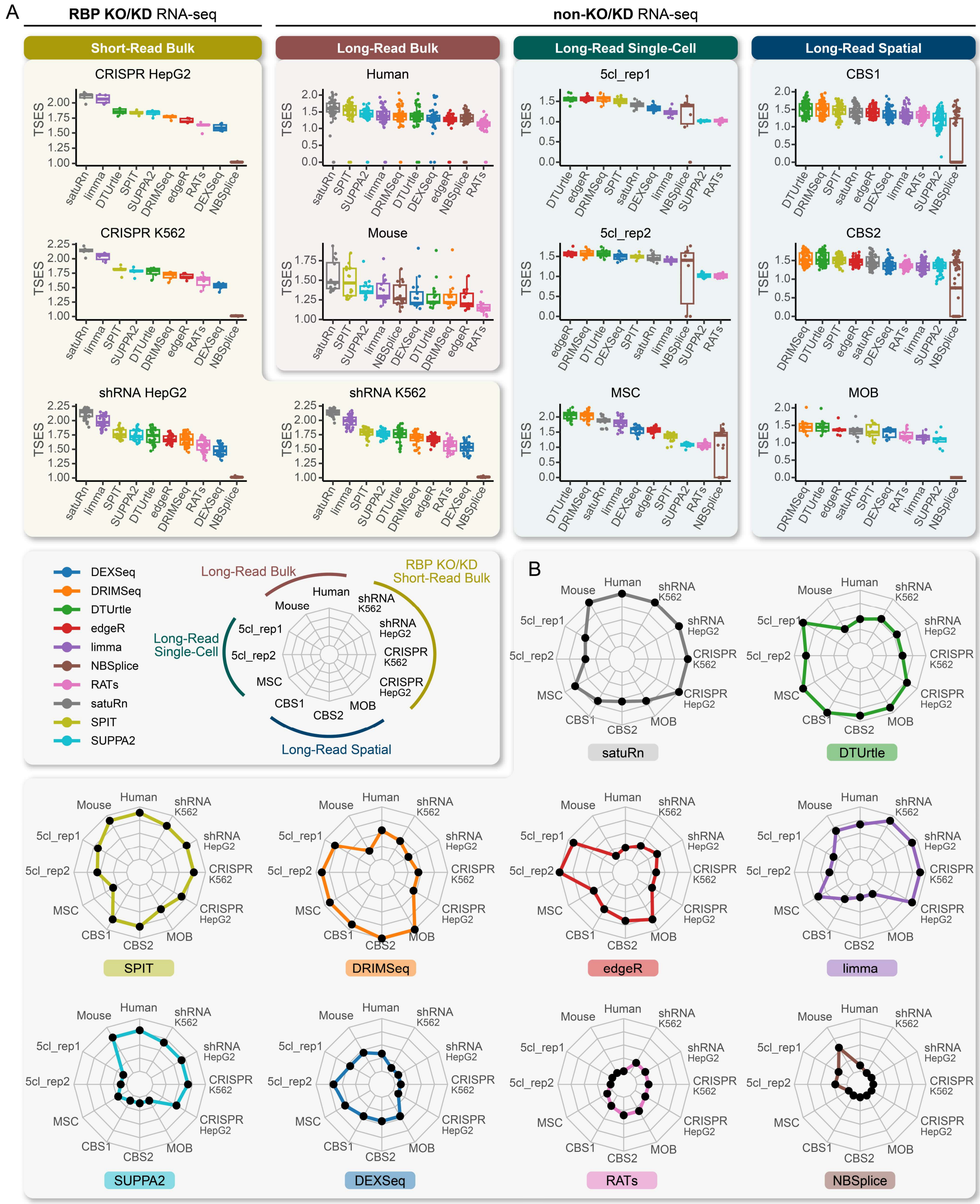

Figure S9. Evaluation results of ten representative DTU methods under the common-threshold parameter setting of comThre = 3. (A) TSES values of different DTU methods across multiple datasets. The y-axis represents the TSES values, and the x-axis shows the DTU methods. Each dot in the boxplot indicates the TSES value of one condition pair within a dataset. Methods on the x-axis are ranked by their mean TSES values. **(B) Ranking of DTU methods based on TSES across different datasets.** In the radar chart, a point farther from the center indicates a higher ranking of the corresponding method in that dataset.

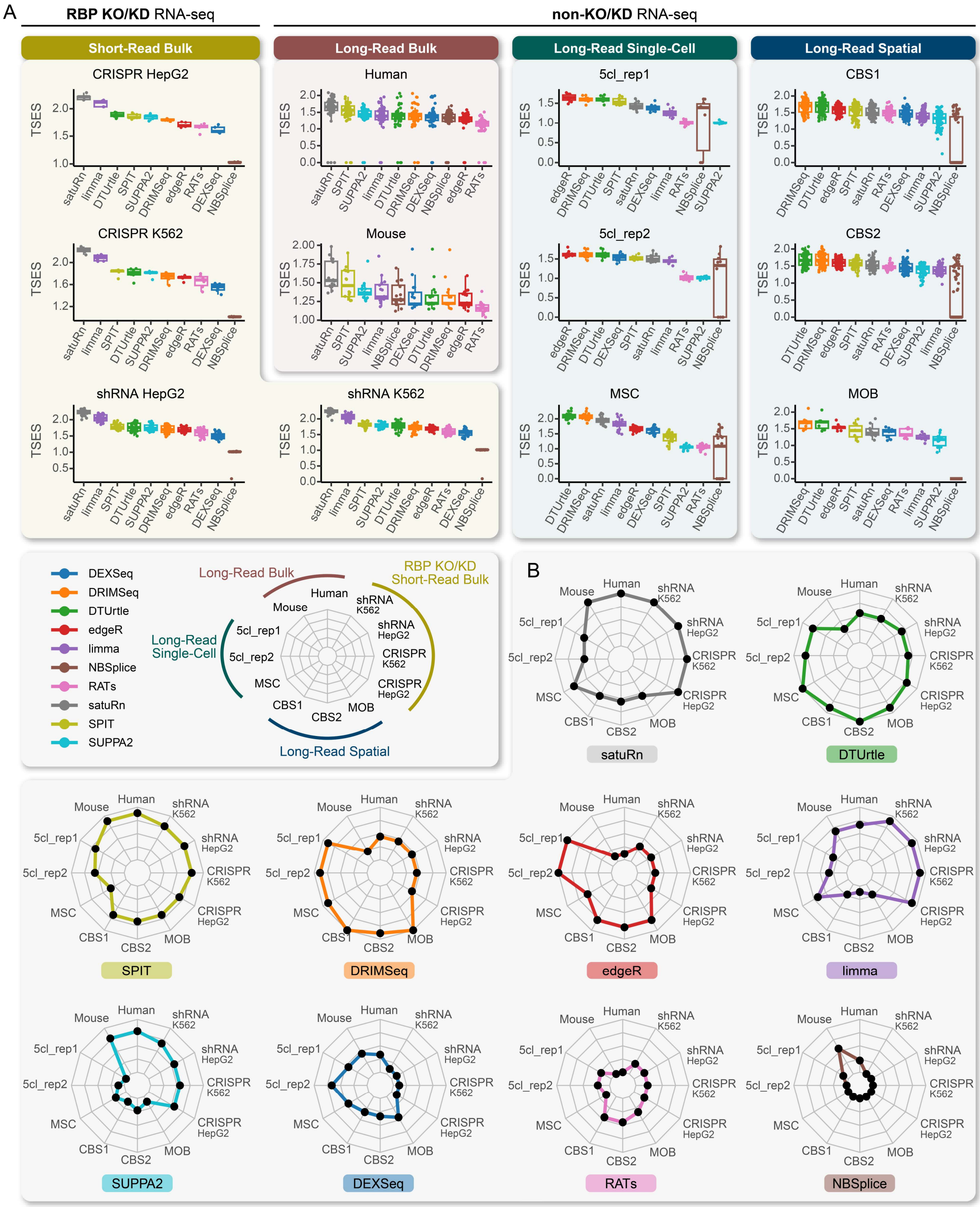

Figure S10. Evaluation results of ten representative DTU methods under the common-threshold parameter setting of comThre = 4. (A) TSES values of different DTU methods across multiple datasets. The y-axis represents the TSES values, and the x-axis shows the DTU methods. Each dot in the boxplot indicates the TSES value of one condition pair within a dataset. Methods on the x-axis are ranked by their mean TSES values. **(B) Ranking of DTU methods based on TSES across different datasets.** In the radar chart, a point farther from the center indicates a higher ranking of the corresponding method in that dataset.

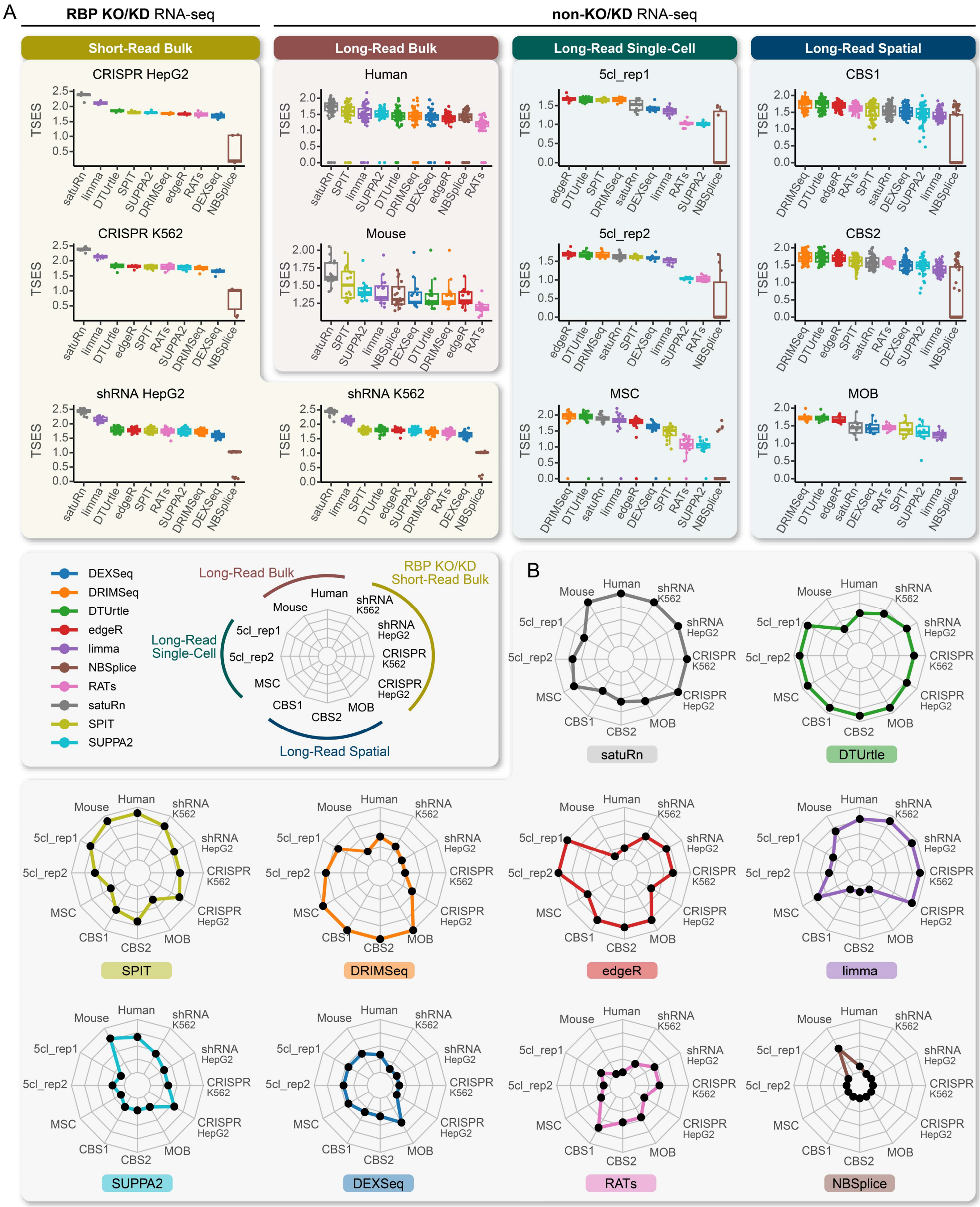

Figure S11. Evaluation results of ten representative DTU methods under the common-threshold parameter setting of comThre = 6. (A) TSES values of different DTU methods across multiple datasets. The y-axis represents the TSES values, and the x-axis shows the DTU methods. Each dot in the boxplot indicates the TSES value of one condition pair within a dataset. Methods on the x-axis are ranked by their mean TSES values. **(B) Ranking of DTU methods based on TSES across different datasets.** In the radar chart, a point farther from the center indicates a higher ranking of the corresponding method in that dataset.

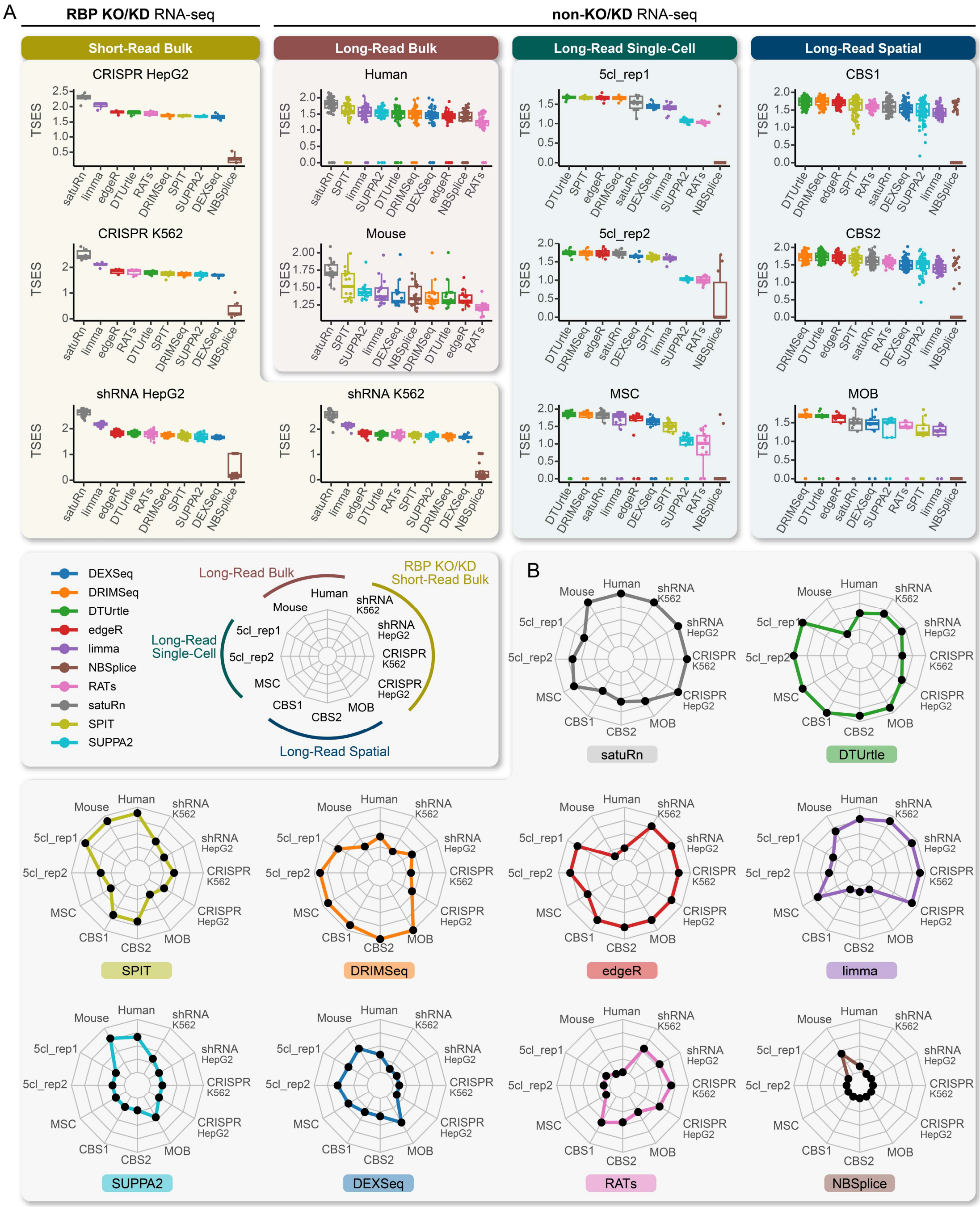

Figure S12. Evaluation results of ten representative DTU methods under the common-threshold parameter setting of comThre = 7. (A) TSES values of different DTU methods across multiple datasets. The y-axis represents the TSES values, and the x-axis shows the DTU methods. Each dot in the boxplot indicates the TSES value of one condition pair within a dataset. Methods on the x-axis are ranked by their mean TSES values. **(B) Ranking of DTU methods based on TSES across different datasets.** In the radar chart, a point farther from the center indicates a higher ranking of the corresponding method in that dataset.

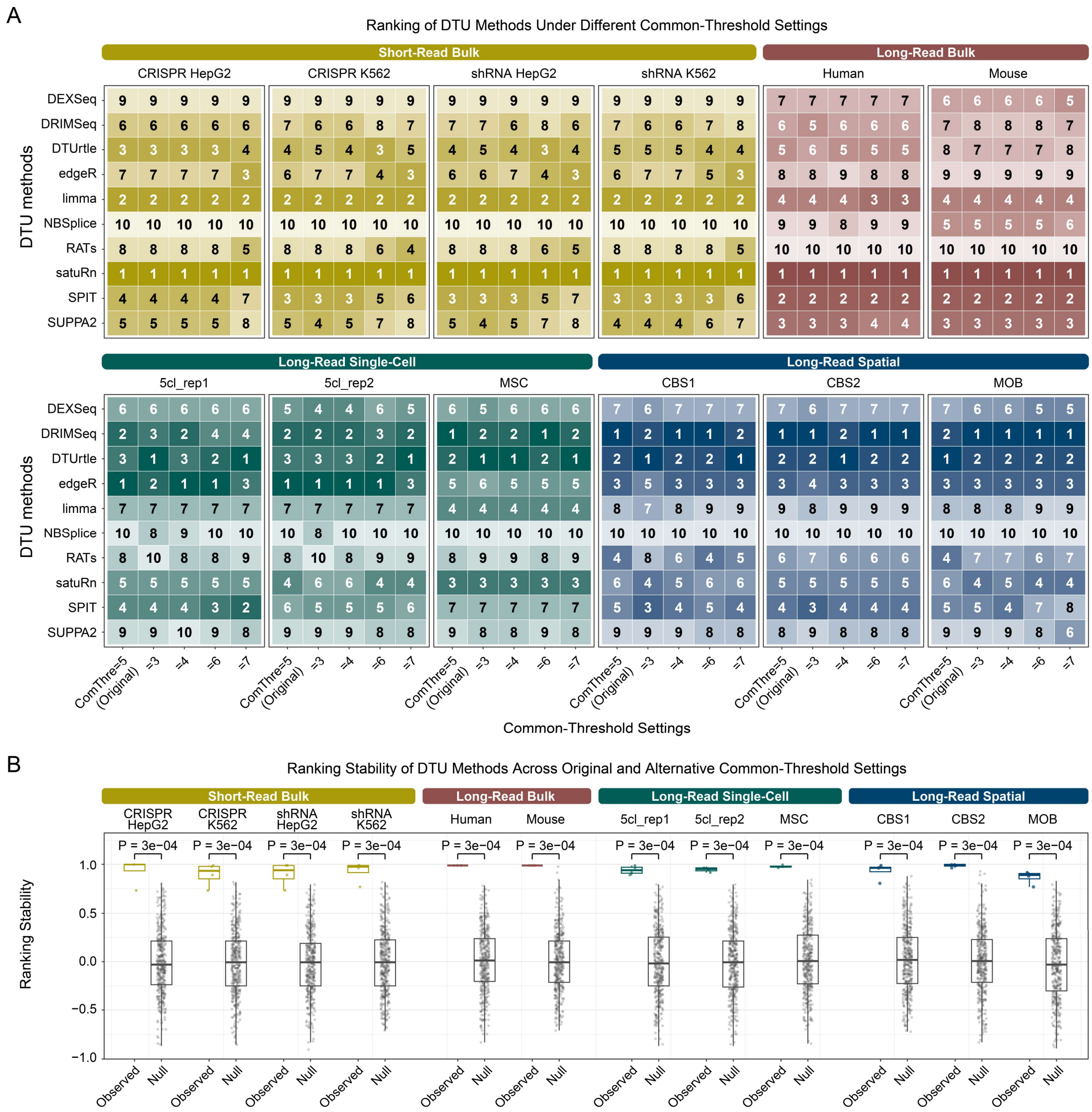

Figure S13. Ranking robustness of ten representative DTU methods under original and alternative common-threshold settings. (A) DTU method rankings across common-threshold settings. The heatmap shows the ranks of ten DTU methods under the original and alternative common-threshold settings (common-threshold = 3, 4, 6, and 7) for each dataset. Rows indicate DTU methods, columns indicate common-threshold settings, and numbers in the tiles indicate method ranks. **(B) Ranking stability relative to the original setting.** Observed ranking stability represents the Spearman correlation between the method ranking under the original setting (common-threshold = 5) and the ranking under each alternative setting. Null distributions represent Spearman correlations between randomly permuted method rankings, generated from 100 random permutations for each of the four alternative-setting comparisons (common-threshold = 3, 4, 6, and 7), yielding 100 × 4 = 400 null correlations per dataset. P-values are based on one-sided Wilcoxon rank-sum tests for observed values greater than null values.

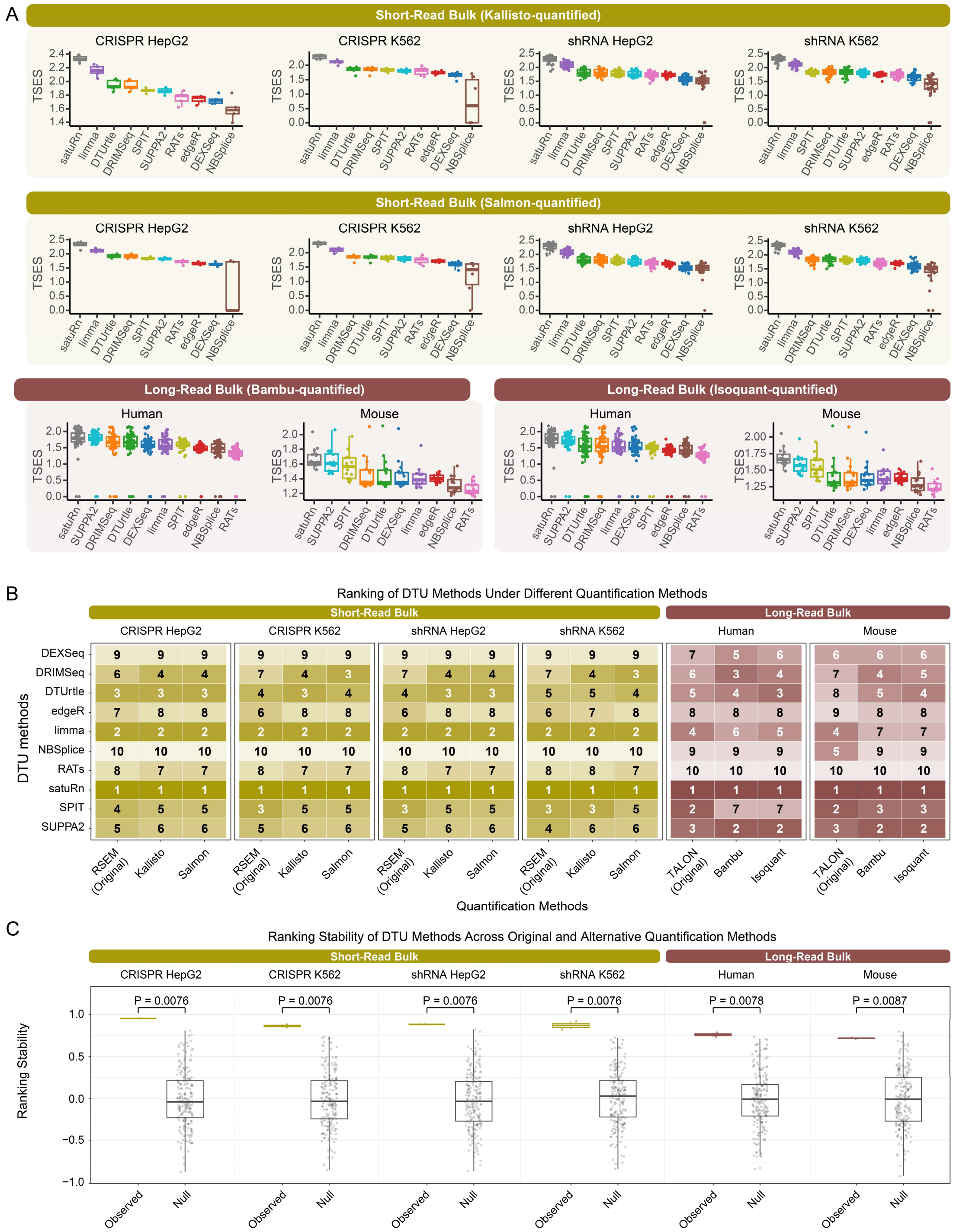

Figure S14. Performance and ranking robustness of ten representative DTU methods under alternative transcript quantification methods. (A) TSES values of ten DTU methods under alternative quantification methods (including Kallisto and Salmon for short-read bulk datasets, and Bambu and Isoquant for long-read bulk RNA-seq datasets). Boxplots summarize TSES distributions across condition pairs within each data subtype, and each dot represents one condition pair. Higher TSES values indicate stronger enrichment of reference transcripts near the top of DTU-ranked transcript lists. (B) Ranking of DTU methods across original and alternative quantification methods. For each data subtype and quantification method, DTU methods are ranked by mean TSES. Numbers in heatmap tiles indicate method ranks, with rank 1 representing the best-performing method. (C) Ranking stability between original and alternative quantification methods. Observed ranking stability represents the Spearman correlation between DTU method ranks from original quantification and those from each alternative quantification method. Null distributions represent Spearman correlations between randomly permuted method ranks, generated using 100 random

permutations for each original-alternative comparison. P-values are calculated using one-sided Wilcoxon rank-sum tests for observed values greater than null values.

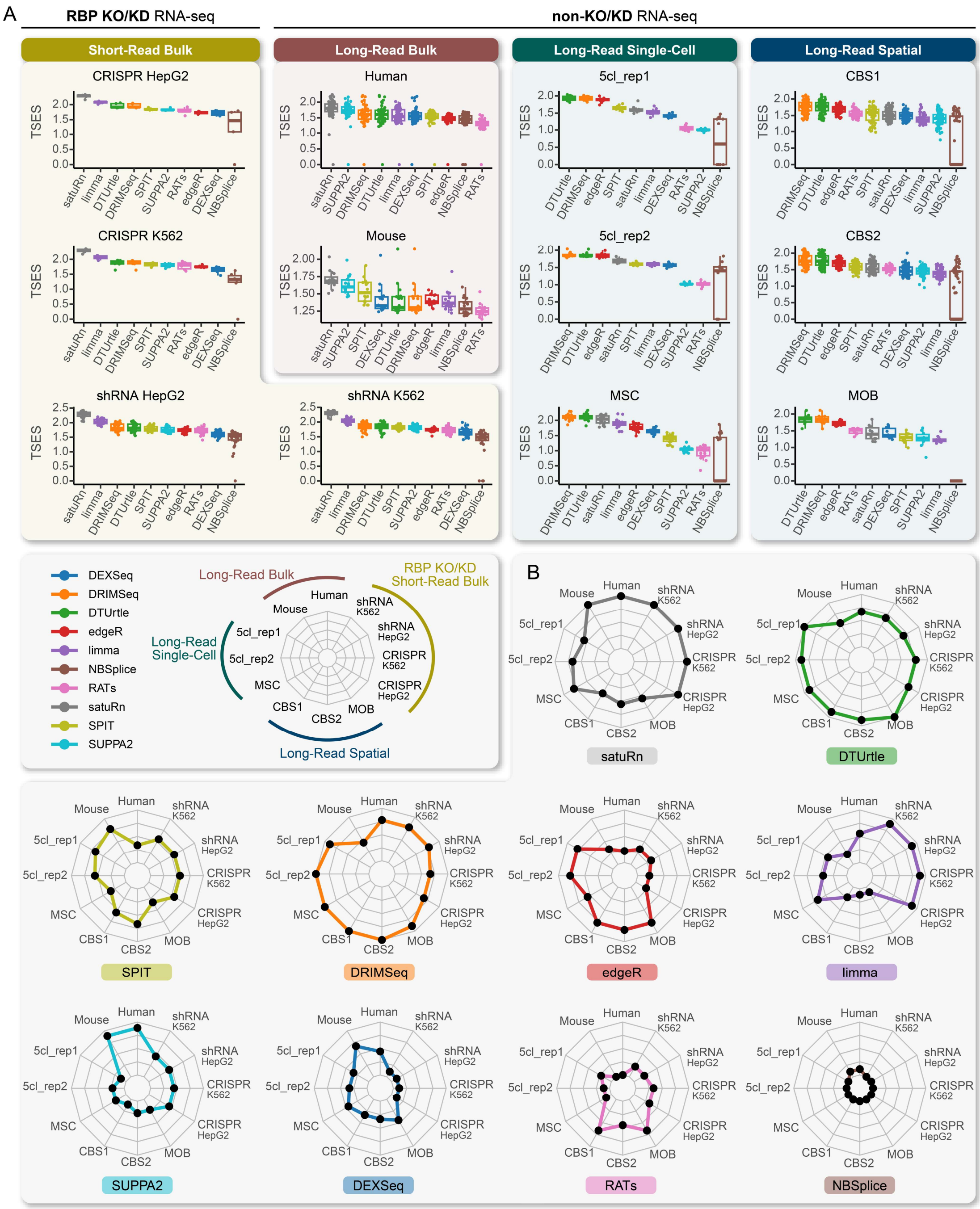

Figure S15. Evaluation results of ten representative DTU methods under the expression-filtering parameter setting of minExp = 2 and minSample = 2. (A) TSES values of different DTU methods across multiple datasets. The y-axis represents the TSES values, and the x-axis shows the DTU methods. Each dot in the boxplot indicates the TSES value of one condition pair within a dataset. Methods on the x-axis are ranked by their mean TSES values. **(B) Ranking of DTU methods based on TSES across different datasets.** In the radar chart, a point farther from the center indicates a higher ranking of the corresponding method in that dataset.

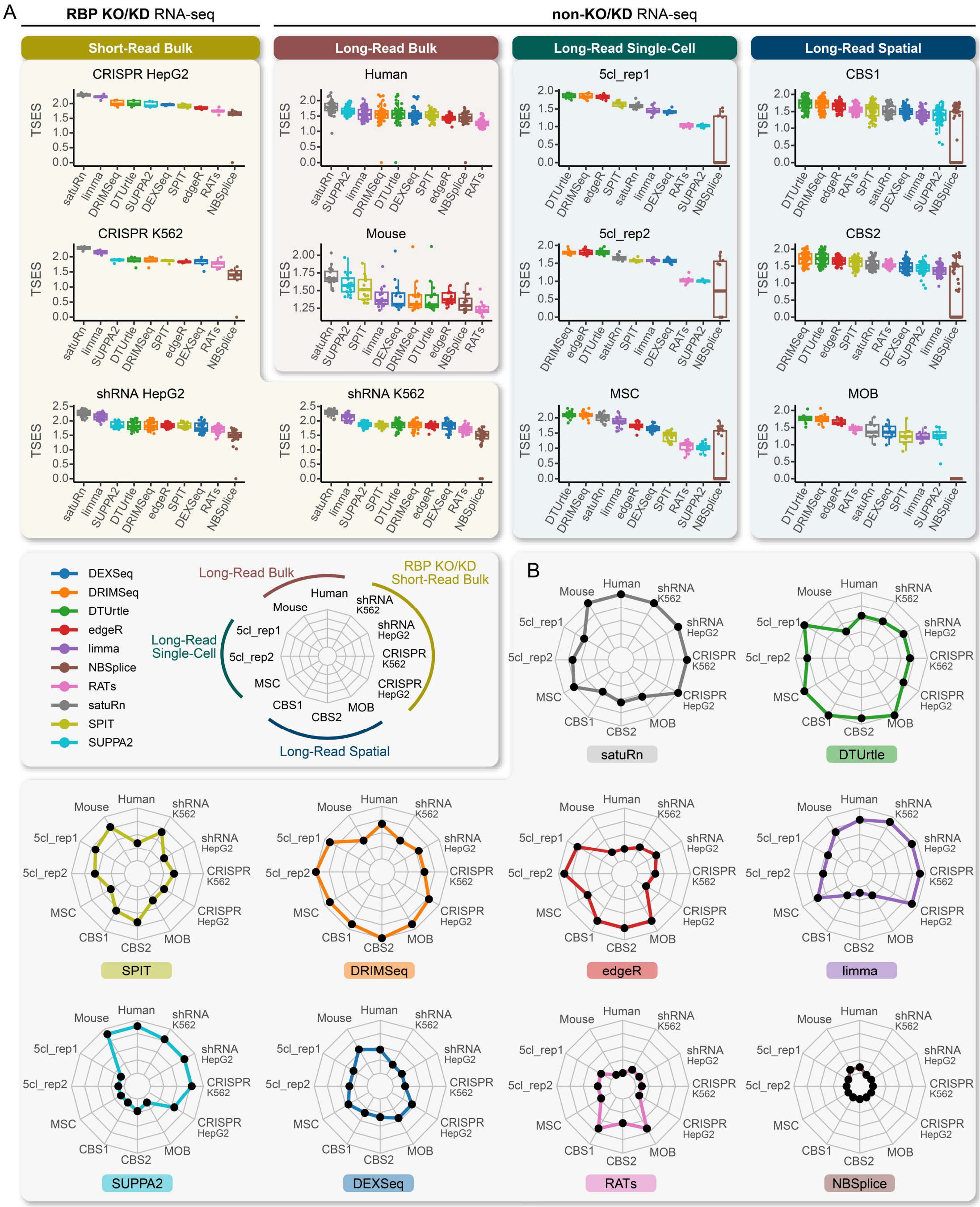

Figure S16. Evaluation results of ten representative DTU methods under the expression-filtering parameter setting of minExp = 2 and minSample = 3. (A) TSES values of different DTU methods across multiple datasets. The y-axis represents the TSES values, and the x-axis shows the DTU methods. Each dot in the boxplot indicates the TSES value of one condition pair within a dataset. Methods on the x-axis are ranked by their mean TSES values. **(B) Ranking of DTU methods based on TSES across different datasets.** In the radar chart, a point farther from the center indicates a higher ranking of the corresponding method in that dataset.

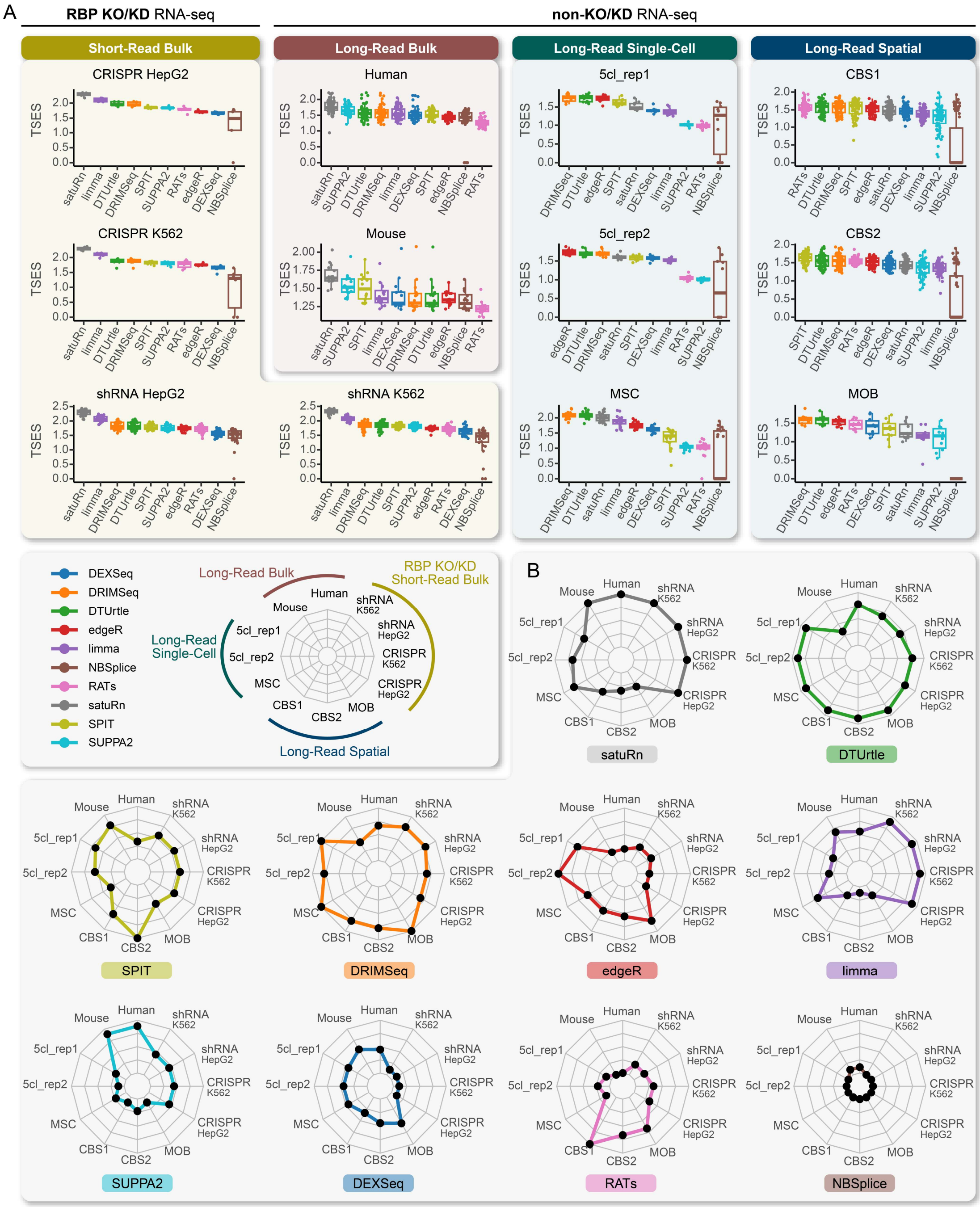

Figure S17. Evaluation results of ten representative DTU methods under the expression-filtering parameter setting of minExp = 3 and minSample = 2. (A) TSES values of different DTU methods across multiple datasets. The y-axis represents the TSES values, and the x-axis shows the DTU methods. Each dot in the boxplot indicates the TSES value of one condition pair within a dataset. Methods on the x-axis are ranked by their mean TSES values. **(B) Ranking of DTU methods based on TSES across different datasets.** In the radar chart, a point farther from the center indicates a higher ranking of the corresponding method in that dataset.

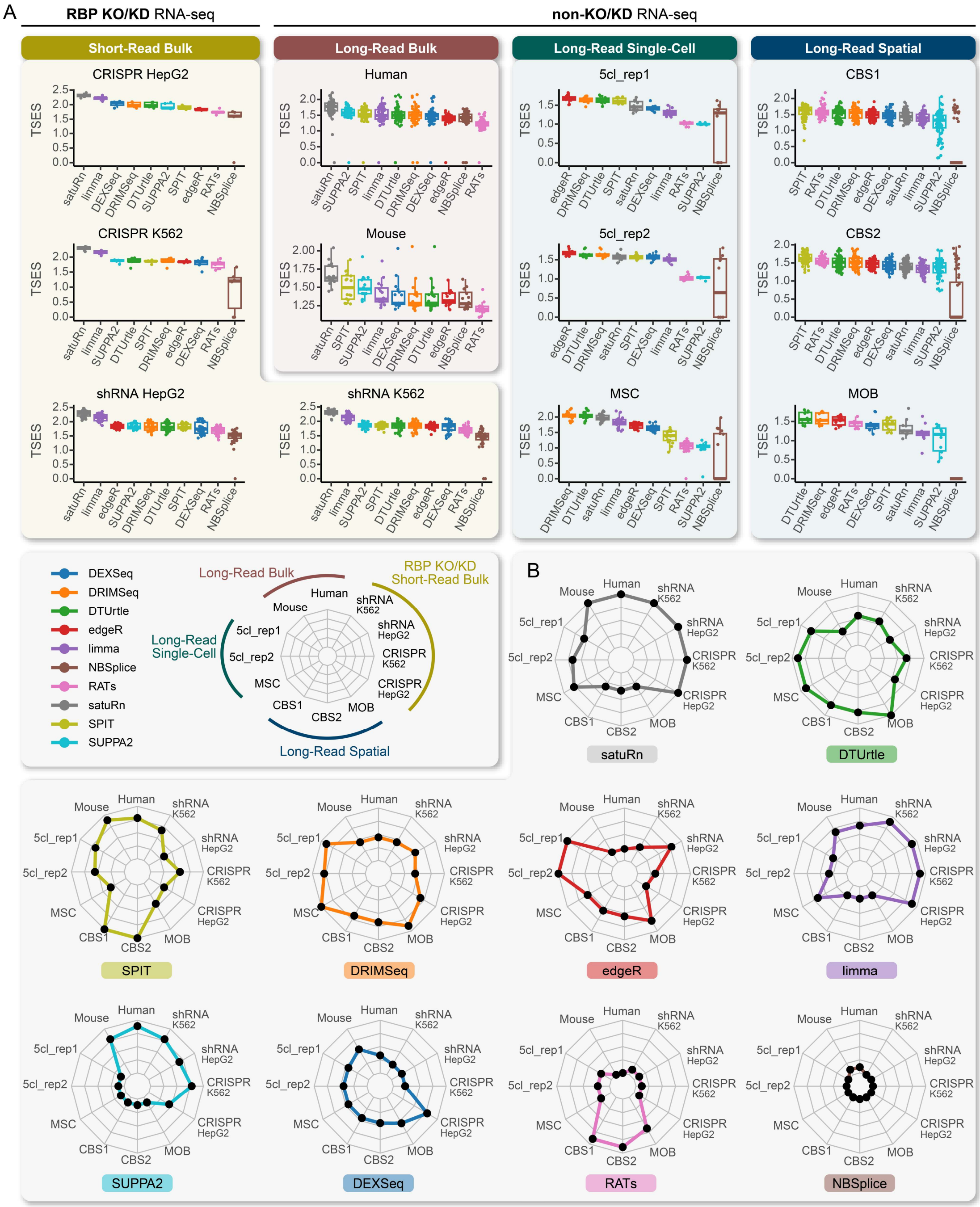

Figure S18. Evaluation results of ten representative DTU methods under the expression-filtering parameter setting of minExp = 3 and minSample = 3. (A) TSES values of different DTU methods across multiple datasets. The y-axis represents the TSES values, and the x-axis shows the DTU methods. Each dot in the boxplot indicates the TSES value of one condition pair within a dataset. Methods on the x-axis are ranked by their mean TSES values. **(B) Ranking of DTU methods based on TSES across different datasets.** In the radar chart, a point farther from the center indicates a higher ranking of the corresponding method in that dataset.

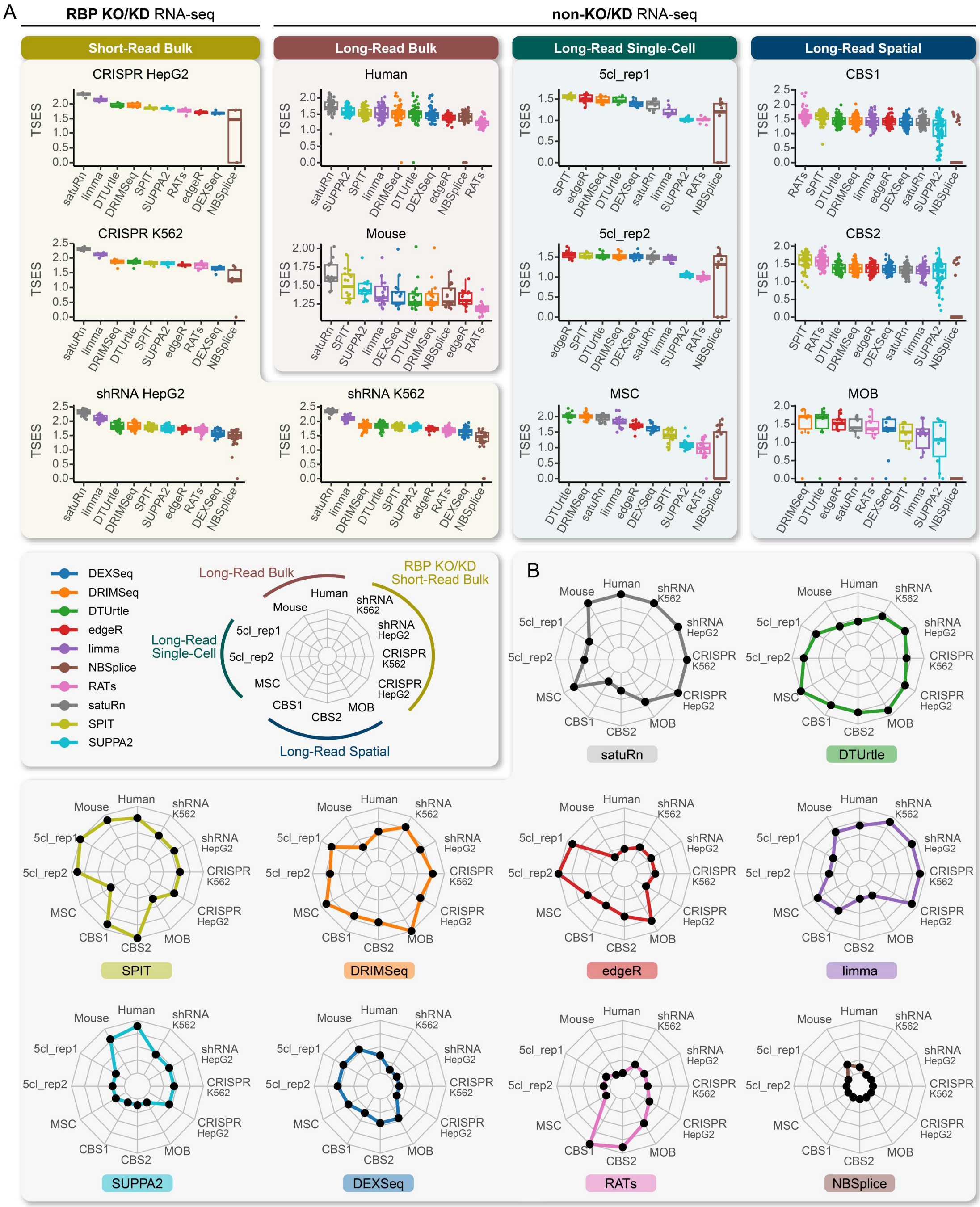

Figure S19. Evaluation results of ten representative DTU methods under the expression-filtering parameter setting of minExp = 5 and minSample = 2. (A) TSES values of different DTU methods across multiple datasets. The y-axis represents the TSES values, and the x-axis shows the DTU methods. Each dot in the boxplot indicates the TSES value of one condition pair within a dataset. Methods on the x-axis are ranked by their mean TSES values. **(B) Ranking of DTU methods based on TSES across different datasets.** In the radar chart, a point farther from the center indicates a higher ranking of the corresponding method in that dataset.

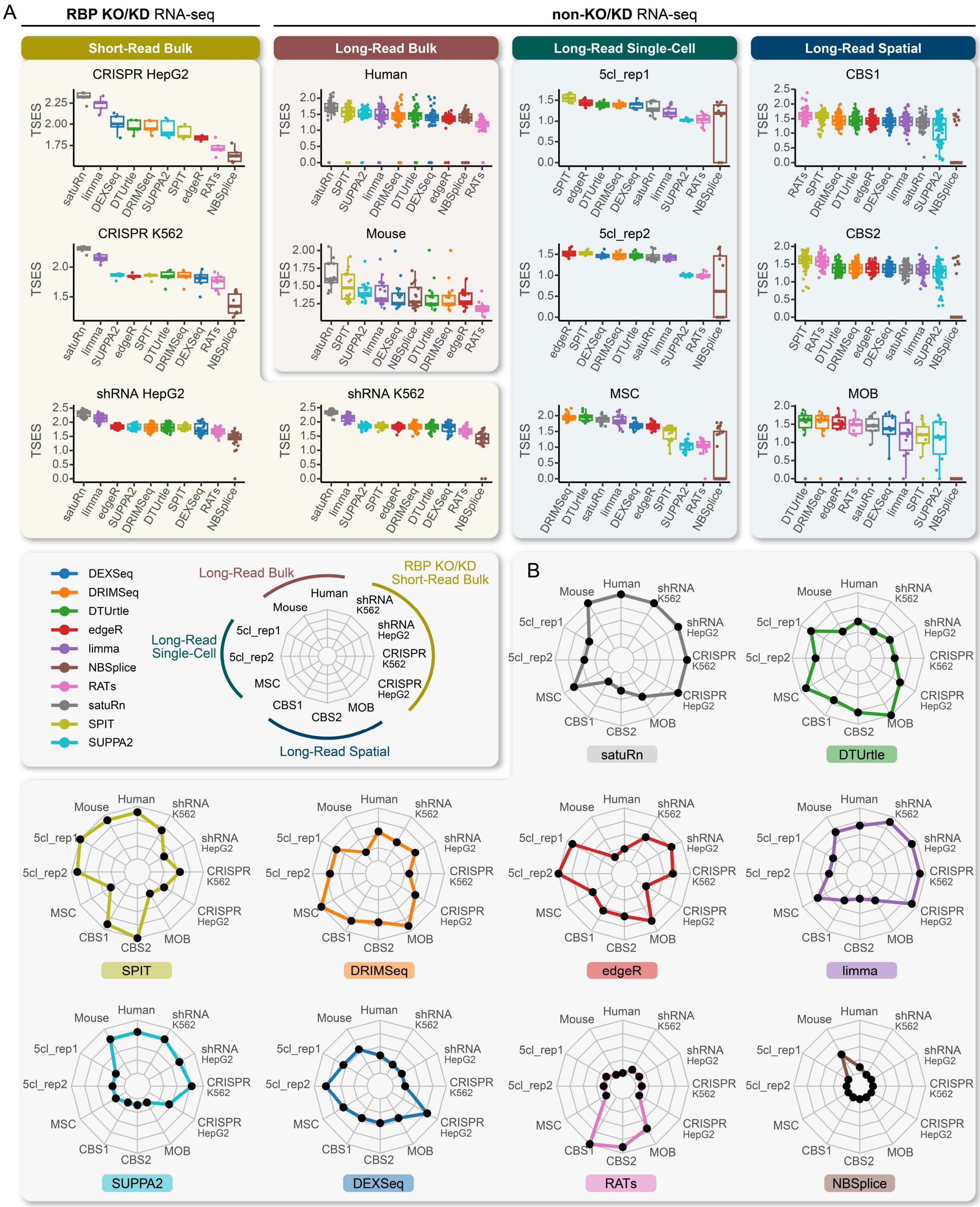

Figure S20. Evaluation results of ten representative DTU methods under the expression-filtering parameter setting of minExp = 5 and minSample = 3. (A) TSES values of different DTU methods across multiple datasets. The y-axis represents the TSES values, and the x-axis shows the DTU methods. Each dot in the boxplot indicates the TSES value of one condition pair within a dataset. Methods on the x-axis are ranked by their mean TSES values. **(B) Ranking of DTU methods based on TSES across different datasets.** In the radar chart, a point farther from the center indicates a higher ranking of the corresponding method in that dataset.

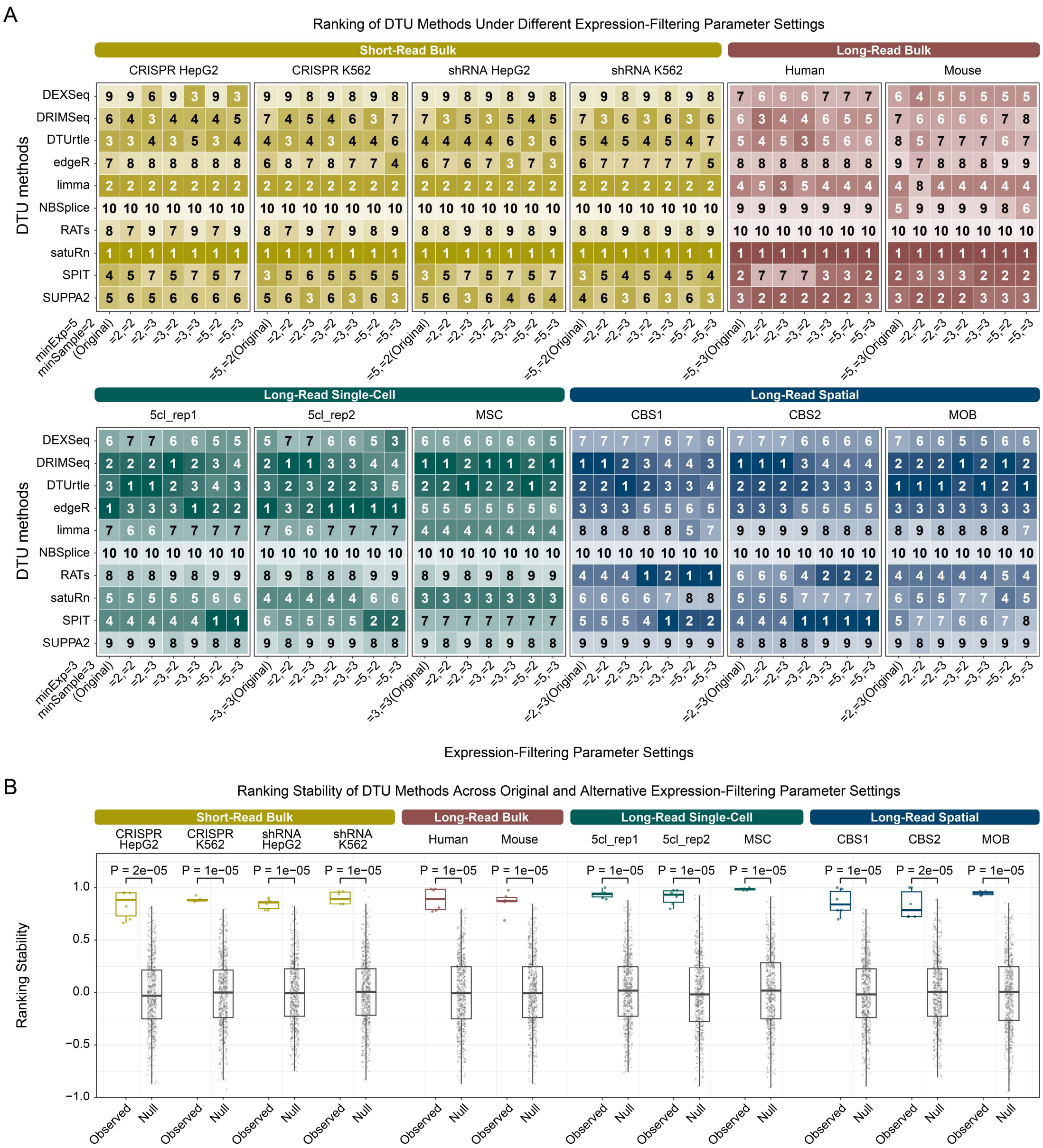

Figure S21. Ranking robustness of ten representative DTU methods under original and alternative expression-filtering parameter settings. (A) DTU method rankings across expression-filtering parameter settings. The heatmap shows the ranks of ten DTU methods under the original and alternative expression-filtering settings (minExp/minSample = 2/2, 2/3, 3/2, 3/3, 5/2, and 5/3) for each dataset. Rows indicate DTU methods, columns indicate expression-filtering settings, and numbers in the tiles indicate method ranks. **(B) Ranking stability relative to the original setting.** Observed ranking stability represents the Spearman correlation between the method ranking under the original setting and the ranking under each alternative setting. Null distributions represent Spearman correlations between randomly permuted method rankings, generated from 100 random permutations for each of the six alternative-setting comparisons (minExp/minSample = 2/2, 2/3, 3/2, 3/3, 5/2, and 5/3), yielding 100 × 6 = 600 null correlations per dataset. P-values are based on one-sided Wilcoxon rank-sum tests for observed values greater than null values.

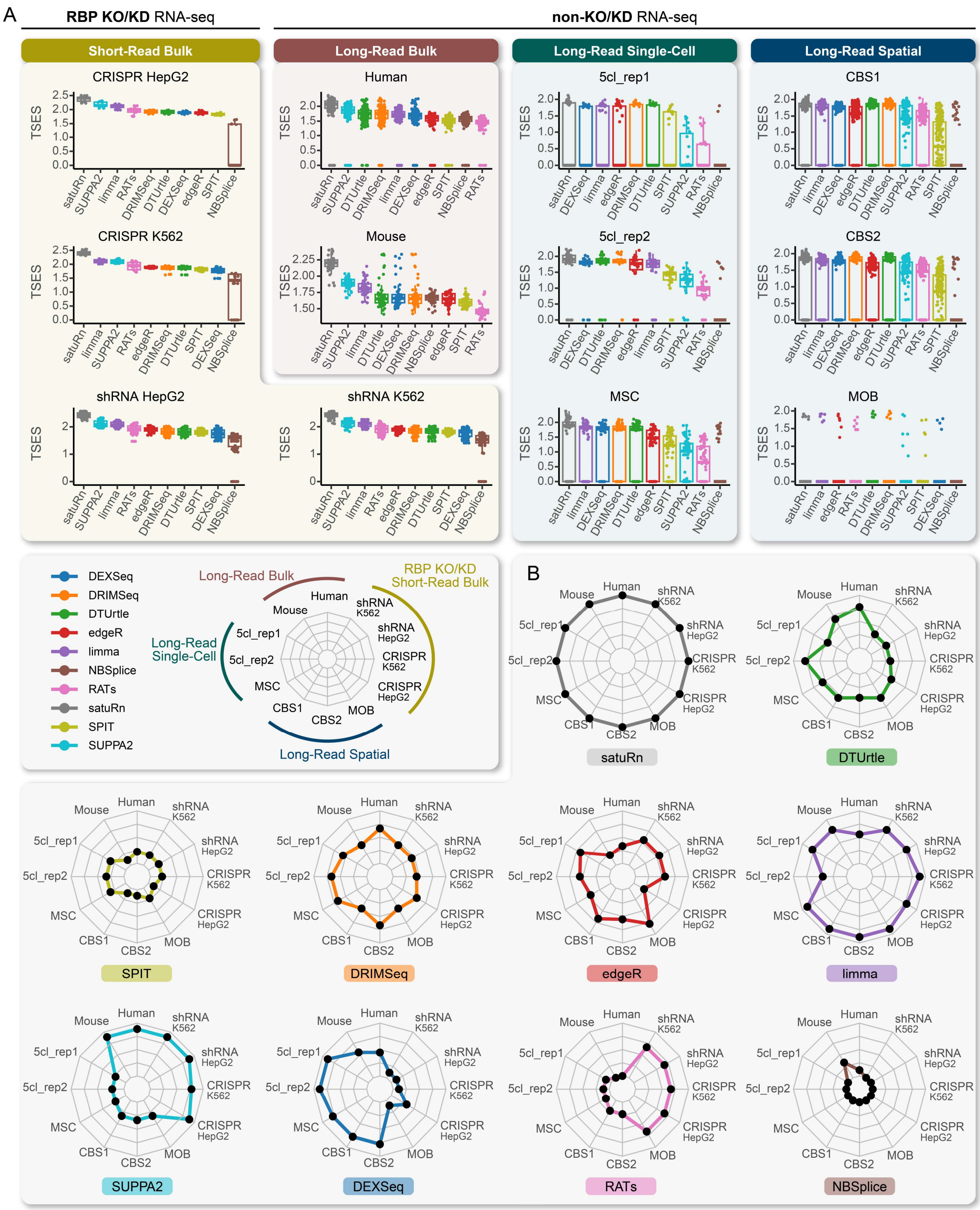

Figure S22. Evaluation results of ten representative DTU methods under the random-subsampling setting (3 vs 3 samples). (A) TSES values of different DTU methods across multiple datasets. The y-axis represents TSES values, and the x-axis shows DTU methods ranked by their mean TSES values within each dataset. Each dot in the boxplot indicates the TSES value of one condition pair in one random subsampling replicate. **(B) Ranking of DTU methods based on TSES across different datasets.** In the radar chart, a point farther from the center indicates a higher ranking of the corresponding method in that dataset.

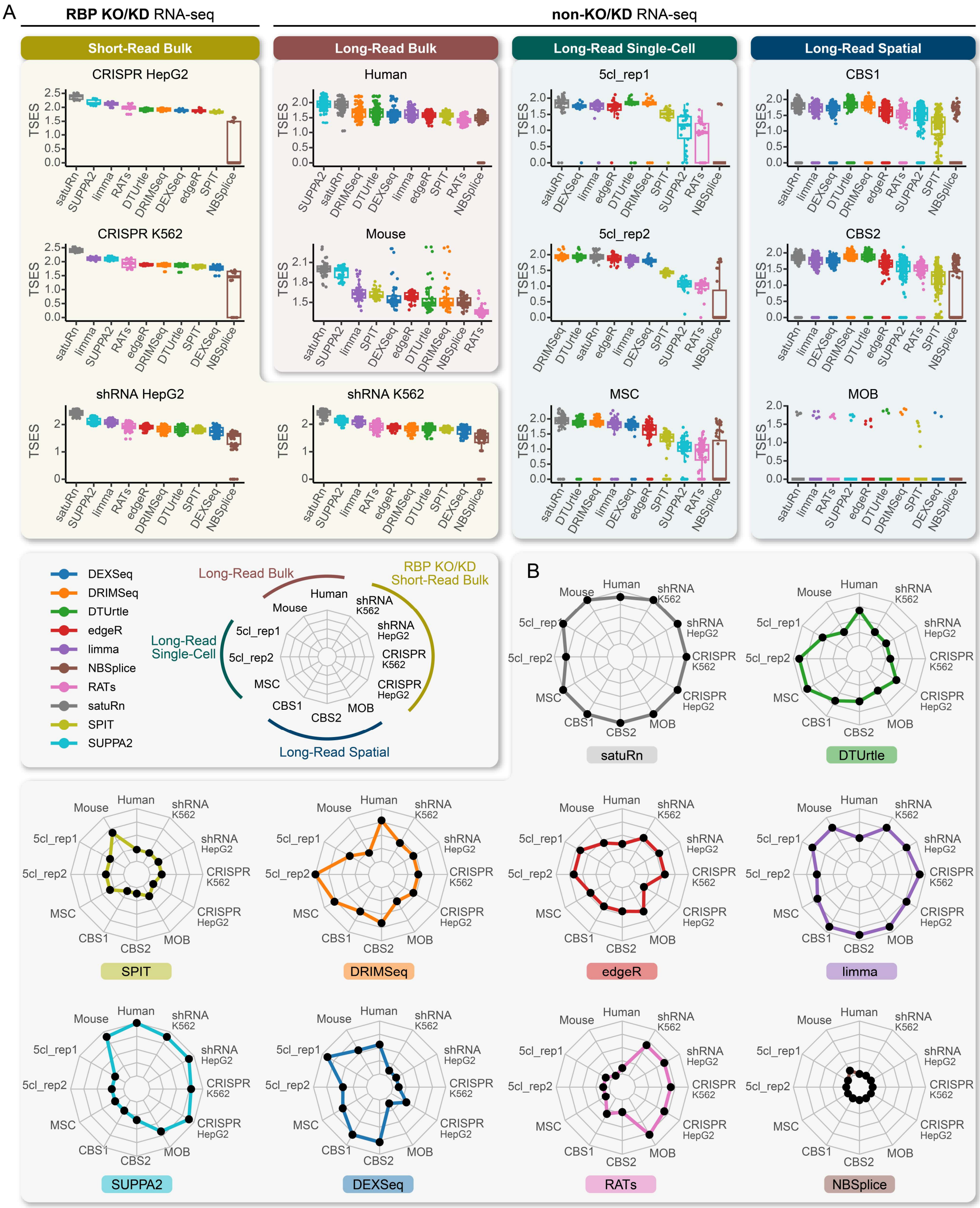

Figure S23. Evaluation results of ten representative DTU methods under the random-subsampling setting (5 vs 5 samples). (A) TSES values of different DTU methods across multiple datasets. The y-axis represents TSES values, and the x-axis shows DTU methods ranked by their mean TSES values within each dataset. Each dot in the boxplot indicates the TSES value of one condition pair in one random subsampling replicate. **(B) Ranking of DTU methods based on TSES across different datasets.** In the radar chart, a point farther from the center indicates a higher ranking of the corresponding method in that dataset.

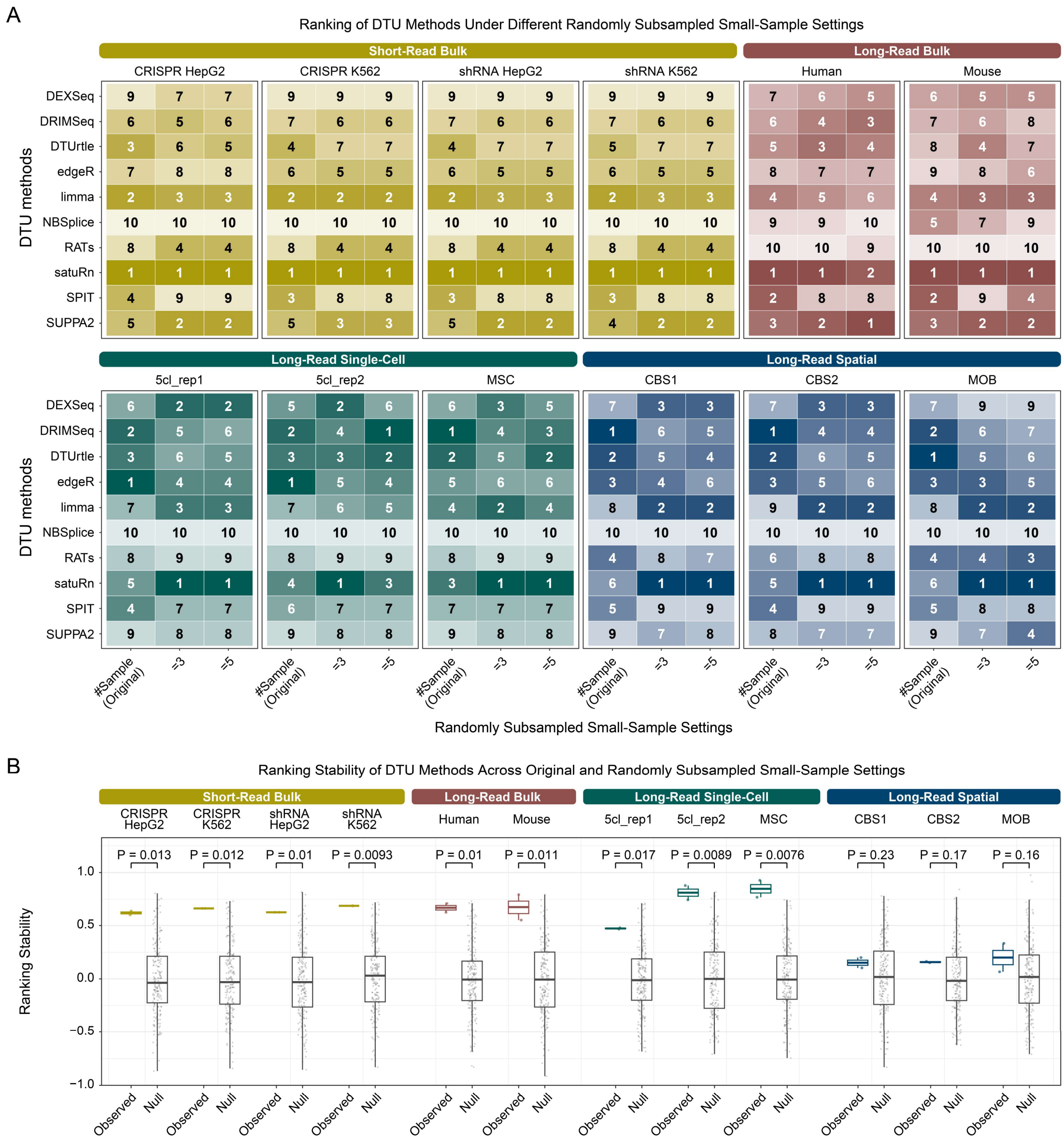

Figure S24. Ranking robustness of ten representative DTU methods under original and random-subsampling setting. (A) DTU method rankings across original and randomly subsampled sample settings. The heatmap shows the ranks of ten DTU methods under the original, 3 vs 3 samples, and 5 vs 5 samples settings for each dataset. Rows indicate DTU methods, columns indicate sample settings, and numbers in the tiles indicate method ranks. Data-type-specific color gradients are used, with darker colors indicating better ranks. **(B) Ranking stability relative to the original setting.** Observed ranking stability represents the Spearman correlation between the method ranking under the original setting and the ranking under each random subsampling setting. Null distributions represent Spearman correlations between randomly permuted method rankings, generated from 100 random permutations for each of the two subsampling settings, yielding 200 null correlations per dataset. Significance is assessed using one-sided Wilcoxon rank-sum tests for observed values greater than null values.

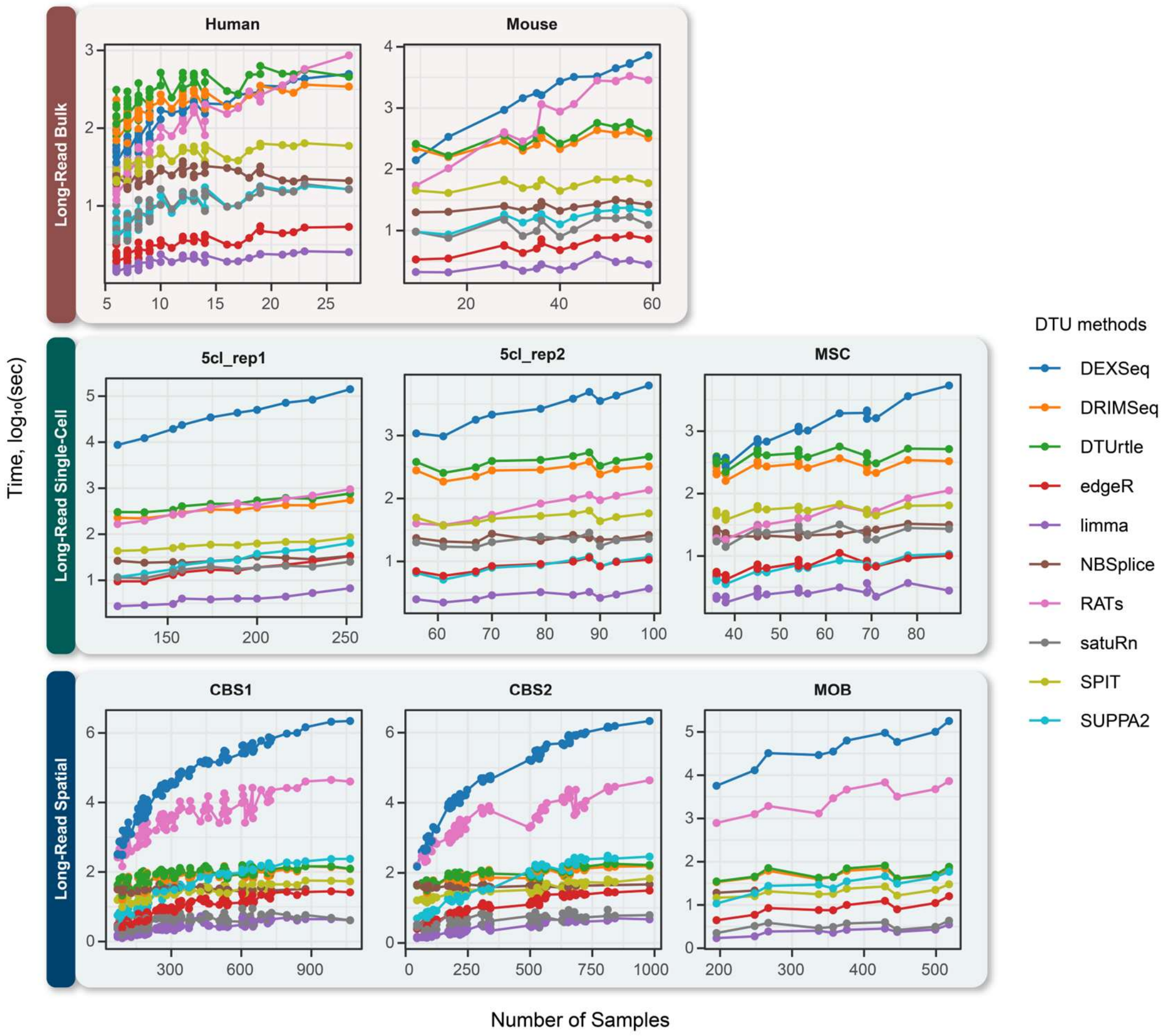

Figure S25. Runtime of DTU methods.

Each point represents one comparison between an experimental group and a control group. The x-axis "Number of Samples" shows the total number of samples from both groups, while the y-axis "Time, $\log_{10}$(sec)" indicates the running time in seconds on a log10 scale.
